# Population sparseness determines strength of Hebbian plasticity for maximal memory lifetime in associative networks

**DOI:** 10.1371/journal.pcbi.1013235

**Published:** 2026-07-06

**Authors:** Naomi Auer, Lars Chen, Jakob Stubenrauch, Benjamin Lindner, Richard Kempter

**Affiliations:** 1 Institute for Theoretical Biology, Department of Biology, Humboldt-Universität zu Berlin, Berlin, Germany; 2 Bernstein Center for Computational Neuroscience Berlin, Berlin, Germany; 3 Department of Physics, Humboldt-Universität zu Berlin, Berlin, Germany; University College London, United Kingdom of Great Britain and Northern Ireland

## Abstract

The brain can efficiently learn and form memories based on limited exposure to stimuli, often even in single trials. Two key factors are believed to support this ability: large synaptic plasticity to strongly encode new memories; and sparse coding, leading to low overlap between memory representations and to small interference. Therefore, increased sparseness can also improve memory capacity. However, it is not well understood how the strength of plasticity of synapses affects capacity. Here, we analyze the combined impact of population sparseness and strength of plasticity on memory capacity. Specifically, we explore how the strength of plasticity that maximizes capacity depends on the sparseness of the neural code. To this end, we study a feedforward network with Hebbian and homeostatic plasticity and a two-state synapse model. The network learns to associate sparse binary input-output pattern pairs. The strength of plasticity is modeled as the probability of synaptic changes. Our results are based on both network simulations and an analytical theory, predicting the expected memory capacity in dependence on strength of plasticity and population sparseness. For both perfect and noisy input patterns, we find that the optimal strength of plasticity increases with increasing pattern sparseness and that this effect is more pronounced for input than for output sparseness. Interestingly, the optimal strength of plasticity remains the same across different network sizes if the number of active units in an input pattern is constant. While the memory capacity obtained at the optimal strength of plasticity increases monotonically with output sparseness, its dependence on input sparseness is non-monotonic. Overall, we provide the first detailed investigation of the interactions between population sparseness, strength of plasticity, and memory capacity. Our findings suggest that differences in sparseness between brain regions may underlie observed differences in how strongly these regions adapt and how quickly they learn.

## Author summary

The brain can efficiently learn and form memories based on limited exposure to stimuli. This ability is believed to be supported by the way neural circuits encode and organize information about the external world. High population sparseness refers to the phenomenon in which, in many brain regions, only a small subset of neurons is active at any given time or in response to a particular stimulus. Such sparse codes are believed to reduce overlap between representations and minimize interference and can thus enhance the storage capacity of a network. Here, we investigate the relation of population sparseness and the strength of plasticity when forming input-output associations in a network model with Hebbian learning. We find that the strength of plasticity that yields the maximal capacity increases with increasing sparseness. The maximal capacity increases for sparser input and output representations, but in the first case only up to a certain point before sparseness becomes destructive. These findings could contribute to explaining why some brain regions learn with higher plasticity than others.

## Introduction

Across many regions of the brain, information seems to be represented by the activity of small fractions of the total number of neurons — in contrast to the limiting cases of single neurons or whole populations of neurons. In particular, the dentate gyrus (DG) is known to be dependent on very sparse population activity; estimates of population sparseness based on the expression of immediate early genes in the DG of rodents propose a percentage of active cells in the one-digit range [1–3]. Other hippocampal subfields, such as Cornu Ammonis 1 (CA1) and Cornu Ammonis 3 (CA3), also use sparse coding, but their activity patterns are believed to be less sparse than in DG [2,4–6]. Outside the hippocampus, the population sparseness has been investigated most extensively in the visual system, because it can explain various visual response properties [7,8]. Estimates for the sparseness level cover a wide range of values, which may be due to different recording techniques, quantification measures, and behavioral paradigms. However, there is a consensus that only a fraction of the neurons available in principle in a particular visual area of the cortex is used to represent a visual stimulus [9–14]. Further, population sparseness has been quantified in the auditory system [15–17], the olfactory system [18–21], the somatosensory system [22], and the amygdala [23]. Insights into the sparseness of the human cortex are limited, but probabilistic reasoning based on single cell recordings predicts a population sparseness of approximately 2% in the human medial temporal lobe [24]. In general, representations appear to become sparser along the hierarchy of sensory processing [8,16,25–28].

In addition to the comprehensive experimental evidence for sparseness in many regions of the brain, there is one main reason why sparse coding is also favorable from a theoretical point of view: the reduced overlap between representations, which brings with it several advantages. Most famously, due to the reduced overlap between patterns, sparse representations can increase the memory capacity of associative neural networks. The dependence of the capacity on the fraction of active neurons was first shown by [29]. Later, [30] found that sparse patterns yield higher capacity than dense patterns also in the Hopfield net [31,32]. In 1987, [33] calculated the theoretical maximum capacity, and soon after [34] proposed a weight configuration that can achieve a capacity close to the theoretical optimum. The beneficial effect of sparseness on the storage capacity of different network structures has been studied extensively since then and has also been confirmed for palimpsest-like networks in which memory capacity is directly determined by memory lifetime [35–42].

Moreover, the reduced overlap and the reduced interference between sparser representations suggest that they are also favorable because they allow for an increased level of plasticity without compromising capacity. This consideration represents the basis for the central hypothesis of this manuscript. In contrast to the well-established increase in storage capacity, higher plasticity as a benefit of sparser coding is hardly discussed in the literature. Although various areas of the brain, in particular the hippocampus, are known for exhibiting sparse representations and rapid learning with high plasticity, the association between these features has not been properly analyzed so far.

In the present paper, we investigate the dependence of the optimal strength of plasticity on the sparseness of representations in a scenario with Hebbian and homeostatic plasticity; furthermore, we explain the combined impact of the strength of plasticity and sparseness on the memory capacity, which is here the same as the memory lifetime, and, in particular, we also determine the maximal memory lifetime. To simplify the approach as much as possible, we use a heteroassociative feedforward network of neurons with binary activations, connected by two-state synapses; the network learns pairs of input-output patterns in a supervised fashion. Based on the underlying assumption that the memory capacity of the network should be maximal, we examine the roles of the (input and output) fractions of active neurons and the probabilities of synaptic changes by means of numerical simulations and analytical derivations. Our theory is based on an extensive pool of work dealing with distributions of dendritic sums and the capacity of Willshaw-like networks (see, e.g., [29,43–52]), which greatly inspired the current study. The advancement of this paper lies in the description of dendritic sums for a strict homeostatic update rule and the quantification of the lifetime-based pattern capacity if the output patterns have a fixed sparseness level, as well as in the application of these findings to an investigation of the relationship between population sparseness, strength of plasticity, and memory lifetime.

This manuscript is organized as follows: We first introduce the model and the learning rule and explain how the capacity of the network is quantified. Then we explain the relationship between the optimal strength of plasticity and the sparseness of both the input and the output patterns. We also show how choosing the optimal strength of plasticity for each sparseness value impacts the maximal memory capacity. Then there is a section that provides a more intuitive understanding of these results based on analytical considerations. Further, we discuss the effect of a change in network size and analyze whether the fraction of active neurons or the absolute number of active neurons is the critical parameter. Finally, we add noise to the input pattern during retrieval in order to generalize our results to the biologically more realistic case of non-perfect retrieval cues. The Results section is followed by the Discussion that sets our results into context. Note that the Results and the Discussion can be read without knowing the methodological details about the theory and the numerical simulations as described in the Methods section.

## Results

The Results described in this section are written so that the text is self-contained. For the interested reader, all further details regarding the theoretical derivations and the numerical simulations used to obtain the results are explained in the Methods section, which is also written in a self-contained way — independent of the Results.

### Network model, learning paradigm, and quantification of capacity

**Fig 1:**
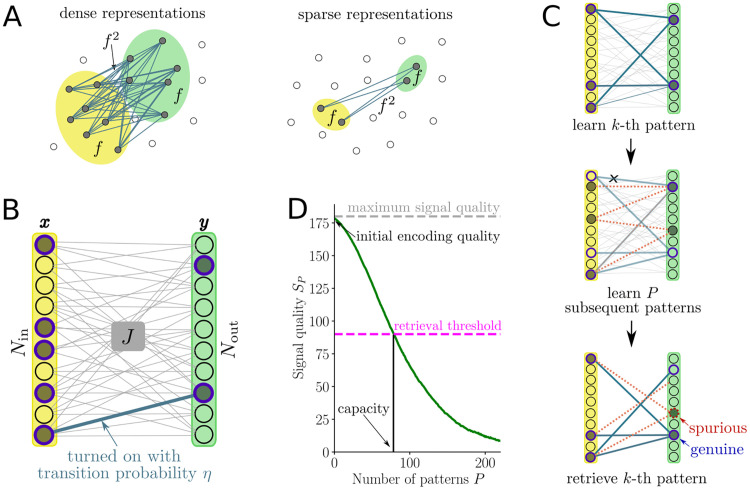
Sparse vs dense representations and quantification of capacity. **(A)** In a network of neurons with an activation ratio *f*, which is the number of neurons in an assembly divided by the total number of neurons in the network, the number of connections potentially involved in an association between two assemblies scales with *f*^2^. The number of relevant connections is thus smaller for sparse representations (left) than for dense representations (right). (**B**) Illustration of the network model with an input layer (yellow) of size *N*_in_ (black circles) with input patterns **x** (indicated by gray discs and blue circles) of activation ratio *f*_in_ and an output layer (green) of size *N*_out_ with output patterns **y** (gray discs and blue circles) of activation ratio *f*_out_ that are connected by a binary weight matrix *J* with a morphological connectivity $c_{m}$ (not sketched) and a functional connectivity *c* (lines between input and output layer) (both normalized per output unit). During learning, the connections between active units are turned on with transition probability η; a homeostasis mechanism maintains the functional connectivity *c*. (**C**) Schematic illustration of the learning and retrieval processes. After learning the *k*-th pattern, *P* additional patterns are learned. In order to determine the memory signal quality of the *k*-th pattern at time *P*, input pattern ${𝐱}^{\left[k\right]}$ is presented to the network again to calculate an output pattern ${\hat{𝐲}}^{\left[k\right]}(P)$, which can then be compared to the target output pattern ${𝐲}^{\left[k\right]}$. (**D**) The quality of the memory signal (signal quality) is defined as $H_{avg}-H({𝐲}^{\left[k\right]},{\hat{𝐲}}^{\left[k\right]}(P))$ (green line). It decays with the number of subsequently stored patterns *P*. The average Hamming distance $H_{avg}$ between two random *f*_out_-sparse vectors of length *N*_out_ (dashed gray line) is an upper bound of the average signal quality. The retrieval threshold is marked as $T_{S}$ (dashed magenta line). The capacity of the network is defined as the number of patterns *P* for which the signal quality reaches the retrieval threshold $T_{S}$.

We are interested in the effect of population sparseness on the optimal strength of plasticity in an associative network. One might hypothesize that sparse representations facilitate a large capacity when there is large synaptic plasticity. This hypothesis is based on the following considerations: In order to establish an association between two neuronal assemblies, the synapses between the neurons that are part of the first assembly and the neurons of the second assembly need to be strengthened. If *f* is the fraction of the total number of neurons that forms an assembly (activation ratio), the number of potential connections between neurons of the two assemblies scales with *f*^2^. A low activation ratio is interpreted as high population sparseness, while a large activation ratio means dense representations and hence a low population sparseness (Fig 1A). On the one hand, learning an association between two sparser assemblies involves only a smaller fraction of synapses. In this case, the memory capacity can be larger because each association affects fewer synapses; however, sparseness cannot be arbitrarily low. On the other hand, an association between denser assemblies involves the modification of a larger fraction of synapses, which can lead to catastrophic interference and lower capacity. For example, for sparser assemblies where only 1% of the cells are active (*f* = 0.01), only a fraction $f^{2}=1/10^{4}$ of the synapses is changed, while for denser assemblies with *f* = 0.5, a quarter (*f*^2^ = 0.25) of all synapses is updated; however, if synaptic plasticity is lower and not all synapses are updated, denser assemblies might generate higher memory capacity. Therefore, to test the hypothesis, the effect of this interaction needs to be quantified.

We thus investigate the interplay between sparseness and plasticity in a heteroassociative one-layer feedforward network with *N*_in_ input and *N*_out_ output units (1B), similar to the architecture of a Willshaw network [29]. This type of hetero-associative network and its storage capacity for both dense and sparse patterns have been of interest for many decades and have been studied extensively [43,44,47,48,52]. The new contribution of our work is an elaboration of the relationship between the sparseness of input and output patterns and the strength of the network’s plasticity as well as their joint effect on the network’s capacity to store memories, especially when this capacity should be as large as possible.

In our setup, a memory is a one-way association between an input pattern **x** and an output pattern **y**. Units have two states and can be either active (1) or inactive (0). The input and output activation ratios *f*_in_ and *f*_out_ are defined as the fraction of active units in the input representation and the output representation, respectively. We denote the number of active input units in an input pattern **x** as $M_{\text{in}}=f_{\text{in}}N_{\text{in}}$; similarly, the number of active output units in a target output pattern **y** is $M_{\text{out}}=f_{\text{out}}N_{\text{out}}$. We typically assume $M_{\text{in}}\gg 1$ and $M_{\text{out}}\gg 1$. Input and output layers of the network are connected by a weight matrix *J* with binary entries. For the construction of *J*, we distinguish between morphological and functional connectivity. In biological neural systems, the number of morphological (or structural) synapses per unit is constrained. Therefore, we assume a morphological connectivity $0<c_{m}\leq 1$. The morphological connectivity is normalized per output unit, and thus each output unit is targeted by exactly $c_{m}N_{\text{in}}$ morphological connections chosen randomly among all possible connections from input units. Entries of the weight matrix *J* that do not correspond to a morphological connection are permanently set to zero. We have a total number of $c_{m}N_{\text{in}}N_{\text{out}}$ morphological connections between the input and the output units, and each connection can be functional (on, value 1) or silent (off, value 0). The weight matrix is initialized such that each output unit is targeted by exactly *cN*_in_ connections that are functional (thin gray lines in Fig 1C top), and we call *c* the functional connectivity. Note that the functional connectivity is bounded by the morphological connectivity: $0<c\leq c_{m}$.

The network learns a sequence of uncorrelated input and output pattern pairs $({𝐱}^{\left[k\right]},{𝐲}^{\left[k\right]}{)}_{k\in \{0,1,\ldots \}}$, which are pairs of randomly drawn binary vectors that meet the sparseness constraint given by *f*_in_ or *f*_out_. Since there is no explicit time component, presenting one pattern pair to the network can be interpreted as one step in time. For each pattern, the weights of the connection matrix *J* are updated in the following way: First, we consider a connection between an active input unit (filled gray circle in yellow layer in Fig 1C top) and an active output unit (filled gray circle in green layer in Fig 1C top). Such a connection can change only if there is a morphological connection. A connection that was already functional stays on, i.e., it is not changed (thin blue line originating from last input unit in Fig 1C top). A connection that was silent is turned on with some transition probability η. These connections are depicted as thick blue lines in Fig 1C top. We interpret the transition probability 0≤η≤1 as the *strength of plasticity* (or *degree of plasticity*) of the network. It is this plasticity parameter that determines how plastic the network connections are. For a high transition probability η, many connections are updated at once, and the memory is strongly engraved into the network. Note that, even though it is often true that higher plasticity leads to more rapid learning, we do not investigate the speed of learning, e.g., in terms of how many trials are needed for a satisfactory outcome (also see Section ‘Strength of plasticity and related concepts’ in the Discussion). Each pattern pair is presented only once and learned in one shot — regardless of the plasticity parameter η.

Furthermore, a homeostasis mechanism maintains the functional connectivity *c*. Connections that were previously functional are turned off in order to balance the additional connections turned on during a learning step. Otherwise, all morphological connections would eventually become functional. In principle, there are many possible ways to normalize the number of functional connections and provide homeostatic stabilization. Inspired by studies suggesting that neurons monitor the total excitation that they receive and that the summed synaptic surface area per length of dendritic segment is conserved over time [53,54], we apply a post-synaptic homeostasis step after each learning step: The functional connectivity *c* is maintained per output unit by randomly silencing the necessary number of connections from inactive input units to active output units. To this end, we must assume $f_{\text{in}}\leq c$ (see Methods, Section ‘Hebbian and homeostatic update rules from a probabilistic perspective’ for details). Connections targeting inactive output units are not changed. This combination of Hebbian potentiation and homeostatic depression realizes a learning rule that is similar to the ones suggested by [36] and by [37]. In summary, the matrix update $J^{\left[k\right]}\to J^{\left[k+1\right]}$ is performed in two steps: First, a Hebbian update is performed with success probability η,


$$\begin{array}{c} K_{ji}^{\left[k+1\right]}=J_{ji}^{\left[k\right]}+{\bar{C}}_{ji}(𝐲{𝐱}^{T}{)}_{ji}(1-J_{ji}^{\left[k\right]})V_{ji}, \end{array}$$ (1)

where $\bar{C}\in \{0,1{\}}^{N_{\text{out}}\times N_{\text{in}}}$ has entries of 1 for predefined morphologically existing connections, thus $\sum_{i}{\bar{C}}_{ji}=c_{m}N_{\text{in}},\forall j$, and $V_{ji}\overset{i.i.d.}{\sim }Bernoulli(\eta )$ is a random binary matrix with independent entries. Second, a random homeostatic step is performed which masks away excess connections,


$$\begin{array}{c} J_{ji}^{\left[k+1\right]}=K_{ji}^{\left[k+1\right]}-{\bar{C}}_{ji}(𝐲(1-𝐱{)}^{T}{)}_{ji}K_{ji}^{\left[k+1\right]}W_{ji}, \end{array}$$ (2)

where the mask $W\in \{0,1{\}}^{N_{\text{out}}\times N_{\text{in}}}$ is a random binary matrix with *N*_out_ constraints $\sum_{i}K_{ji}^{\left[k+1\right]}-{\bar{C}}_{ji}(𝐲(1-𝐱{)}^{T}{)}_{ji}K_{ji}^{\left[k+1\right]}W_{ji}=cN_{\text{in}},\forall j$.

To assess memory capacity, we quantify changes of the memory trace of the *k*-th pattern pair after learning *P* subsequent pattern pairs. With increasing *P*, the memory trace deteriorates more until the memory is no longer retrievable according to some criterion. We always understand the time point *P* as relative to the *k*-th pattern. So after learning the *k*-th pattern, *P* additional patterns (illustrated for *P* = 1 in Fig 1C center; units active in the *k* + 1st pattern are shown as filled gray circles, units active in the *k*-th pattern are shown as circles with dark blue stroke) are learned by turning on connections that are relevant for these patterns and turning others off, potentially also connections that were relevant for the *k*-th pattern. Connections that are turned on in this step are shown as dashed orange lines and connections that are silenced are shown as light blue lines that are crossed out. The network hence realizes a palimpsest memory system [55] where older memories are erased by newer memories due to the homeostasis mechanism.

In order to quantify memory capacity, we follow the line of work started by [46] (see also [45]) but now include not only the plasticity parameter η but also the homeostatic update (cf. Eq (2)). As before, we distinguish two types of output units: Output units are either *genuine* with respect to the *k*-th pattern, hence active in the original target output ${𝐲}^{\left[k\right]}$ or *spurious* with respect to the *k*-th pattern, hence inactive in the target output ${𝐲}^{\left[k\right]}$. During retrieval, there are errors because genuine output units might become inactive and spurious units might become active. At any time step *P* after learning the *k*-th pattern, the retrieval of the *k*-th input-output pattern pair can be tested by presenting the original input pattern and calculating the output with the current weight matrix *J*^[*k*+*P*]^ (Fig 1C bottom). Some of the relevant connections of the *k*-th pattern are still functional (blue lines) and provide input to the genuine output units (blue output units in Fig 1C bottom). Other connections from the same input units might be functional due to learning subsequent patterns (dashed orange lines) or due to the initial weights before learning the *k*-th pattern. These connections provide input to spurious output units (red output unit in Fig 1C bottom).

Output units are modeled as McCulloch-Pitts neurons [56], which sum the weighted inputs and compare them against an activation threshold $T_{\text{in}}^{\left[P\right]}$. Their output is calculated by


$$\begin{array}{c} {\hat{y}}_{j}^{\left[k\right]}(P)=\Theta \left(\left(\sum_{i=1}^{N_{\text{in}}}J_{ji}^{\left[k+P\right]}x_{i}^{\left[k\right]}\right)-T_{\text{in}}^{\left[P\right]}\right) \end{array}$$ (3)

where


$$\begin{array}{c} \Theta (x)=\left\{ \begin{array}{c} 1,\;\text{for }x\geq 0\\ 0,\;\text{for }x<0 \end{array}\right. \end{array}$$ (4)

is the Heaviside step function. The activation threshold $T_{\text{in}}^{\left[P\right]}$ is adjusted in every time step such that the calculated output pattern has the same activation ratio *f*_out_ as the target output. This phenomenologically describes latent competition where $M_{\text{out}}=f_{\text{out}}N_{\text{out}}$ winners take all and the rest is deactivated. Note that this choice of threshold is motivated by the comparability of results based on fixed sparseness values. It is not necessarily an optimal activation threshold in that it maximizes capacity or other performance metrics. The difference between the output ${\hat{𝐲}}^{\left[k\right]}(P)$ obtained with the weight matrix *J*^[*k*+*P*]^ at time (or pattern) *P* and the original target output ${𝐲}^{\left[k\right]}$ of this input pattern is measured by the absolute number of bit flips between ${\hat{𝐲}}^{\left[k\right]}(P)$ and ${𝐲}^{\left[k\right]}$, termed the Hamming distance


$$\begin{array}{cc} H({𝐲}^{\left[k\right]},{\hat{𝐲}}^{\left[k\right]}(P)) & =\sum_{j=1}^{N_{\text{out}}}{\left(y_{j}^{\left[k\right]}-{\left({\hat{y}}^{\left[k\right]}(P)\right)}_{j}\right)}^{2} \end{array}$$ (5)

$$\begin{array}{c} =\#\left\{j\in \{1,\ldots ,N_{\text{out}}\}|y_{j}^{\left[k\right]}\neq {\left({\hat{y}}^{\left[k\right]}(P)\right)}_{j}\right\}. \end{array}$$ (6)

The larger the number of subsequent patterns *P* that have been learned since the one that is being tested for retrieval, the more the obtained output will differ from the target output because the summed inputs to the genuine units become too small compared to the summed inputs of the spurious units.

To quantify the strength of the memory after *P* time steps, we define the signal quality


$$\begin{array}{c} S_{P}:=H_{avg}-H({𝐲}^{\left[k\right]},{\hat{𝐲}}^{\left[k\right]}(P)) \end{array}$$ (7)

where the first term $H_{avg}:=2N_{\text{out}}f_{\text{out}}(1-f_{\text{out}})$ is the average Hamming distance between two random *f*_out_-sparse patterns (Fig 1D, dashed gray line) and the second term is the Hamming distance between the calculated output representation ${\hat{y}}^{[k]}(P)$ and the target ${𝐲}^{\left[k\right]}$ (Fig 1D). For two random *f*_out_-sparse patterns, an error occurs if either the bit of the first pattern is 1 (with probability *f*_out_) and the bit of the second pattern is 0 (with probability $1-f_{\text{out}}$) or the first bit is 0 (with probability $1-f_{\text{out}}$) and the second one is 1 (with probability *f*_out_); the probability for a deviation between the two entries is thus the sum over the probabilities of the two distinct events. Summed over all *N*_out_ entries, this gives *H*_avg_.

We define the memory capacity $P^{*}$ of the network as the lifetime of a memory stored in the network, which is the time that passes until the signal of the memory has decayed to the point that the memory cannot be successfully retrieved anymore. Alternatively, it is the number of patterns that can be stored in addition to the first pattern, without destroying the retrieval of the first pattern. We note that defining capacity in this way has limitations because it does not take into account the information-theoretic efficiency of synapse use [47,52,57] (see ‘Capacity measures’ for a discussion of these limitations). However, a preliminary analysis suggests that the main results of this manuscript are mostly maintained for synaptic (information) capacity (see S8 Appendix).

Practically, the pattern capacity $P^{*}$ here is determined by introducing a retrieval threshold $T_{S}$ (Fig 1D, dashed magenta line), which represents the error tolerance between the obtained output and the target. The number of subsequently stored patterns for which the signal quality reaches the retrieval threshold can be interpreted as the lifetime of the memory or the memory capacity $P^{*}$ of the network, defined by the equation


$$\begin{array}{c} H_{avg}-H({𝐲}^{\left[k\right]},{\hat{𝐲}}^{\left[k\right]}(P^{*}))=T_{S}. \end{array}$$ (8)

We define the threshold


$$\begin{array}{c} T_{S}=t_{S}H_{avg}, \end{array}$$ (9)

with $t_{S}\in (0,1)$, as a fraction of the maximal signal quality, which is the Hamming distance $H_{avg}$ between two random *f*_out_-sparse vectors. A fixed number of wrongly activated units thus has a more severe destructive effect if the total number of active output units $M_{\text{out}}=N_{\text{out}}f_{\text{out}}$ is small. Whenever not further specified, we choose $t_{S}=0.5$ and thus $T_{S}=N_{\text{out}}f_{\text{out}}(1-f_{\text{out}})$.

### Theory on distributions of dendritic sums and calculation of capacity

The memory capacity of the network can be calculated numerically in simulations, but it can also be described analytically in a probabilistic sense. We follow an approach that is similar to the one proposed and promoted by an extensive line of research by Willshaw, Buckingham, Palm, Sommer, Knoblauch, and others (see, e.g., [45–50]) and use the distributions of dendritic sums to calculate the retrieval error, which is expressed by the Hamming distance between the target pattern and the calculated output pattern (see also our Eq (5)). The present work extends and complements previous findings by considering an explicit parameter η for the strength of plasticity. Furthermore, we include a synaptic homeostasis rule that maintains the number of functional connections per output unit *cN*_in_. Both the strength of plasticity and the homeostasis considerably affect the distributions of dendritic sums. Moreover, assuming a fixed output sparseness *f*_out_ affects the placement of the retrieval threshold and makes the quantification of the capacity more involved. Finally, we analyze the role of sparseness for the optimal transition probability and the maximal capacity (see Sections ‘Effect of activation ratio on optimal strength of plasticity and (maximal) capacity’, ‘Role of network size and number of active units’ and ‘Noisy input patterns during retrieval’) and provide an intuitive understanding of the effect of a change in activation ratios on the memory capacity (see Section ‘Activation ratios in the distributions of dendritic sums’).

**Fig 2:**
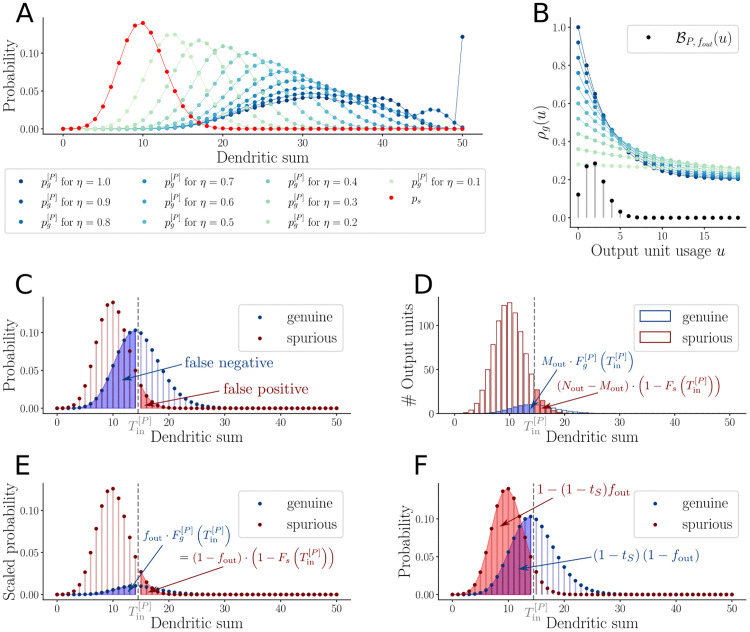
Distributions of dendritic sums and theory on capacity. (**A**) The smaller the transition probability η, the closer the probability mass function (PMF) of the dendritic sums of genuine output units ($p_{g}^{\left[P\right]}$, blue/green) is to the PMF of the dendritic sums of spurious output units ($p_{s}$, red). (**B**) The probability of a functional connection to a genuine output unit decays with the output unit usage *u*. The probability ${ℬ}_{P,\;f_{\text{out}}}(u)$ that an output unit is active *u* times across *P* patterns is the weight of the corresponding binomial PMF in Eq (11). Different shades of blue/green represent different transition probabilities η (same as in (**A**)). In (**A**) - (**B**), *P* = 20. (**C**) Spurious units with a dendritic sum larger than the activation threshold $T_{\text{in}}^{\left[P\right]}$ are wrongly activated (red area, false positive); genuine units with a dendritic sum smaller than $T_{\text{in}}^{\left[P\right]}$ are wrongly not activated (blue area, false negative). (**D**) shows a histogram of the dendritic sums of output units. The expected Hamming distance is the sum of falsely deactivated genuine units (blue area) and falsely activated spurious units (red area). In (**E**), the PMFs of dendritic sums of genuine (blue) and spurious (red) units are scaled by *f*_out_ and $1-f_{\text{out}}$, respectively. To respect the Balance Equation, the mass of the part of the genuine distribution left of the activation threshold $T_{\text{in}}^{\left[P\right]}$ (blue area) must correspond to the mass of the part of the spurious distribution right of $T_{\text{in}}^{\left[P\right]}$ (red area). (**F**) At capacity $P=P^{*}$, the activation threshold $T_{\text{in}}^{\left[P\right]}$ corresponds to the $1-(1-t_{S})f_{\text{out}}$-quantile of the spurious distribution and to the $\left(1-t_{S})(1-f_{\text{out}}\right)$-quantile of the genuine distribution. In (**C**) – (**F**), *P* = 108. Further parameters: $N_{\text{in}}=N_{\text{out}}=1000,f_{\text{in}}=0.05,f_{\text{out}}=0.1,c=0.2,c_{m}=1,t_{S}=0.5$.

Let us now first turn to the definition of dendritic sums, which is very similar to many previous approaches: Given a particular input pattern ${𝐱}^{\left[k\right]}$, the summed input that an output unit receives is called the dendritic sum of this output unit. Output units can potentially be either genuinely or spuriously active in the calculated output ${\hat{𝐲}}^{\left[k\right]}(P)$ depending on whether they are active or inactive in the target output ${𝐲}^{\left[k\right]}$. For each output unit, the dendritic sum hence follows either the distribution of dendritic sums of spurious output units (red distribution in Fig 2A, will be called *spurious distribution*) or the distribution of dendritic sums of genuine output units (blue/green distributions in Fig 2A, will be called *genuine distribution*).

The probability mass function (PMF) of the dendritic sum of a spurious unit is


$$\begin{array}{c} p_{s}(x)={ℬ}_{M_{\text{in}},\;c}(x) \end{array}$$ (10)

(see Fig 2A red, derived in Methods, Section ‘Probabilistic description of dendritic sums’ and S1 Appendix, see Eqs (36) and (40)), where ${ℬ}_{n,p}$ denotes a binomial distribution with *n* trials and success probability *p*. The spurious distribution is centered at *M*_in_*c* where $M_{\text{in}}=N_{\text{in}}f_{\text{in}}$. We note that *M*_in_*c* remains constant when *P* is changed due to the constant functional connectivity *c* that ensures a fixed number of functional connections per output unit; when a random input pattern is applied, on average, a fraction *f*_in_ of the *cN*_in_ connections receives input. The spurious distribution does not change when new uncorrelated patterns are learned, and thus the spurious distribution $p_{s}$ does not depend on the number of patterns *P*.

In contrast, the PMF of the distribution of the dendritic sums of genuine units depends on *P*; it is given by


pg[P](x)=∑u=0P[(Pu)foutu(1−fout)P−u·ℬMin,ρg(u)(x)] (11)

(see Fig 2A blue/green) where the probability $\rho_{g}$ for a connection to a genuine unit to be functional given that the output unit is active *u* times across the whole pattern set is


$$\begin{array}{c} \rho_{g}(u)=(c_{m}-c)\eta {\left(1-\frac{f_{\text{in}}\eta c_{m}}{c}\right)}^{u}+c \end{array}$$ (12)

(see Fig 2B blue/green, derived in Methods, Section ‘Probabilistic description of dendritic sums’ and S1 Appendix, see Eqs (36) and (41)). Immediately after learning a specific pattern, the probability of the relevant connections to a genuine output unit to be functional is larger than the average functional connectivity *c*, i.e., in Eq (12) we have $\rho_{g}(0)=(c_{m}-c)\eta +c$. Due to the homeostatic plasticity, the probability $\rho_{g}$ is reduced every time this output unit is active in subsequent patterns, which is reflected in the term ${\left(1-\frac{f_{\text{in}}\eta c_{m}}{c}\right)}^{u}$ where *u* is the output unit usage. For u→∞, we have $\rho_{g}(u)\to c$. The probability $\rho_{g}$ enters Eq (11) in ${ℬ}_{M_{\text{in}},\;\rho_{g}(u)}(x)$, which is the distribution of the dendritic sums if the output unit was active *u* times in the other patterns. The distribution of the output unit usage *u*, hence the number of times that an output unit is active across a set of *P* patterns, explains the weight (Pu)foutu(1−fout)P−u of a binomial distribution in Eq (11) (see Fig 2B black). For P→∞, the genuine distribution converges to the spurious distribution. The distributions of dendritic sums obtained from numerical simulations match the distributions in Eqs (10) and (11) very well (see the example in S5 Fig).

For an analytical description of the expected Hamming distance $𝔼(H({𝐲}^{\left[k\right]},{\hat{𝐲}}^{\left[k\right]}(P)))$ between a target output pattern ${𝐲}^{\left[k\right]}$ and the corresponding output pattern ${\hat{𝐲}}^{\left[k\right]}(P)$ calculated during retrieval, we need to calculate the expected number of output neurons that are in the wrong state in ${\hat{𝐲}}^{\left[k\right]}(P)$ compared to ${𝐲}^{\left[k\right]}$. We note that the output activation ratio of the calculated output pattern should be the same as the activation ratio *f*_out_ of the target output pattern. The activation threshold *T*_in_ should be chosen accordingly. This constraint is different from other works (e.g., [47,58,59]) and provides an additional challenge to the derivation of the storage capacity (for example, Eq (15) needs to be respected; see below and Methods, Section ‘Analytical Hamming distance’ for more details). The activation threshold *T*_in_ (vertical dashed gray line in Fig 2C) determines which output units are activated and which are not. If the distributions of dendritic sums of genuine and spurious output units are far apart and do not overlap, which can be the case during the first steps after learning a pattern, hence for small *P*, the activation threshold *T*_in_ can perfectly separate the two distributions, and then the calculated output is identical with the target output. If the two distributions are close to each other, a part of the spurious output units is wrongly activated (red area under red distribution in Fig 2C, false positive) and a part of the genuine output units is wrongly inactivated (blue area under blue distribution in Fig 2C, false negative). These two errors both contribute to the Hamming distance between the target output pattern and the calculated output pattern but they are weighted differently, as will be explained in the following (see Methods for a detailed derivation). In order to derive an actual error contribution from these distributions, we have to consider how many units have dendritic sums following either of the two distributions. As mentioned, there are *M*_out_ active units in the target output pattern. There are hence *M*_out_ output units whose dendritic sum follows the genuine distribution and $N_{\text{out}}-M_{\text{out}}$ output units whose dendritic sum follows the spurious distribution. The false negative error and the false positive error thus have to be weighted by *M*_out_ and $N_{\text{out}}-M_{\text{out}}$, respectively. It follows that the expected Hamming distance can be expressed as


$$\begin{array}{c} 𝔼\left(H\left({𝐲}^{\left[k\right]},{\hat{𝐲}}^{\left[k\right]}(P)\right)\right)=M_{\text{out}}e_{\text{fn}}+(N_{\text{out}}-M_{\text{out}})e_{\text{fp}}, \end{array}$$ (13)

where *e*_fn_ and *e*_fp_ denote the error probabilities due to false negatives (blue area) and false positives (red area), respectively.

Let $F_{g}^{\left[P\right]}$ and $F_{s}$ denote the cumulative distribution functions (CDF) of the distributions of dendritic sums of genuine units and spurious units, respectively. Then, we can express the expected Hamming distance as


$$\begin{array}{c} 𝔼\left(H\left({𝐲}^{\left[k\right]},{\hat{𝐲}}^{\left[k\right]}(P)\right)\right)=M_{\text{out}}F_{g}^{\left[P\right]}\left(T_{\text{in}}^{\left[P\right]}\right)+(N_{\text{out}}-M_{\text{out}})\left(1-F_{s}\left(T_{\text{in}}^{\left[P\right]}\right)\right), \end{array}$$ (14)

see Fig 2D (derived in Methods).

As discussed in the previous section, we always adjust the activation threshold $T_{\text{in}}^{\left[P\right]}$ such that the output activation ratio of the target output pattern ${𝐲}^{\left[k\right]}$ is maintained in the calculated output ${\hat{𝐲}}^{\left[k\right]}(P)$. This restriction is respected if the Balance Equation


$$\begin{array}{c} f_{\text{out}}\cdot F_{g}^{\left[P\right]}\left(T_{\text{in}}^{\left[P\right]}\right)=(1-f_{\text{out}})\cdot \left(1-F_{s}\left(T_{\text{in}}^{\left[P\right]}\right)\right) \end{array}$$ (15)

is fulfilled (see Fig 2E), i.e., if the contribution to the Hamming distance of the wrongly inactive genuine units $M_{\text{out}}e_{\text{fn}}$ equals the contribution of the wrongly active spurious units $(N_{\text{out}}-M_{\text{out}})e_{\text{fp}}$ (see derivation in Methods).

With the Balance Equation, we can express $F_{g}^{\left[P\right]}\left(T_{\text{in}}^{\left[P\right]}\right)$ in terms of $F_{s}\left(T_{\text{in}}^{\left[P\right]}\right)$, and the expected Hamming distance simplifies to


$$\begin{array}{c} 𝔼\left(H\left({𝐲}^{\left[k\right]},{\hat{𝐲}}^{\left[k\right]}(P)\right)\right)=2N_{\text{out}}(1-f_{\text{out}})\left(1-F_{s}\left(T_{\text{in}}^{\left[P\right]}\right)\right). \end{array}$$ (16)

To calculate the capacity, we need to determine the number of patterns *P* for which the signal quality $S_{P}=H_{\text{avg}}-H\left({𝐲}^{\left[k\right]},{\hat{𝐲}}^{\left[k\right]}(P)\right)$, as defined in Eq (7), equals the retrieval threshold $T_{S}=t_{S}H_{\text{avg}}$ (see Methods for details), i.e., we have to solve the equation


$$\begin{array}{c} H_{\text{avg}}-𝔼\left(H\left({𝐲}^{\left[k\right]},{\hat{𝐲}}^{\left[k\right]}(P)\right)\right)=t_{S}H_{\text{avg}}, \end{array}$$ (17)

which can be simplified to


$$\begin{array}{c} f_{\text{out}}(1-t_{S})=1-F_{s}\left(T_{\text{in}}^{\left[P\right]}\right). \end{array}$$ (18)

Using this relation in the form $F_{s}\left(T_{\text{in}}^{\left[P\right]}\right)=1-f_{\text{out}}(1-t_{S})$ in the Balance Equation yields


$$\begin{array}{c} F_{g}^{\left[P\right]}\left(T_{\text{in}}^{\left[P\right]}\right)=(1-t_{S})(1-f_{\text{out}}). \end{array}$$ (19)

We now have two equations, Eq (18) and Eq (19), in two variables *P* and $T_{\text{in}}^{\left[P\right]}$. Solving them gives us the number of patterns *P* (and the corresponding activation threshold $T_{\text{in}}^{\left[P\right]}$) for which the signal quality $S_{P}$ equals the retrieval threshold. This *P* is the capacity (or memory lifetime) $P^{*}$.

Since the dendritic sums follow discrete distributions (see Eqs (10) and (11)), their CDFs $F_{g}^{\left[P\right]}$ and $F_{s}$ are not invertible over a continuous domain. If we approximate the distributions by appropriate continuous distributions, Eqs (19) and (18) can be reduced to a single equation


$$\begin{array}{c} F_{s}^{-1}\left(1-(1-t_{S})f_{\text{out}}\right)={F_{g}^{\left[P^{*}\right]}}^{-1}\left((1-t_{S})(1-f_{\text{out}})\right) \end{array}$$ (20)

in $t_{S}$. The capacity $P^{*}$ corresponds thus to the number of patterns *P* for which the $1-(1-t_{S})f_{\text{out}}$-quantile of the spurious distribution equals the $\left(1-t_{S})(1-f_{\text{out}}\right)$-quantile of the genuine distribution (Fig 2F). An analytical solution to Eq (20) is derived in the Methods.

### Effect of activation ratio on optimal strength of plasticity and (maximal) capacity

Let us now quantify how the memory capacity $P^{*}$ depends on the activation ratio of the input pattern *f*_in_, the activation ratio of the output pattern *f*_out_, and on the transition probability η. While many earlier works (e.g., [29,38,43,44,46]) have already analyzed how activation ratios impact the capacity of similar networks, we extend these earlier works by adding the strength of plasticity as an additional parameter. We can thus investigate the new triangular relationship between activation ratios, strength of plasticity, and capacity. This allows us to derive and discuss an *optimal* strength of plasticity and to define a *maximal* capacity.

**Fig 3:**
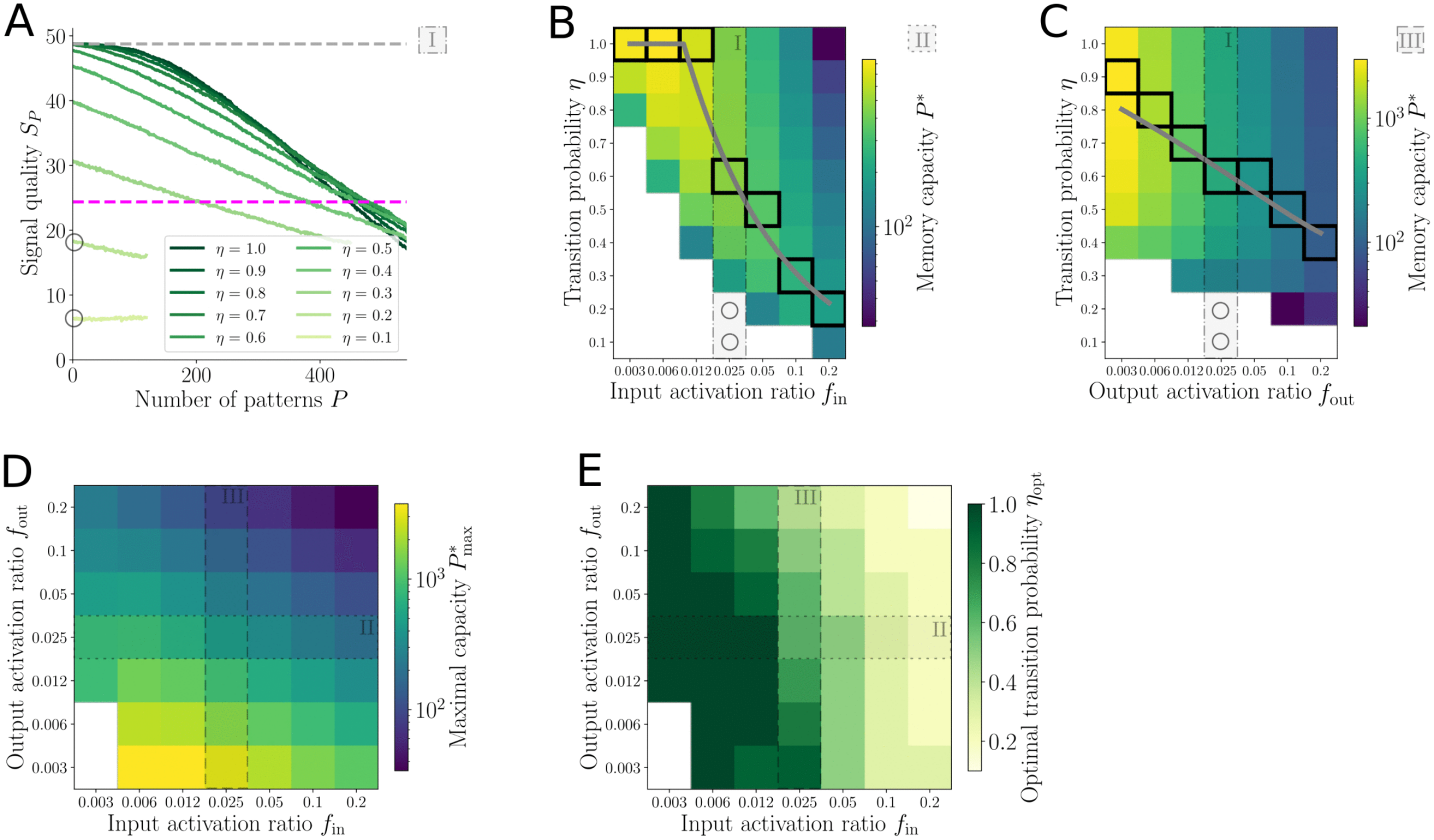
Effect of activation ratio on optimal strength of plasticity and capacity. (**A**) Signal quality $S_{P}$ decaying with number of patterns *P* for various transition probabilities η. While small η cause low initial signal quality, large η cause a fast decay. A transition probability of η=0.6 yields the largest memory capacity because it reaches the retrieval threshold (magenta dashed line), which is chosen as half of the maximal signal quality (gray dashed line), for the largest number of patterns *P*. For approximately η<0.25, the initial signal quality is lower than the threshold and the capacity is zero (gray circles, see also gray circles in (**B**) and (**C**)). Fixed parameters: $f_{\text{in}}=f_{\text{out}}=0.025$. (**B**) For a given transition probability η≤1, the memory capacity increases with decreasing input activation ratio *f*_in_ until it reaches a maximum and then quickly decays to zero for smaller *f*_in_. The optimal η, which is defined as the transition probability that results in the largest memory capacity for a given *f*_in_, increases with decreasing *f*_in_ until it reaches and stays at its largest value of 1 (black squares). The solid gray line shows the analytically derived optimal η as a function of *f*_in_. The signal-quality traces of the column marked by ‘I’ are shown in (**A**). White area indicates a capacity of zero. Fixed parameter: *f*_out_ = 0.025. (**C**) Same as (**B**) for output activation ratio *f*_out_ instead of input activation ratio *f*_in_. The memory capacity and the optimal η increase with decreasing *f*_out_. Column ‘I’ is shown in (**A**). Fixed parameter: *f*_in_ = 0.025. In (**D**), the maximal capacity (obtained with the optimal transition probability $\eta_{opt}$ shown in (**E**)) for each combination of input activation ratio *f*_in_ and output activation ratio *f*_out_ is shown. For a fixed *f*_in_, the maximal capacity increases monotonically with decreasing *f*_out_. For a fixed *f*_out_, the maximal capacity has a maximum at a small but nonzero *f*_in_. Columns ‘II’ and ‘III’ are obtained from the results shown in (**B**) and (**C**), respectively. (**E**) Optimal transition probability $\eta_{opt}$ increases with decreasing *f*_in_ and with decreasing *f*_out_. The slope is larger for *f*_in_ than *f*_out_. Columns ‘II’ and ‘III’ are shown in (**B**) and (**C**), respectively. Further fixed parameters in (**A**) -(**E**): $N_{\text{in}}=1000,N_{\text{out}}=1000,c=0.2,c_{m}=1,t_{S}=0.5$, *N*_avg_ = 200 (see Methods for details on averaging).

Fig 3A shows the signal quality as a function of the number of patterns *P* for several transition probabilities η. The signal quality always decays with time, i.e., with the number *P* of subsequently stored patterns. The larger the transition probability, the faster the decay because the number of connections that is updated in each time step is larger and the old memories are thus overwritten more quickly. In the long run, the signal quality decays to zero. The initial signal quality (shown at *P* = 0 in Fig 3A) strongly depends on the transition probability because it determines how many connections are used for the initial memory storage. For a higher transition probability, the memory is more strongly engraved into the system and, initially, can be retrieved better. If the transition probability is too low, too few connections are updated to recall a memory even right after storing it (gray circles in Fig 3A). The transition probability that yields the highest capacity, which we call the optimal transition probability, hence depends on a trade-off between a high initial encoding and a slow decay of the memory signal (Fig 3A).

Fig 3B summarizes how the memory capacity $P^{*}$ depends on the input activation ratio *f*_in_ and the transition probability η — for fixed *f*_out_. The capacity values of Fig 3A are visible in the marked column (*f*_in_ = 0.025) of Fig 3B. In each column of Fig 3B, hence for a fixed *f*_in_, the largest capacity value that thus corresponds to the optimal transition probability is marked by a black square. The corresponding analytical estimate of the optimal transition probability is displayed by the gray line (Eq (103) in Methods, discussed in Section ‘Role of network size and number of active units’). For a fixed transition probability η (single row in Fig 3B), the capacity $P^{*}$ is non-monotonic as a function of *f*_in_, i.e., it increases with increasing input activation ratio *f*_in_ and then decreases again. As known from the literature [34,36,38], denser representations (higher *f*_in_) can lead to lower capacity values. However, if the input representations become too sparse (too small *f*_in_), the maximal possible capacity also drops and goes to zero for very sparse input patterns. This non-monotonic dependence of the capacity on the sparseness of the patterns has also been known for decades [37,38,45,52,60] and is confirmed here. The drop in capacity for very small input activation ratios can be explained by the fact that it is not possible for too few active input units to sufficiently excite the respective output units. In this case, a slight change of the weight matrix due to the storage of additional patterns can lead to very different units being activated in the output. In extreme cases — especially if both the transition probability η and the input activation ratio *f*_in_ are too small — the pattern is not well enough imprinted from the very beginning (cf. Fig 3A, gray circles, and 3B, white region) and the capacity is zero. This issue is particularly severe if the error tolerance $1-t_{S}$ is small, hence if the retrieval threshold $T_{S}$ is high, i.e., is close to its maximum *H*_avg_. In this case, the capacity can be zero even for high transition probabilities η if the initial signal quality lies below $T_{S}$. In the following section ‘Activation ratios in the distributions of dendritic sums’, we provide an intuitive understanding of the role of the input activation ratio in the distributions of dendritic sums and its impact on the capacity.

Fig 3C summarizes how the memory capacity $P^{*}$ depends on the output activation ratio *f*_out_ and the transition probability η — for fixed *f*_in_. The marked column in Fig 3C shows the same capacity values as the marked column in Fig 3B. For a constant transition probability (single rows in Fig 3C), we find that the capacity increases with decreasing output activation ratio *f*_out_. A smaller output activation ratio *f*_out_ is beneficial for the capacity because a specific output unit is less likely to be active in subsequent patterns; hence relevant connections are turned off with lower probability. Nevertheless, if the transition probability η is too small, as before, the memory is not sufficiently well imprinted from the beginning and the capacity is zero (Fig 3C, white region). Previous work showed that, due to a collapse of the retrieval reliability for high output sparseness as soon as some noise is present in the system, also the output activation ratio can have a non-monotonic effect on the capacity in that capacity decreases again if the activation ratio becomes too small [46,52,60]. However, in the parameter ranges investigated in this study, the capacity always showed a monotonic decrease with the output activation ratio. For an attempt at an intuitive explanation of the dependence of the capacity on the output sparseness via an analysis of the role of the output activation ratio in the distributions of dendritic sums, we refer again to the following section ‘Activation ratios in the distributions of dendritic sums’.

In summary, we find that in our network the sparseness values of the input and the output patterns have differential effects on the capacity of the network. While the smallest output activation ratio achieves the highest capacity values (Fig 3C), there is a non-monotonic dependence of the capacity on the input activation ratio (Fig 3B).

While capacity as a function of sparseness has been extensively investigated in earlier work (e.g., [34,37,38,45,52,60]), we can, in our setup, now extend these dependencies to the *maximal* capacity obtained with the *optimal* strength of plasticity: The non-monotonic dependence of the capacity on *f*_in_ and its monotonic dependence on *f*_out_ are also maintained for the maximal capacity. Fig 3D summarizes the maximal capacity for various pairs of input and output activation ratios *f*_in_ and *f*_out_, which is obtained by using the optimal transition probability for each pair. As for the capacity for a fixed η in Fig 3B,3C, Fig 3D shows an asymmetric effect of *f*_in_ and *f*_out_ on the maximal capacity.

Finally, the quantification of the capacity in dependence of the strength of plasticity allows us to derive the *optimal* strength of plasticity, which is the transition probability that yields the largest capacity — for fixed input and output activation ratios. A central result of our study is that the optimal strength of plasticity depends on the input and output activation ratios. The sparser the patterns (smaller *f*_in_ or *f*_out_), the larger the transition probability that allows for the largest capacity (depicted by black squares in Fig 3B,3C). This effect is monotonic and has a larger gradient for the input activation ratio (Fig 3B) than for the output activation ratio (Fig 3C). This behavior is summarized in Fig 3E for a wider range of pairs of *f*_in_ and *f*_out_.

In S6 Appendix, we compare our results that were obtained with a homeostasis based post-synaptic normalization to results obtained for a pre-synaptic normalization and for a normalization averaged over all connections. We find that the qualitative dependencies of the optimal transition probability and the maximal capacity on the activation ratios (compare Fig 5D,5E to S6 Appendix) remain unchanged.

### Activation ratios in the distributions of dendritic sums

While the benefit of sparseness for the capacity for large to moderate activation ratios and the decrease of capacity for small activation ratios (in particular small input activation ratios) were already well-established in the literature (e.g., [37,38,45,52,60]), we provide here a new intuitive analysis of these effects based on the distributions of dendritic sums.

The intuitive understanding of the effects of the input and the output activation ratios on the capacity can be strengthened by having a closer look at the distributions of the dendritic sums, which determine the signal quality of the memory. We again distinguish distributions of dendritic sums of spurious and genuine units (red and blue/green, respectively, in Fig 2A) as given in Eq (10) and Eq (11), respectively. These explicit expressions for dendritic sums of spurious and genuine units allow us to understand why *f*_in_ and *f*_out_ affect the capacity of the network in different ways.

**Fig 4:**
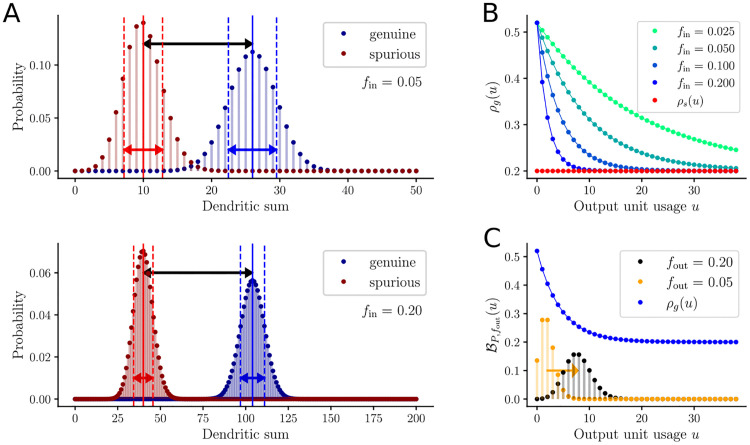
Role of the activation ratios in the distributions of dendritic sums. (**A**) Distributions of dendritic sums of genuine (blue) and spurious (red) output units. In the bottom panel, the input activation ratio *f*_in_ is four times larger than in the top panel. For a larger *f*_in_, the difference between the means of the distributions (black arrows) is larger (note the different scales of the *x*-axes in the two panels). The standard deviations (red and blue arrows) are also larger for larger *f*_in_ but the coefficients of variation are smaller. Also the overall overlap of the two distributions is smaller for larger *f*_in_. (**B**) The probability $\rho_{g}$ of a connection to a genuine output unit to be functional as a function of the output unit usage *u*. $\rho_{g}(u)$ always decays as a function of *u* and approaches $\rho_{s}$ (red) for large *u*. The decay is faster for larger input activation ratio *f*_in_. (**C**) The weight distribution ${ℬ}_{P,f_{\text{out}}}$ moves to the right if *f*_out_ is increased (black: *f*_out_ four times larger than in orange). The largest weights hence affect binomials ${ℬ}_{M_{\text{in}},\rho_{g}(u)}$ (in Eq (11)) with smaller $\rho_{g}(u)$ values (blue). Further parameters: $N_{\text{in}}=1000,f_{\text{out}}=0.1,c=0.2,c_{m}=1,P=0,\eta =0.4$.

We first explain the role of *f*_in_, while *f*_out_ is assumed to be fixed: A fixed *f*_out_ implies that the weighting of the individual binomial mass functions in Eq (11) is fixed for a given *P*. We can hence discuss the role of *f*_in_ in the individual binomials without considering the weighting. The capacity’s non-monotonic dependence on *f*_in_ is a combination of two separate effects working together. In the following, we discuss via what dominating mechanism *f*_in_ can positively correlate with the capacity, which is the case for very small *f*_in_ (left of the gray line in Fig 3B), and what mechanism has to dominate such that *f*_in_ is negatively correlated with the capacity, which is the case for larger *f*_in_ (right of the gray line in Fig 3B). First, the input activation ratio occurs in the number of trials $M_{\text{in}}=f_{\text{in}}N_{\text{in}}$ of the individual binomial distributions in $p_{g}^{\left[P\right]}$ and the number of trials *M*_in_ of the spurious distribution $p_{s}$. Let us assume for the moment that *f*_in_ was increased only in the number of trials of the binomials, and we ignore the dependence of $\rho_{g}$ on *f*_in_ in Eq (12). Such an increase of *f*_in_ in the number of trials pulls the distributions $p_{s}$ and $p_{g}^{\left[P\right]}$ farther apart since both mean values are proportional to *f*_in_. They are also stretched out more (higher standard deviation $\sqrt{M_{\text{in}}p(1-p)}$ with *p* = *c* or $p=\rho_{g}(u)$) but the overall overlap of the two distributions is reduced because the means grow with *M*_in_ and the standard deviations grow only with $\sqrt{M_{\text{in}}}$ (also see left hand side of Eq (91) for *P* = 0). This is illustrated in Fig 4A (top: smaller *f*_in_, bottom: larger *f*_in_). Due to the smaller overlap of the two distributions, the Hamming distance is reduced and this yields a larger capacity. For *f*_in_ increasing from zero, this increase of the capacity can be seen for the values left of the gray line in Fig 3B. However, the input activation ratio *f*_in_ also occurs in the probabilities $\rho_{g}(u)$ (Eq (12)) of the individual binomials in the genuine distributions. If only changed at this place, an increased *f*_in_ leads to a faster decay of the probability with increasing *u* (Fig 4B) and hence with the number of patterns *P*. For larger *f*_in_, each of the individual binomials ${ℬ}_{M_{\text{in}},\;\rho_{g}(u)}$ in Eq (11) and hence also the total genuine distribution converges faster to the spurious distribution, which implies a deteriorative effect on the capacity. This can be seen for the capacity values right of the gray line in Fig 3B. In summary, these two effects dominate in different ranges of *f*_in_, which can explain the intermediate value of *f*_in_ that yields the optimal capacity. For input activation ratios *f*_in_ close to zero, the effect of the change of *f*_in_ in the number of trials of the distributions (10) and (11) dominates over the effect of the change of *f*_in_ in Eq (12). The capacity hence increases with increasing *f*_in_ if *f*_in_ is small (derived in S5 Appendix). This confirms the detrimental effect of a very small *f*_in_ on the network capacity that we discussed in the previous section: If *f*_in_ becomes very small, the number of active input units becomes too low to reliably activate the respective output units. For very large input activation ratios *f*_in_, the effect of a change of *f*_in_ via the probability $\rho_{g}$ (Eq (12)) is stronger than the effect via the number of trials and the capacity decreases with increasing *f*_in_.

The upper limit of *f*_in_ is *c* (see first section on condition $f_{\text{in}}\leq c$). The capacity for this upper limit of the input activation ratio *f*_in_ can be derived for the special case $c_{m}=1,\eta =1$. If *f*_in_ takes its maximal value *c*, the probability $\rho_{g}(u)$ (Eq (12)) equals *c* and the genuine (Eq (11)) and spurious distributions (Eq (10)) become identical. This makes it impossible to differentiate between genuine and spurious units and the capacity is hence zero. In total, the intuition that we have developed here thus matches the numerical results discussed in the previous section.

Let us now turn to the output activation ratio *f*_out_, which affects the capacity also in two ways. We now assume a fixed *f*_in_. First, *f*_out_ is the success probability of the binomial distribution, which constitutes the weights in the genuine distribution (Eq (11)). An increased *f*_out_ pushes the center of mass of the distribution to larger values of *u* (Fig 4C). The largest weights thus are given to larger *u* values, which correspond to binomials with smaller probabilities $\rho_{g}(u)$. This emphasizes the parts of $p_{g}^{\left[P\right]}(x)$ that are more to the left of $x\in [0,M_{\text{in}}]$, which culminates in a shift to the left of the center of mass of the overall genuine distribution. In this sense, increasing *f*_out_ increases the overlap of the genuine distribution with the spurious distribution and hence decreases the capacity of the network. At the same time, *f*_out_ directly impacts the placement of the activation threshold because we are always enforcing $M_{\text{out}}=f_{\text{out}}N_{\text{out}}$ active output units. This is discussed in detail in the Methods, Section ‘The activation threshold and balancing tails of the distributions’. The effect on the capacity of a change of the activation threshold due to an increase of *f*_out_ is unknown (also see Methods, Section ‘Deriving the Hamming distance from dendritic sums’). We speculate that this effect is small in the parameter range that we investigated numerically and that the effect of *f*_out_ via the weight distribution discussed above dominates the dependence of the capacity on *f*_out_.

### Role of network size and number of active units

So far, we have considered a fixed network size. We have seen how the capacity and the optimal transition probability depend on the input and the output activation ratio. Here, we discuss how these results depend on network sizes.

The following results are derived in detail in the Methods, Section ‘Analytical calculation of the capacity of the network’. Assuming that the average number of functional connections per output unit is large ($M_{\text{in}}c\gg 1$) but not too large ($M_{\text{in}}(1-\rho_{g}(\lfloor f_{\text{out}}(P+1)\rfloor ))\gg 1$) and $f_{\text{in}}\eta c_{m}/c\ll 1$, the capacity $P^{*}$ of the network can be analytically approximated as


$$\begin{array}{c} P^{*}=\frac{N_{\text{in}}c}{-\eta M_{\text{in}}c_{m}f_{\text{out}}}\cdot \ln\left(\frac{A(M_{\text{in}},c,f_{\text{out}},t_{S})}{B(M_{\text{in}},c_{m},c,f_{\text{out}},t_{S})\cdot \eta }\right) \end{array}$$ (21)

with *A* and *B* being two functions that are defined in Eqs (93) and (94) in the Methods section (see also Eq (98)). Note that both *A* and *B* are independent of the input and output network size *N*_in_ and *N*_out_. They only depend on the number of active input units *M*_in_ and the output activation ratio *f*_out_.

**Fig 5:**
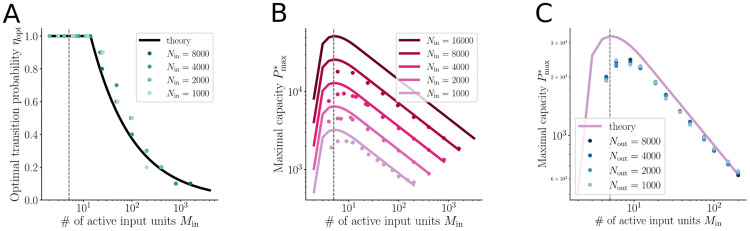
Role of network size and number of active units. Results from numerical simulations are depicted by dots, and theoretical results are depicted by solid lines. (**A**) The optimal transition probability $\eta_{\text{opt}}$ as a function of *M*_in_ monotonically decreases from a plateau at one to zero. $\eta_{\text{opt}}$ does not depend on the input layer size *N*_in_. The overall highest capacity for a fixed network size is obtained for an *M*_in_ which yields an optimal η of one (vertical gray dashed line). (**B**) shows the maximal capacity $P_{\text{max}}^{*}$ as a function of the number of active input units $M_{\text{in}}=f_{\text{in}}N_{\text{in}}$ for *N*_in_ = 1000, 2000, 4000, 8000 and 16000. The size of the output layer is fixed to *N*_out_ = 1000 in the simulations. The maximal capacity as a function of *M*_in_ scales linearly with *N*_in_. The vertical gray dashed line marks the number of active input units *M*_in_ that yields the highest capacity. The highest capacity for a fixed network size is obtained at the same *M*_in_ for any network size. In (**C**), the output layer size *N*_out_ (=1000, 2000, 4000, 8000) is varied instead of the input layer size *N*_in_. The number of input units *N*_in_ is fixed to 1000. The size of the output layer *N*_out_ does not have an impact on the maximal memory capacity $P_{\text{max}}^{*}$ of the network. Further parameter values in (**A**)–(**C**): $f_{\text{out}}=0.006,c=0.2,c_{m}=1,t_{S}=0.5,N_{\text{avg}}=200$.

Under the assumption $\frac{f_{\text{in}}\eta c_{m}}{c}\ll 1$, the optimal transition probability can be approximated by


$$\begin{array}{c} \eta_{opt}=\min\left(\frac{e\cdot A(M_{\text{in}},c,f_{\text{out}},t_{S})}{B(M_{\text{in}},c_{m},c,f_{\text{out}},t_{S})},1\right) \end{array}$$ (22)

(black line in Fig 5A, derived in Methods, Section ‘Optimal transition probability and maximal capacity’, see Eq (103)). It is independent of the input layer size *N*_in_ and the output layer size *N*_out_. The maximal capacity $P_{\text{max}}^{*}$ for a fixed set of network parameters is obtained by using the optimal transition probability $\eta_{\text{opt}}$ in Eq (21):


$$\begin{array}{c} P_{\text{max}}^{*}=P^{*}{|}_{\eta =\eta_{\text{opt}}}. \end{array}$$ (23)

We find analytically and numerically that the size of the input layer strongly impacts the maximal capacity of the network (see Fig 5B): The capacity $P^{*}$ scales linearly with the input layer size *N*_in_. Since $\eta_{\text{opt}}$ is independent of *N*_in_ (5A), the maximal capacity $P_{\text{max}}^{*}$ also scales linearly with *N*_in_ (Eq (21) and Fig 5B). The maximal capacity $P_{\text{max}}^{*}$ depends non-monotonically on the number of active input units *M*_in_. We find that, for any sufficiently large input layer size, the highest capacity is reached for the same number of active input units *M*_in_ (Fig 5B, gray line). According to Eqs (21) and (23), the maximum over *M*_in_ of the maximal capacity $P_{\text{max}}^{*}$ depends on the output activation ratio *f*_out_, the functional and morphological connectivities *c* and $c_{m}$ and of course, via $t_{S}$, also on the retrieval threshold $T_{S}=t_{S}H_{\text{avg}}$. Since, for a fixed network size *N*_in_, the input activation ratio $f_{\text{in}}=M_{\text{in}}/N_{\text{in}}$ is simply a scaled version of *M*_in_, the maximal capacity $P_{\text{max}}^{*}$ also depends non-monotonically on *f*_in_. We have seen examples of this non-monotonic dependence on *f*_in_ in Fig 3D.

In most figures, we show results for a morphological connectivity $c_{m}=1$ and a retrieval threshold $T_{S}=0.5H_{\text{avg}}$. However, our theory also holds for smaller, more realistic morphological connectivities of a few percent and more stringent retrieval criteria, as well as for a combination of the two. The main dependencies between activation ratios, optimal transition probability, and capacity are qualitatively robust to such changes. An increase of the retrieval threshold $T_{S}$ yields a larger optimal transition probability and, of course, a smaller capacity (S1 Fig). If the morphological connectivity $c_{m}$ decreases, $\eta_{\text{opt}}$ increases, while $P_{\text{max}}^{*}$ decreases (S2 Fig). The minimal number of active input units *M*_in_ that is necessary to store patterns and achieve non-zero capacity increases with a decrease in morphological connectivity $c_{m}$. An increase in functional connectivity *c* increases both $\eta_{\text{opt}}$ and $P_{\text{max}}^{*}$, at least for sufficiently large *M*_in_ (S3 Fig).

As a probability, the optimal transition probability is bounded between zero and one. It is a monotonically decreasing function of *M*_in_. For small values of *M*_in_, it takes the maximal value one. When *M*_in_ becomes large enough, the expression eA/B becomes smaller than one and $\eta_{\text{opt}}$ becomes strictly decreasing as a function of *M*_in_ (Fig 5A). This monotonicity of $\eta_{\text{opt}}$ as a function of *M*_in_ could not be formally shown with our theory but has been confirmed in all evaluations of Eq (22) and all numerical simulations that were performed. In the range where $\eta_{\text{opt}}<1$, the maximal capacity $P_{\text{max}}^{*}$ is monotonically decreasing as a function of *M*_in_ (shown in the Methods, Section ‘Dependence on input parameters’) (compare Fig 5A and 5B). It follows that, if the maximal capacity has a maximum as a function of *M*_in_ (for any given network size), it is reached within the range where we have $\eta_{\text{opt}}=1$ (Fig 5A, the gray line marks the *M*_in_ that yields the largest capacity). Note that, depending on the network parameters and the retrieval criterion, it can occur that there is no $M_{\text{in}}\geq 1$ for which $\eta_{\text{opt}}=1$. In this case, the dependence of the maximal capacity on *M*_in_ (as well as *f*_in_) is monotonic and the largest capacity is obtained for *M*_in_ = 1. The special role of the number of active input units for the optimal transition probability as well as for the maximal capacity is remarkable and we speculate that it is related to the fact that we choose the number *cN*_in_ of functional connections per output neuron to scale with the input network size.

As can be seen from Eqs (21) and (23) and as discussed in previous sections, the capacity depends on the output activation ratio *f*_out_. For adequately large *N*_out_, the size of the output layer, on the other hand, does not affect the storage capacity of the network (Eq (21) and Fig 5C, also see Discussion, Section ‘Role of the output size and retrieval criterion’).

### Noisy input patterns during retrieval

**Fig 6:**
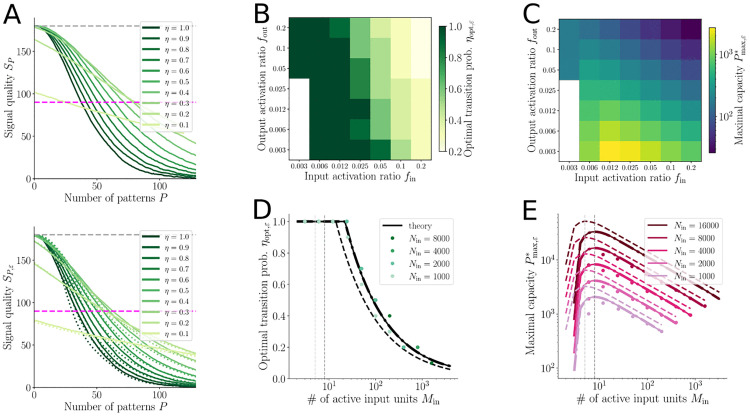
With noisy input patterns during retrieval, maximal capacity decreases and optimal transition probability increases. The noise level is set to ε=0.2 in the entire figure. (**A**) The memory signal quality $S_{P}$ decays with the number *P* of patterns learned by the network. For smaller transition probabilities η, the signal quality $S_{P}$ starts lower but decays more slowly than for larger η. Top: No noise during retrieval; the largest capacity is attained for η=0.2. Bottom: With noise on the input patterns during retrieval; the signal quality $S_{P,\varepsilon }$ is lower than without noise, particularly for small transition probabilities η. All capacity values with noise are slightly smaller than the corresponding ones without noise. The largest capacity is now attained for η=0.3. In (**A**) the solid curves show results from simulations. The dotted curves are an approximation of the signal quality with noise $S_{P,\varepsilon }$ where the signal quality without noise $S_{P}$ is multiplied by a factor obtained from theory (see S3 Appendix). Fixed parameters in (**A**): $f_{\text{in}}=f_{\text{out}}=0.1$. (**B**),(**C**) The optimal transition probability $\eta_{\text{opt},\varepsilon }$ and the maximal memory capacity $P_{\text{max},\varepsilon }^{*}$ obtained with noise during retrieval as functions of input and output activation ratios *f*_in_ and *f*_out_. $\eta_{\text{opt},\varepsilon }$ and $P_{\text{max},\varepsilon }^{*}$ behave qualitatively similar to the maximal memory capacity $P_{\text{max}}^{*}$ and the optimal transition probability $\eta_{\text{opt}}$ obtained without noise (cf. Fig 3D,3E). In (**A**)–(**C**): $N_{\text{in}}=N_{\text{out}}=1000$. (**D**) The optimal transition probability $\eta_{\text{opt},\varepsilon }$ with noise (solid curve, Eq (24)) is slightly higher than without noise (dashed curve, Eq (22)). The dash-dotted curve (which aligns closely with the solid curve) shows an approximation of $\eta_{\text{opt},\varepsilon }$ as a multiple of $\eta_{\text{opt}}$ (Eq (26)). The vertical dark gray and light gray dashed lines mark the *M*_in_ that yields the highest capacity with and without noise, respectively. With noise, the optimal *M*_in_ is larger than without noise. All black curves are derived from theoretical considerations, the colored dots show results from simulations (*N*_out_ fixed to 1000). (**E**) Noise on the input patterns during retrieval reduces the maximal capacity $P_{\text{max},\varepsilon }^{*}$. Furthermore, $P_{\text{max},\varepsilon }^{*}$ scales with the input layer size *N*_in_. Dashed curves show the maximal capacity without noise (Eq (23)), solid curves show the maximal capacity with noise (Eq (27)), dash-dotted curves (which align closely with the solid curves) show an approximation of the maximal capacity with noise for small ε (Eq (28)), and dots show numerical results (*N*_out_ fixed to 1000). Further parameter values in (**A**)–(**E**): $c=0.2,c_{m}=1,t_{S}=0.5,N_{\text{avg}}=200$.

We previously (Fig 3A) found that the transition probability that leads to the largest capacity depends on a balance between a large initial signal quality and a slow decay of the signal quality with the number of subsequently stored patterns. The top panel of Fig 6A also shows the signal quality as a function of the number of patterns but for a different set of input and output parameters ($f_{\text{in}}=f_{\text{out}}=0.1$) than Fig 3A. Let us now investigate the effect of noisy input patterns during retrieval on the optimal transition probability and the maximal capacity of the network.

The input pattern that is presented to the network during the retrieval phase does not necessarily have to be the same pattern that was used for training. From a biological perspective, it makes sense to assume that the input during retrieval is not exactly the same but a noisy version of the original input pattern. We follow a similar approach as [46,47]. The noisy input patterns are created by deactivating a particular fraction ε of the active input units while activating the same number of inactive input units. The input activation ratio *f*_in_ is hence maintained. Adding noise to the input pattern during retrieval reduces the memory capacity by deteriorating the signal quality right from the beginning (Fig 6A, top vs bottom). This shift in the initial signal quality is particularly strong for small transition probabilities η because the memory relies on few connections to provide a strong input to the output units. If some of the input units that are supposed to be active in a pattern are inactive and others are activated instead, it is likely that many of the genuine output units are not activated anymore. The effect is less drastic for large transition probabilities for which the memory is more reliably imprinted into many connections and does not get lost just because some input units are wrongly activated. However, we find that the change in the signal quality due to noise can neither be explained by a constant shift nor by a constant multiplicative scaling. The change depends on the number of patterns in a more complex way, see S3 Appendix. There we derived an analytical description of the factor that has to be applied to the signal quality $S_{P}$ calculated without noise during retrieval to obtain an estimate of the signal quality $S_{P,\varepsilon }$ with noise.

Despite the complex relationship between $S_{P}$ and $S_{P,\varepsilon }$, the qualitative dependence of the optimal transition probability $\eta_{\text{opt},\varepsilon }$ on the input and output activation ratios remains the same as without noise (Fig 6B, compare to Fig 3E) — it increases with decreasing activation ratios. Further, adding noise to the input pattern during retrieval maintains the non-monotonic effect of the input activation ratio and the monotonic effect of the output activation ratio on the maximal capacity (Fig 6C, compare to Fig 3D).

We extend our theory to retrieval with noisy input patterns and find that the optimal transition probability can again be described as


$$\begin{array}{c} \eta_{\text{opt},\varepsilon }=\min\left(\frac{e\cdot A_{\varepsilon }(M_{\text{in}},c,f_{\text{out}},t_{S},\varepsilon )}{B_{\varepsilon }(M_{\text{in}},c_{m},c,f_{\text{out}},t_{S},\varepsilon )},1\right), \end{array}$$ (24)

where $A_{\varepsilon }$ and $B_{\varepsilon }$ (Eqs (112) and (113)) now additionally depend on the noise level ε (derived in Methods, Section ‘Extension to noisy input patterns during retrieval’). For a fixed set of parameters, noise during retrieval increases the optimal transition probability:


$$\begin{array}{c} \eta_{\text{opt},\varepsilon }\geq \eta_{\text{opt}} \end{array}$$ (25)

(Fig 6D, compare solid line to dashed line). It can be approximated as a scaled version of the optimal transition probability without noise:


$$\begin{array}{c} \eta_{\text{opt},\varepsilon }\approx \frac{\eta_{\text{opt}}}{1-\frac{\varepsilon }{1-f_{\text{in}}}} \end{array}$$ (26)

(Fig 6D, dash-dotted lines, also see S4 Fig). As discussed previously, the optimal transition probability corresponds to a sweet spot that realizes both a good initial encoding of the memory and a slow decay of the memory trace. The additional noise impacts both aspects but since we find that $\eta_{\text{opt},\varepsilon }\geq \eta_{\text{opt}}$, the noise has a more detrimental effect on the initial signal quality than on the decay of the signal quality.

The maximal capacity can again be theoretically described as


$$\begin{array}{c} P_{\text{max},\varepsilon }^{*}=\frac{N_{\text{in}}c}{-\eta_{\text{opt},\varepsilon }M_{\text{in}}c_{m}f_{\text{out}}}\cdot \ln\left(\frac{A_{\varepsilon }(M_{\text{in}},c,f_{\text{out}},t_{S},\varepsilon )}{B_{\varepsilon }(M_{\text{in}},c_{m},c,f_{\text{out}},t_{S},\varepsilon )\cdot \eta_{\text{opt},\varepsilon }}\right), \end{array}$$ (27)

(Fig 6E, solid lines, derived in Methods, Section ‘Extension to noisy input patterns during retrieval’, see also S4 Fig). It can further be approximated by


$$\begin{array}{c} P_{\text{max},\varepsilon }^{*}\approx P^{*}{|}_{\eta =\eta_{\text{opt},\varepsilon }}+\frac{\ln\left(1-\frac{\varepsilon }{1-f_{\text{in}}}\right)c}{f_{\text{in}}f_{\text{out}}\eta_{\text{opt},\varepsilon }c_{m}}\approx P^{*}{|}_{\eta =\eta_{\text{opt},\varepsilon }}-\frac{\varepsilon c}{f_{\text{in}}f_{\text{out}}\eta_{\text{opt},\varepsilon }c_{m}}, \end{array}$$ (28)

where $P^{*}{|}_{\eta =\eta_{\text{opt},\varepsilon }}$ is the capacity without noise (Eq (21)) evaluated for $\eta =\eta_{\text{opt},\varepsilon }$ and the last approximation step assumes ε≪1 and $f_{\text{in}}\ll 1$ (Fig 6E, dash-dotted lines, see also S4 Fig; derived in S4 Appendix). Hence, the maximal capacity with noise during retrieval is the maximal capacity without noise (evaluated at $\eta_{\text{opt},\varepsilon }$) shifted down by a fixed amount that scales linearly with ε. The *M*_in_ that yields the highest capacity is again independent of *N*_in_ but it increases with the noise level ε (Fig 6E, vertical dashed lines, see also S4 Fig).

We can conclude that in a system with noisy input patterns, the capacity is lower than in a system without noise. However, with noise, it is beneficial for the capacity to learn with a higher transition probability.

## Discussion

We studied the effect of population sparseness on the optimal transition probability of synaptic weights for maximum memory capacity in an associative network. We quantified this storage capacity in a heteroassociative feedforward network, where the capacity is based on a threshold of the Hamming distance between the target patterns and the calculated output patterns, as illustrated in Figs 1 and 2. Furthermore, we determined the transition probability that maximizes the capacity (see Eqs (21)–(23)). Analytical derivations and numerical simulations show that this optimal transition probability increases with the sparseness of both the input and the output patterns, but the impact of the input sparseness is stronger (Fig 3B,3C,3E). The maximum capacity is largest for the lowest *output* activation ratio, whereas it depends non-monotonically on the *input* activation ratio (Fig 3B,3C,3D). These results are accompanied by a theoretical analysis based on the distributions of the dendritic sums that provides an intuitive understanding of the mentioned dependencies (Fig 4). Moreover, the maximum capacity is extensive with respect to the input layer size, but the optimal transition probability is independent of the network size (Fig 5). The crucial parameter that determines the optimal transition probability is the actual number of active input units and not the input activation ratio (Fig 5A). The number of active input units that yields the largest possible capacity is also independent of the network size (Fig 5B,5C). All results hold for recall with perfect input patterns as well as with noisy input patterns. As expected, the maximal capacity decreases as a function of the noise level but, interestingly, the optimal transition probability increases (Fig 6). Together, our in-depth numerical and analytical analysis of memory capacity based on Hebbian and homeostatic learning presents a substantial advancement, as it reveals the distinct effects of input and output sparseness as well as network size and input noise on the optimal strength of plasticity while relying on a very simple network and pattern structure.

From a methodological perspective, the theoretical derivations in this manuscript build on previous work on the capacity of binary networks (see, e.g., [43,44,47,48,52]) that was derived via the distributions of dendritic sums (see, e.g., [45–47,49–51]). These earlier approaches use setups that have some similarities with the one analyzed here — like sparse patterns [46,47,49], clipped Hebbian learning [46,49], or imperfect retrieval cues [46,47]. However, the homeostatic effect of a predefined in-degree and of a fixed output sparseness, which complicate the analytical description of the network capacity, were not considered before. In other regards, the present work has a different focus than previous work, which, for example, investigated the specific statistical properties of distributions of dendritic sums [50,51,61], compared different thresholding strategies [45,46], or used a bidirectional memory structure [49]. The new analytical contributions of the present work can thus be summarized as follows: Distributions of dendritic sums are derived for a learning rule where the number of functional connections per output neuron is fixed. To our knowledge, the effect of such a homeostasis rule on the behavior of the distributions of dendritic sums has not been analyzed in detail before. Furthermore, the memory capacity is derived for an activation threshold that is chosen self-consistently so that the output activation ratio is maintained. This introduces a *Balance Equation* (Eq (15)) that needs to be fulfilled by a certain activation threshold. Moreover, and most importantly, the current work includes a parameter that describes the strength of plasticity of the network and, therefore, allows for an analytical investigation of how the input and output activation ratios impact the optimal strength of plasticity and the maximal capacity.

### Palimpsest memory and homeostatic plasticity

The architecture of the network used in this manuscript is similar to the Willshaw network [29] in that it is a feedforward one-layer network that learns associations between binary input and output patterns in a Hebbian way (see Section ‘Network model, learning paradigm, and quantification of capacity’). In contrast to a classical Willshaw network in which all memories disappear when the capacity is reached, our network realizes a palimpsest memory system in which older memories are erased by newer memories due to a homeostasis mechanism (see, e.g., Fig 3A). This degradation of older memories allows for an online learning regime where an in principle infinite sequence of patterns can be learned — new patterns always gradually replacing older ones [36,38]. The signal quality of a memory pattern hence does not only passively decay over time due to the interference between patterns, but also decays actively due to the deactivation or depression of synaptic connections. The underlying heterosynaptic plasticity, which is characterized by an input-unspecific change of synaptic efficacies [62,63], has been found, e.g., in the hippocampus and the neocortex [64,65]. Heterosynaptic plasticity can provide an effective homeostatic constraint on Hebbian plasticity [66,67]. In the presented work, a combination of Hebbian homosynaptic and homeostatic heterosynaptic learning was used (see Section ‘Network model, learning paradigm, and quantification of capacity’). Hebbian plasticity increases connection weights with a predefined probability if both the pre- and the postsynaptic units are active, and homeostatic plasticity decreases weights with another predefined probability if only the postsynaptic unit is active (details in Methods, Section ‘Hebbian and homeostatic update rules from a probabilistic perspective’). The update probabilities, which represent the strength of plasticity, are balanced such that the total number of functional connections per output unit remains constant. This special rule of heterosynaptic plasticity is inspired by experimental evidence of a constant total summed synaptic weight on a dendritic branch [54,68].

In general, variations in the implemented homeostatic plasticity are possible. For example, in contrast to our approach, the total number of functional connections per input unit could be conserved to achieve presynaptic homeostasis by depressing connections from active input neurons to inactive output neurons (as suggested by [36], e.g., reported by [69]). Alternatively, the number of functional connections could be conserved only throughout the network, i.e., not normalized per input or per output unit; such a normalization could be achieved by randomly silencing connections that are not involved in a given association of patterns [37]. Numerical simulations based on either of these two variations suggest that the qualitative dependence of the optimal strength of plasticity on the sparseness of the patterns is the same as for the learning rule analyzed in detail in this paper (see S6 Appendix). Finally, a change in synaptic efficacies could occur due to synaptic aging where connections that have not been involved in a pre- and postsynaptic co-activity for a long time might be more likely to be depressed than those that have been strengthened more recently [70]. Such an age-dependent homeostasis rule requires synapses with metaplasticity, which could have a beneficial effect on the storage capacity of the network [38,40,71] even though not necessarily for sparse codes [38]. Furthermore, models that incorporate structural plasticity focus not only on changes in connection strength but also on the formation and elimination of synapses and the remodeling of dendritic spines [72,73]. Such models have been shown to be capable of increasing storage capacity, in particular for sparse patterns in sparsely connected networks [74,75]. It is beyond the scope of this manuscript to analyze in detail the impact of metaplasticity or structural plasticity.

### Input sparseness vs output sparseness and morphological connectivity

In our heteroassociative network, both a change in the input and the output sparseness value affect the optimal strength of plasticity and the corresponding maximum memory capacity (Figs 3, 5, and 6, Eqs (21)–23)). The optimal transition probability monotonically decreases with an increase in the input activation ratio as well as with an increase in the output activation ratio, but the gradient is steeper for the input activation ratio (see Figs 3E, 6B, 13). In order to learn with high plasticity, sparseness in the input representations is thus more beneficial than sparseness in the output representations.

As known from the literature [29,33,34,36], the capacity of an associative network benefits from sparse representations. We find that the same is true for the maximum capacity, which is obtained by learning with the optimal transition probability for each combination of parameters. While enforcing sparser output patterns decreases the overlap in the output space and hence decreases the likelihood of retrieval errors up to extreme sparseness values of only one active output unit, using too sparse input patterns can be detrimental to the capacity. If the input representations contain a too small number of active units *M*_in_, it is difficult for the signal, which scales as *M*_in_, to overcome the noise, which scales as $\sqrt{M_{\text{in}}}$ (Fig 3A,3B and Fig 5B). In other words, a too small number of active input units cannot reliably drive the respective output units. If *M*_in_ is smaller than some optimal value, the initial signal quality approaches the retrieval threshold and the capacity decreases, and if *M*_in_ becomes even smaller, the initial signal quality can be below the retrieval threshold and therefore the capacity is zero [37,38,46,52]. This collapse of the maximal capacity for too sparse input patterns occurs only in the range of *M*_in_-values where the optimal transition probability has already reached its maximal value 1 (Fig 5A; details in Methods, Section ‘Dependence on input parameters’). Therefore, for too sparse input patterns, a decrease in *M*_in_ cannot be compensated by an increase in the transition probability.

A similar limitation may arise in networks with strongly diluted morphological connectivity $c_{m}\ll 1$. Such connectivity values are substantially more biologically realistic than the fully connected case $c_{m}=1$ (used in many figures of this manuscript), since anatomical connectivity in cortical networks is typically sparse [76]. In terms of the approximation of the distributions of dendritic sums by a binomial distribution, $c_{m}=1$ is the worst-case scenario (see S4 Appendix for details). The analytical approximation of the capacity derived in this manuscript hence improves significantly for more realistic values of $c_{m}<1$. However, in the case $c_{m}\ll 1$, particularly for very sparse input representations, the number of active feedforward inputs contributing to the signal can become too small for reliable retrieval. As discussed in previous work on sparse associative memories [74,75], very sparse morphological connectivity can therefore further reduce the robustness of sparse representations and lead to vanishing capacities. To address this point, we evaluated the model for a wide range of small morphological connectivity values $c_{m}<1$. As expected, reduced connectivity decreases the achievable capacity and increases the optimal transition probability to compensate for the reduced number of available synapses. However, even for very small morphological connectivities $c_{m}\ll 1$ and strict retrieval criteria ($t_{S}$ close to 1), many patterns can be learned and successfully retrieved if the number of active input units *M*_in_ is sufficiently large — and the qualitative dependencies of the capacity and strength of plasticity on sparseness are maintained (S1 Fig and S2 Fig).

### Choice of the activation threshold and optimized capacity

The central purpose of this work is to establish a relationship between the population sparseness of the patterns and the optimal strength of plasticity of the network to maximize its capacity. This requires quantifying the capacity, i.e., the lifetime of a pattern; therefore, the setup must allow for a meaningful comparison of the target and calculated output patterns. As a measure, we used the Hamming distance between the target and the calculated output patterns. This measure is most robust if the number of active output units *M*_out_ is fixed, which is implemented by choosing the activation threshold of the McCulloch-Pitts output neurons accordingly (Eq (3)). The maximum capacity calculated in this framework (Eq (23)) represents the highest capacity obtained under the given constraints using the optimal transition probability. In other approaches, the beneficial effect of sparseness on capacity has been extensively studied in different settings. For example, [34] could increase the capacity by using an optimized covariance-based learning rule. Moreover, [46] found that the capacity can be optimized by individually adjusting the threshold of each neuron, and [77] suggested the use of a numerically optimized activation threshold in a setup that is very similar to ours. A general model that unifies earlier Willshaw- and Hopfield-type networks provides optimal synaptic weights as well as an optimal firing threshold for each neuron [78]. In our manuscript, such aspects have been excluded in an attempt to keep the model as simple as possible to allow for a detailed analysis of the effect of sparseness on the strength of plasticity.

### Capacity measures

A limitation of the present work is the use of the performance measure “pattern capacity”, which is defined as the number of subsequent associations that can be stored before losing retrievability of a pattern. As an alternative, network capacity is commonly also defined through information-theoretic measures that optimize resource use, such as synaptic capacity (see, e.g., [29,43,52,57]). For sparse patterns, it has been shown that parameters that yield optimal pattern capacity do not necessarily also optimize synaptic (information) capacity [47,52,57], implying an under-utilization of synaptic resources. In addition, outside the very sparse regime, when the number of active neurons per pattern becomes sufficiently large, optimizing pattern capacity may in principle overestimate the amount of synaptic memory required to store a given number of associations. In this case, the information content of the stored sparse patterns exceeds the minimal information necessary to distinguish between *P* memories, and alternative memory models could therefore encode lighter-weight representations using fewer synaptic resources. In our work, pattern capacity is employed as a functional measure of memory performance, focusing on the ability of the network to reliably retrieve stored patterns. It is not intended to quantify the information-theoretic optimality of synaptic storage. The results are thus best understood as characterizing the retrieval performance for different activation ratios and transition probabilities under the given homeostatic constraints of the network—and not as statements about how efficiently synapses are used in terms of information storage.

In S8 Appendix, we provide a translation of our main results into the information-theoretic measure of synaptic capacity. These preliminary results suggest that the decrease of the optimal transition probability with increasing activation ratios, which is the core result of the present work, is maintained for synaptic capacity. The synaptic capacity obtained with the optimal transition probability decreases with increasing input and output activation ratios. A detailed information-theoretic analysis of these results is beyond the scope of this study, but provides an interesting direction for future work.

### Role of the output size and retrieval criterion

The analytical description of memory capacity relies on the distributions of dendritic sums, which are based on the synaptic connections of single output units (Eqs (10) and (11)). For the Hamming distance (Eq (14)) and thus also for the signal quality $S_{P}$ (Eq (7), also see Methods, Section ‘Signal quality after *P* subsequent patterns’), the number of output units *N*_out_ has to be taken into account because the probabilities of output units being in the wrong state are scaled by the respective number of units. We defined memory capacity as the number of subsequent patterns that can be learned until the signal quality $S_{P}$ reaches the retrieval threshold $T_{S}$ (Eq (8) and Eq (17)). The retrieval threshold $T_{S}=t_{S}H_{\text{avg}}$, with a fixed $t_{S}\in (0,1)$, is chosen as a fraction of $H_{\text{avg}}=2N_{\text{out}}f_{\text{out}}(1-f_{\text{out}})$, which is the average Hamming distance between two random *f*_out_-sparse patterns of size *N*_out_ (Eq (9)). For most figures in this manuscript, we chose $t_{S}=0.5$ as an exemplary retrieval ratio, where up to half of the units of a pattern are allowed to be wrong. However, network simulations as well as the analytical theory show that the main results, such as the monotonic decrease of the optimal transition probability as a function of the activation ratios and the non-monotonic dependence of the maximal capacity on the number of active input units, are robust with respect to changes in the retrieval threshold (see S1 Fig). The qualitative results hold also for much less permissive retrieval criteria with high values of $t_{S}$ that allow only for a small percentage of wrong units.

Due to the choice of the retrieval threshold $T_{S}$ as a multiple of *H*_avg_, the number of wrong output units that are accepted for the pattern to be classified as retrievable depends on *N*_out_ and *f*_out_. This dependence accounts for the fact that a fixed number of wrongly activated units has a more severe destructive effect if the total number of active output units is small. For example, if in a pattern with *M*_out_ = 100 active output units nine are wrong, the pattern is still very similar to the original one; if *M*_out_ = 10, nine wrong units are detrimental. The capacity is obtained by comparing the signal quality $S_{P}$ to the retrieval threshold $T_{S}$ or, equivalently, by comparing the normalized signal quality $S_{P}/H_{\text{avg}}$ to the retrieval ratio $t_{S}$. Since the normalized signal quality $S_{P}/H_{\text{avg}}$ does not depend on *N*_out_ (derived in Methods, Section ‘Signal quality after *P* subsequent patterns’) but only on the distributions of the dendritic sums and *f*_out_ (see Eqs (59) and (61) as well as S3 Appendix), the capacity does not depend on *N*_out_ either.

As an alternative, which has not been explored in this manuscript, the retrieval threshold could be defined independently of *H*_avg_ and thus independently of *N*_out_, e.g., implementing the retrieval criterion that a maximum of two units may be wrongly activated or that at least ten units must be correctly activated. In such alternative cases, the capacity could depend on the output network size *N*_out_.

Furthermore, the output size as well as the input size in combination with the output and input activation ratios, respectively, pose an upper limit to the representational capacity, i.e., the maximal number of distinct patterns. If every input pattern and every output pattern should occur maximally once, the number of distinct input-output pattern pairs is bounded by min((NinMin),(NoutMout)).

In general, however, even if the size of the output layer is negligible for the storage capacity under specific circumstances, it plays an important role in a potential additional readout layer, e.g., a classification of the output representations. In [79], it has been shown that an increase in the number of output units increases the performance of a readout unit that performs a binary classification.

### Strength of plasticity and related concepts

In the context of strength of plasticity, several distinct but related notions are used. Here, it seems useful to explain and compare the concepts of transition probability, weight step size, and learning rate, which could all be used to model the strength of synaptic plasticity. Apart from that, we make the distinction between the strength of plasticity and the speed of learning. The discussion of the speed of learning introduces the additional notions of number of trials and convergence time.

In this manuscript, we model the strength of plasticity by the transition probability, which refers to the likelihood that a synaptic weight increases during learning. Instead, a change in the strength of plasticity could also be realized by a change in the magnitude of synaptic weight updates (here called the *weight step size*). Weights could, for instance, be modeled to take on a number of discrete values. A high degree of plasticity could then be realized by a larger jump from a small weight value to a large weight value, while a low degree of plasticity would allow for updates from a small to an intermediate weight value. The transition probability and the weight step size are closely related as both affect changes in weights, and the average weight change in one learning step is proportional to the product of the two parameters. The qualitative results for the effect of sparseness on the optimal strength of plasticity of the present study are likely to also hold for models based on the weight step size or on a combination of both the transition probability and the weight step size. Note that while the assumption of only binary synaptic weights combined with a transition probability is a simplification of the state transitions that occur in real synapses, assuming a bounded number of discrete states is more realistic [80,81]. It therefore makes sense to assume that the strength of plasticity — e.g., in terms of the amount of potentiation experienced by synapses combined with a probability of potentiation — is bound to have an upper limit.

In this manuscript, we consider a time-discrete model of plasticity. In contrast, in time-continuous models, the strength of plasticity could be represented by the *learning rate*, which controls how fast the weights change. Again, we believe that a time-continuous model would yield qualitatively similar results to our time-discrete model relying on the transition probability if the number of connections in the network is large. It would generally be interesting to analyze more complex and more realistic synapse models (e.g., [38,40,71]) in this context, but all this is beyond the scope of the present work.

Furthermore, the strength of plasticity should be distinguished from the speed of learning. In our learning paradigm, the number of learning trials is kept constant and equal to one. Thus, each pattern pair is learned in one shot by changing a number of connections, which depends on the strength of plasticity. Although high plasticity and more rapid learning can often go hand in hand (also see next section ‘Sparseness and plasticity in the brain’), the *speed of learning* (one trial) is here fixed and thus not a parameter of investigation in this study.

Finally, we relate the so-called “convergence time” to the strength of plasticity, although the term “convergence” and its magnitude do not refer to how many or how much weights are altered, but it is a system-level outcome. Convergence refers to how quickly a neural network stabilizes or reaches its learning objective, e.g., a minimum in a gradient-descent learning algorithm. While very plastic synapses (e.g., in terms of transition probability, weight step size, or learning rate) can speed up appropriate weight updates, fast convergence is achieved by balancing these factors to ensure that the network efficiently settles into an optimal or stable solution. A high learning rate can accelerate updates towards a solution, but it does not necessarily relate to fast convergence. If the update steps are too large, they risk overshooting or instability. In [82] it is argued that sparse representations yield faster convergence because the learning rate can be increased without risking instabilities, but how this relates to the capacity of the network was not investigated. Furthermore, it is not clear how this translates to biologically more plausible local learning paradigms. In our model, convergence is not evaluated because every pattern is presented only once, and thus there is only one weight update step. It would be interesting to investigate the effect of sparseness on transition probability in combination with convergence speed in a network model that allows for both. Building on the results of this manuscript and the reasoning in [82], we believe that high sparseness would yield a high optimal transition probability (or learning rate) while converging fast because sparseness allows for large update steps without instability. A concrete possibility of introducing the concept of convergence into our model — when allowing repeated encoding of a pattern pair — is to analyze how many trials are necessary to perfectly imprint, in the sense that all connections from active input to active output units in the pattern are functional, the association into the network. For a transition probability of one, this is the case after one shot. For lower transition probabilities, it could take many more trials, i.e., the speed of learning would be slower.

### Sparseness and plasticity in the brain

As already outlined in the Introduction, high population sparseness is prevalent throughout the brain [1–3,5,6,8,9,20]. Our work suggests that brain areas with higher sparseness could make use of more plastic synapses than brain areas with lower sparseness. The results of several experimental studies support this relation between increased sparseness and higher plasticity, which often goes together with more rapid learning.

The hippocampus, for example, is well known for its highly sparse representations and is also well known to be involved in rapid learning with high plasticity of synapses, serving episodic and spatial memory. A high turnover rate of dendritic spines as an indicator of high plasticity has been reported for the mouse hippocampus [83]. Studies using single-trial learning paradigms showed that the hippocampus can encode and store information after only a single exposure to an experience [84,85]. This is often called one-shot learning. Research with rodents [86–88] and humans [89–92] demonstrates that concept cells can develop very quickly (within seconds to minutes) and that hippocampal neurons can rapidly form and reorganize place fields or episodic representations during single learning episodes.

Compared to the hippocampus, the neocortex seems to have a lower population sparseness. According to the theory of complementary learning systems in the context of systems memory consolidation, the neocortex is assumed to be a slow learning system, which needs repetitive exposure to acquire a new memory, for example, in the form of repeated memory replay by the hippocampus during slow-wave sleep [93–95]. This feature is in line with the observation that plasticity in the neocortex is lower than in the hippocampus [96,97]. An artificial increase in plasticity in the neocortex has been reported to disrupt memory processes [98]. However, engrams can also form rapidly in the neocortex, either in parallel with engrams in the hippocampus or even without hippocampal support [84,99,100]. Fast memory formation was observed in the neocortex in rodents, for example, in combination with unusually high plasticity in neocortical regions for memories related to prior knowledge [101,102] and in humans during the learning of new words [103]. A recent analysis of a large set of fMRI data suggests that under specific conditions the human parahippocampal cortex (PHC), a part of the neocortex, can exhibit even higher plasticity than the hippocampus [104].

In light of these studies and our theoretical considerations, a deeper experimental investigation of the causal or functional relationship between sparseness and the strength of plasticity would be of great interest for the neuroscience community.

### Optimal sparseness values

In terms of the capacity obtained with an optimized transition probability, our study predicts an optimal small but not too small input activation ratio *f*_in_ and an optimal output activation ratio $f_{\text{out}}=1/N_{\text{out}}$ (Fig 3). The optimal *f*_in_ depends on the input size *N*_in_ while the optimal number of active input units *M*_in_ does not (Fig 5). Nevertheless, the optimal *M*_in_ cannot be interpreted as a quantitative prediction of the optimal number of active units because it depends on the retrieval threshold $T_{S}$ (see Methods, Section ‘Dependence on input parameters’). If $T_{S}$ is increased, i.e., if the retrieval criterion becomes more strict, the optimal *M*_in_ increases (and the corresponding maximum capacity decreases).

In addition, the quantification of the optimal strength of plasticity in our model is focused solely on optimizing the pattern capacity of the network. In reality, the brain might favor a trade-off of various properties instead of optimization for one purpose [105]. Although sparseness can be advantageous, e.g., in terms of energy efficiency [106], storage capacity [107], and downstream classification [79], it might be beneficial to avoid extreme sparseness because it can reduce the representational capacity [105,108], the information capacity per pattern [109,110], the generalization capacity [105,111]; and extreme sparseness is also not robust to noise or damage of neuronal structures [108, 105]. Moreover, sparse connectivity, which is also ubiquitous in the brain, seems to yield similar favorable effects as population sparseness and could be optimized in combination with sparse activity [105,112].

In general, it is therefore not reasonable to predict *one* optimal activation ratio or *one* optimal number of active neurons. Optimal (and actual) values are probably different for different brain regions, cell types, tasks, etc. First steps towards more concrete optimal sparseness values could combine experimentally measured values, obtained with modern sophisticated techniques such as Neuropixels recordings, and computational models that take into account the specific properties and presumed role(s) of particular brain regions.

## Methods

In the following, we give a detailed account of the analytical derivations and numerical implementations used to obtain the results presented in the ‘Results’. In the first part of the Methods (section ‘Probabilistic description of dendritic sums’), we derive the distributions of dendritic sums, which provides the basis for the subsequent derivation of analytical expressions for memory capacity in the second part of the Methods (section ‘Analytical calculation of the capacity of the network’). Finally, in the third part of the Methods (section ‘Network simulations’), we describe the numerical implementation of the algorithm. Table 1 provides a summary of the main parameters.

**Table 1:** Description of variable names and their default values.

Variable	Description	Default Value
*N*_in_	Number of input units	1000
*N*_out_	Number of output units	1000
*f*_in_	Input activation ratio	0.025
*f*_out_	Output activation ratio	0.025
*M*_in_	Number of active input units	25
*M*_out_	Number of active output units	25
*J* ^[*k*]^	Binary weight matrix after learning *k* patterns	
$c_{m}$	Morphological connectivity	1
*c*	Functional connectivity	0.2
η	Transition probability	
$\eta_{\text{opt}}$	Optimal transition probability	
${𝐱}^{\left[k\right]}$	*k*-th input pattern	
${𝐲}^{\left[k\right]}$	*k*-th output pattern	
${\hat{𝐲}}^{\left[k\right]}(l)$	Output given the *k*-th input pattern calculated after learning *l* additional patterns	
*P*	Number of subsequent patterns learned since a particular pattern	
*K*	Total number of patterns learned at the end of a simulation	
*N*_avg_	Minimal number of patterns used for averaging in simulations	200
$T_{S}=t_{S}H_{\text{avg}}$	Retrieval threshold	
$t_{S}$	Retrieval ratio	0.5
*H*_avg_	Average Hamming distance between random patterns with given size and activation ratio	
*H*(*a*,*b*)	Hamming distance between *a* and *b*	
$S_{P}=s_{P}H_{\text{avg}}$	Signal quality after *P* subsequent patterns	
$s_{P}$	Normalized signal quality after *P* subsequent patterns	
$P^{*}$	Memory capacity	
$P_{\text{max}}^{*}$	Maximal memory capacity (with optimal transition probability)	
ε	Noise level on input patterns	0.2
$d_{g}^{\left[P\right]}$	Dendritic sum of genuine unit after *P* subsequent patterns	
$d_{s}$	Dendritic sum of spurious unit	
$p_{g}^{\left[P\right]}$	PMF of dendritic sums of genuine units after *P* subsequent patterns	
$p_{s}$	PMF of dendritic sums of spurious units	
$F_{g}^{\left[P\right]}$	CDF of dendritic sums of genuine units after *P* subsequent patterns	
$F_{s}$	CDF of dendritic sums of spurious units	
$\rho_{g}$	Probability of a functional genuine-genuine connection	
$\rho_{s}$	Probability of a functional genuine-spurious connection	
$\rho_{n}$	Probability of a functional spurious-genuine connection	
$d_{R}^{\prime }$	Generalized sensitivity index	

The three parts of the Methods can be read independently. They are written in a self-contained way so that they can be read independently of the Results section. Therefore, we first briefly recap the network model and the learning paradigm. The network is presented with a sequence of input/output-pattern pairs $\left({𝐱}^{\left[k\right]},{𝐲}^{\left[k\right]}\right)$, k∈{0,1,…}. Each pattern pair is learned in one shot. Input and output patterns are binary vectors of length *N*_in_ and *N*_out_, respectively, and of activation ratio *f*_in_ and *f*_out_, respectively. Hence, they consist of $M_{\text{in}}=f_{\text{in}}N_{\text{in}}$ or $M_{\text{out}}=f_{\text{out}}N_{\text{out}}$ active units and $N_{\text{in}}-M_{\text{in}}$ or $N_{\text{out}}-M_{\text{out}}$ inactive units, respectively.

The input and output layers of the network are connected by a weight matrix *J*. Per output unit, a number $c_{m}N_{\text{in}}$ connections ($c_{m}N_{\text{in}}$ entries per row of the weight matrix) are randomly chosen to be morphologically available connections. They can be functional (‘on’, value 1) or silent (‘off’, value 0). The functional connectivity *c* is always normalized such that a fixed number *cN*_in_ of morphologically available connections per output unit is functional.

In each learning step, the weight matrix is updated according to the presented pattern pair $\left({𝐱}^{\left[k\right]},{𝐲}^{\left[k\right]}\right)$. Silent connections between active input and active output units are turned on with a transition probability η. In addition, we randomly silence the same number of functional connections from inactive input units to active output units such that the total number of functional connections per output unit is maintained. This update step transforms $J^{\left[k-1\right]}$ into *J*^[*k*]^. The transition probability η represents the *strength of plasticity* (or *degree of plasticity*) of the network. While this plasticity parameter modulates how plastic the connections of the network are and, therefore, how long a memory lasts, it does not modulate how fast the network learns, since each pattern is always learned in a single shot.

For any pattern pair that has already been learned, retrieval can be tested by presenting the original input ${𝐱}^{\left[k\right]}$ and calculating the output as


$$\begin{array}{c} {\hat{y}}_{j}^{\left[k\right]}(P)=\Theta \left(\left(\sum_{i=1}^{N_{\text{in}}}J_{ji}^{\left[k+P\right]}x_{i}^{\left[k\right]}\right)-T_{\text{in}}^{\left[P\right]}\right) \end{array}$$ (29)

for $j=1,\ldots ,N_{\text{out}}$, where *P* is the number of subsequent patterns that have been learned since pattern *k*, and the activation threshold $T_{\text{in}}^{\left[P\right]}$ is chosen such that *M*_out_ output units are activated. To determine the decay of the memory signal, this calculated output ${\hat{𝐲}}^{\left[k\right]}(P)$ can be compared to the given output pattern ${𝐲}^{\left[k\right]}$. The larger *P*, the larger the Hamming distance between the two vectors:


$$\begin{array}{c} H({𝐲}^{\left[k\right]},{\hat{𝐲}}^{\left[k\right]}(P))=\sum_{j=1}^{N_{\text{out}}}{\left(y_{j}^{\left[k\right]}-{\hat{y}}_{j}^{\left[k\right]}(P)\right)}^{2}. \end{array}$$ (30)

We define the signal quality after *P* subsequent patterns as


$$\begin{array}{c} S_{P}:=H_{\text{avg}}-H({𝐲}^{\left[k\right]},{\hat{𝐲}}^{\left[k\right]}(P)), \end{array}$$ (31)

where $H_{\text{avg}}=2N_{\text{out}}f_{\text{out}}(1-f_{\text{out}})$ is the average Hamming distance between two random *f*_out_-sparse vectors of length *N*_out_.

The retrieval threshold is defined as


$$\begin{array}{c} T_{S}:=t_{S}H_{\text{avg}}, \end{array}$$ (32)

for a fixed $t_{S}\in (0,1)$. We call a pattern with $S_{P}\geq T_{S}$ retrievable and a pattern with $S_{P}<T_{S}$ not retrievable. The maximal number *P* for which $S_{P}\geq T_{S}$, if $S_{P}$ is averaged across many patterns, is defined as the capacity $P^{*}$ of the network.

### Probabilistic description of dendritic sums

To better understand the learning dynamics of the network and the decay of the quality of the memory signal, which is used to quantify the capacity, we derive a probabilistic description of the dendritic sums of output units (see also, e.g., [45,46,49–51]). The dendritic sum of the *j*-th output unit is its net input


$$\begin{array}{c} d:=\sum_{i=1}^{N_{\text{in}}}J_{ji}x_{i}. \end{array}$$ (33)

In accordance with [45], we introduce the following nomenclature: In general, a calculated output pattern ${\hat{y}}_{j}^{\left[k\right]}(P)$ differs from the target output pattern $y_{j}^{\left[k\right]}$. We call output units that are active (1) in the target output *genuine* output units and output units that are inactive (0) in the target output *spurious* units. In the calculated output, on the one hand, genuine units can be correctly active or incorrectly inactive. On the other hand, spurious units can be correctly inactive or incorrectly active. The terms *genuine* and *spurious* do not indicate whether the calculated activity of an output unit is 1 or 0, respectively, but whether it should be 1 or 0, respectively, to match the target output. In this section, we derive the distributions of the dendritic sums of genuine and spurious output units separately, and these dendritic sums are denoted by symbols $d_{s}$ (spurious) and $d_{g}$ (genuine). The shapes of these two distributions (especially their overlap) is related to the Hamming distance between target and calculated output and consequently also to the signal quality. These relationships will become clear later in the Methods, i.e., in section ‘Analytical calculation of the capacity of the network’.

With respect to a particular pattern pair, we further distinguish between four types of connections:

- genuine-genuine (g-g) connections connect active input to active output units,
- genuine-spurious (g-s) connections connect active input to inactive output units,
- spurious-genuine (s-g) connections connect inactive input to active output units, and
- spurious-spurious (s-s) connections connect inactive input to inactive output units.

Note that the neuronal activity that determines the connection type is here understood as the activity in the original pattern pair $\left({𝐱}^{\left[k\right]},{𝐲}^{\left[k\right]}\right)$ and not in the calculated output pattern ${\hat{𝐲}}^{\left[k\right]}(P)$.

#### Distributions of dendritic sums.

In what follows, we follow the work of [29,43,46] and calculate the distribution of the net input $d_{l}^{\left[P\right]}=J_{j-}^{\left[k+P\right]}{𝐱}^{\left[k\right]}$ to an output unit *j* where l∈{s,g} denotes a spurious (*s*) or a genuine (*g*) output unit. This input $d_{l}^{\left[P\right]}$ is also called the dendritic sum of output unit *j*. The term $J_{j-}^{\left[k+P\right]}$ is the *j*-th line of the weight matrix obtained for *P* additional patterns after learning pattern *k*. To understand how the *P* subsequent patterns change the wei*g*ht matrix, we have to take into account how often the output unit *j* is active across all *P* patterns, which is also called the output unit usage. As suggested by [45], we assume that the number *r* of times an output unit is active in *P* statistically independent patterns follows a binomial distribution $ℬ(P,f_{\text{out}})$, thus


𝒫(r=u)=ℬP,fout(u)=(Pu)foutu(1−fout)P−u. (34)

This describes the output unit usage more accurately than the assumption that each output unit is active exactly $f_{\text{out}}\cdot P$ times across a pattern set of size *P*, as assumed in the original paper on the Willshaw network [29] (also see [61]).

The dendritic sum of the output unit *j* gets contributions only from connections that originate at one of the *M*_in_ input units that are active in the *k*-th pattern. Independently of the state of a connection (functional or silent), connections from inactive input units do not contribute to the net input to an output unit. We define $\rho_{s}(u)$ as the probability that a genuine-spurious connection to the output unit *j* is functional given that the output unit *j* is active *u* times in the pattern set. Similarly, the probability that a genuine-genuine connection is functional is called $\rho_{g}(u)$. The functionality of a connection implicitly takes into account that the connection is available morphologically. We emphasize that $\rho_{l}(u)$, for l∈{s,g}, depends on whether the respective *output* unit is genuine ($\rho_{g}(u)$), i.e., active in the target output, or spurious ($\rho_{s}(u)$), i.e., inactive in the target output. The derivation of $\rho_{s}(u)$ and $\rho_{g}(u)$ is discussed in the S1 Appendix. Moreover, the probability that a spurious-spurious or a spurious-genuine connection is functional is less interesting because these connections do not contribute to dendritic sums due to lack of input. However, these probabilities must be considered implicitly in S1 Appendix to be able to determine $\rho_{s}(u)$ and $\rho_{g}(u)$. Further, the probability of a functional spurious-genuine connection, called $\rho_{n}(u)$, is important if the input pattern used for retrieval is noisy. This case will be discussed in Section ‘Noisy input patterns during retrieval’.

For a particular output unit usage *u* and statistically independent output units, the dendritic sums $d_{l}^{\left[P\right]}$ are binomially distributed with


𝒫(dl[P]=x | r=u)=ℬMin,ρl(u)(x)=(Minx)ρl(u)x(1−ρl(u))Min−x, (35)

for l∈{s,g}. In the extreme case where all connections from input units that are active in the *k*-th pattern to an output unit are functional, its dendritic sum equals the number of active input units *M*_in_. Combining Eq (34) and Eq (35), we obtain


𝒫(dl[P]=x)=∑u=0P(Pu)foutu(1−fout)P−u(Minx)ρl(u)x(1−ρl(u))Min−x, (36)

for l∈{s,g}, as the distribution of the dendritic sums $d_{l}^{\left[P\right]}$. This probability is described here for the sake of completeness, but it is the same as derived in [46]. However, the probabilities of functional connections $\rho_{l}$ in our work differ from those considered in [46] (see S1 Appendix).

In what follows, we abbreviate


$$\begin{array}{c} p_{s}^{\left[P\right]}(x):=𝒫(d_{s}^{\left[P\right]}=x) \end{array}$$ (37)

and


$$\begin{array}{c} p_{g}^{\left[P\right]}(x):=𝒫(d_{g}^{\left[P\right]}=x). \end{array}$$ (38)

Before including the probability $\rho_{l}(u)$ of a single genuine-genuine or a single genuine-spurious connection being functional after storing a set of additional patterns in which the output unit was active *u* times (derived in S1 Appendix), we review the learning rule from a probabilistic perspective and determine important fractions of connections.

#### Hebbian and homeostatic update rules from a probabilistic perspective.

We assume a morphological connectivity of $c_{m}\cdot N_{\text{in}}$ connections per output unit and start from a random choice of $c\cdot N_{\text{in}}$ functional connections targeting each output unit. In each learning step *k*, only connections targeting output units that are active in the current pattern, i.e., genuine-genuine and spurious-genuine connections, are updated. We further can distinguish between four types of connections: They can be either silent or functional before learning the *k*-th pattern and they can either originate from an active or an inactive input unit in the *k*-th pattern. Two types of connections are not updated: If the connection is already functional and the corresponding input unit is active (in addition to the corresponding output unit being active), the connection is protected and remains functional. If the connection is previously silent and the input unit is inactive, the connection always remains silent. The other two types can transition between the two states (functional/silent): Genuine-genuine connections (a fraction *f*_in_ of all connections to one output unit) of the *k*-th pattern that are previously silent (a fraction $c_{m}-c$) are made functional with a transition probability η, i.e., the fraction $f_{\text{in}}(c_{m}-c)\eta$ of all connections becomes functional in one learning step.

For each genuine output unit in the *k*-th pattern, the same number of connections that were made functional is also silenced such that there is a constant fraction *c* of functional connections per output unit after Hebbian learning and homeostatic normalization. Only spurious-genuine connections of the *k*-th pattern (fraction $1-f_{\text{in}}$) that are previously functional (fraction *c*) could be silenced due to the homeostasis mechanism. This is a total fraction $c(1-f_{\text{in}})$ of all connections. The probability that such a connection is silenced depends on the number of additional connections that became functional due to the Hebbian mechanism in the same learning step. As discussed above, a fraction $\eta \cdot (c_{m}-c)f_{\text{in}}$ becomes functional in each step. The probability for a previously functional connection from an inactive input unit to be silenced due to homeostasis is


$$\begin{array}{c} \frac{\eta f_{\text{in}}(c_{m}-c)}{c(1-f_{\text{in}})}. \end{array}$$ (39)

Normalization is possible in this way if this probability is less than or equal to one, which is always fulfilled if we assume that $c\geq f_{\text{in}}$ (regardless of the values of η and $c_{m}$).

In summary, the numerator in Eq 39 is the fraction of connections that are made functional in this step and the denominator is the fraction of connections available to be silenced; together, this is the *probability* to be newly silenced. Since the *fraction* of connections made functional, $\eta f_{\text{in}}(c_{m}-c)$, equals the *fraction* of connections newly silenced, $\frac{\eta f_{\text{in}}(c_{m}-c)}{c(1-f_{\text{in}})}\cdot c(1-f_{\text{in}})$, the overall fraction *c* of functional connections is maintained. Since patterns are uncorrelated, these probabilities remain the same for every pattern that is learned, and they will be used several times in the derivations in the following subsections.

#### Probabilities of functional connections.

As derived in detail in S1 Appendix, the probability for a connection to a spurious unit to be functional is


$$\begin{array}{c} \rho_{s}(u)=c,\;\text{for all }u\in \mathbb{N}. \end{array}$$ (40)

It does not change with the number of times *u* the output unit was active in other patterns. The probability $\rho_{g}$ that a connection to a genuine unit is functional, however, does strongly depend on *u*. It is given by


$$\begin{array}{c} \rho_{g}(u)=(c_{m}-c)\eta {\left(1-\frac{f_{\text{in}}\eta c_{m}}{c}\right)}^{u}+c. \end{array}$$ (41)

Thus, we can fully characterize the distributions of dendritic sums for spurious and genuine output units. We insert the probability of a functional connection to a spurious unit $\rho_{s}(u)=c$ into Eq (36), define $p_{s}(x):=p_{s}^{\left[0\right]}(x)$, and obtain the probability mass function (PMF) of the dendritic sum of a spurious output unit:


ps(x)=𝒫(ds=x)=∑u=0P(Pu)foutu(1−fout)P−u(Minx)cx(1−c)Min−x (42)

=(Minx)cx(1−c)Min−x (43)

$$\begin{array}{cc} = & {ℬ}_{M_{\text{in}},\;c}(x). \end{array}$$ (44)

Since $\rho_{s}(u)$ is constant as a function of the output unit usage *u*, the distribution of dendritic sums for spurious output units $p_{s}$ simplifies to a single binomial distribution ${ℬ}_{n,\;p}$ with parameters *n* = *M*_in_ and *p* = *c*, and $p_{s}$ does not depend on the number of subsequently learned patterns *P*.

Analogously, we insert $\rho_{g}(u)=(c_{m}-c)\eta {\left(1-\frac{f_{\text{in}}\eta c_{m}}{c}\right)}^{u}+c$ into Eq (36) and obtain the PMF of the dendritic sum of a genuine output unit


pg[P](x)=𝒫(dg[P]=x)=∑u=0P[(Pu)foutu(1−fout)P−u(Minx)((cm−c)η(1−finηcmc)u+c)x·(1−(cm−c)η(1−finηcmc)u−c)Min−x] (45)

$$\begin{array}{cc} & =\sum_{u=0}^{P}\left[{ℬ}_{P,\;f_{\text{out}}}(u)\cdot {ℬ}_{M_{\text{in}},\;\rho_{g}(u)}(x)\right]. \end{array}$$ (46)

**Fig 7:**
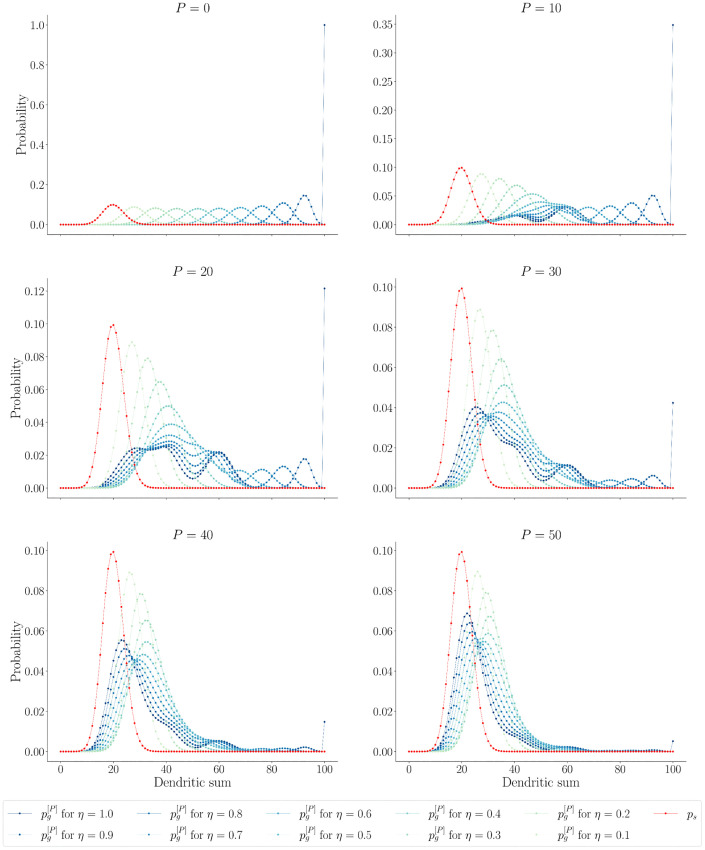
Distributions of dendritic sums. Analytical probability mass functions of dendritic sums after storing *P* = 0, 10, 20, 30, 40, 50 patterns (Eqs (44) and (46)). Spurious units in red; genuine units in blue shades, for several transition probabilities η. With an increasing number of subsequent patterns *P*, the distributions of the dendritic sums of genuine units approach the distribution of the dendritic sums of spurious units. The genuine distributions are multimodal, in particular for large transition probabilities η and small numbers of patterns *P* > 0. Other parameters: $N_{\text{in}}=N_{\text{out}}=1000,f_{\text{in}}=f_{\text{out}}=0.1,c=0.2,c_{m}=1$.

Thus, $p_{g}^{\left[P\right]}$ depends on the number of subsequent patterns *P* (in contrast to $p_{s}$). It is not a single binomial but a linear combination of binomial PMFs ${ℬ}_{M_{\text{in}},\;\rho_{g}(u)}(x)$, for u∈{0,…,P}, see Fig 7. This multimodality is analyzed in more detail in S2 Appendix (also see [61] for a comparison to a binomial distribution). The relation between $p_{s}$ and $p_{g}^{\left[P\right]}$ and their dependence on the activation ratios *f*_in_ and *f*_out_ is discussed in the Results, Section ‘Activation ratios in the distributions of dendritic sums’. S5 Fig shows a comparison of Eqs (44) and (46) to distributions of dendritic sums obtained from numerical simulations.

Let us finally discuss some limiting cases. We have


$$\begin{array}{c} f_{\text{in}}\eta c_{m}\leq f_{\text{in}}c_{m}\leq f_{\text{in}}\leq c \end{array}$$ (47)

because 0<η≤1, $0<c_{m}\leq 1$, 0 < *f*_in_, and $f_{\text{in}}\leq c$. Note that the constraint $f_{\text{in}}\leq c$ stems from the normalization condition as discussed previously in Section ‘Hebbian and homeostatic update rules from a probabilistic perspective’. For the term $1-\frac{f_{\text{in}}\eta c_{m}}{c}$ in Eq (41), it thus follows that we either have


$$\begin{array}{c} 0<1-\frac{f_{\text{in}}\eta c_{m}}{c}<1, \end{array}$$ (48)

which implies


$$\begin{array}{c} \rho_{g}(u)\overset{u\to \infty \;}{\to }c \end{array}$$ (49)

or, for $c_{m}=1$, η=1, and *f*_in_ = *c*,


$$\begin{array}{c} 1-\frac{f_{\text{in}}\eta c_{m}}{c}=0, \end{array}$$ (50)

which implies


$$\begin{array}{c} \rho_{g}(0)=1 \end{array}$$ (51)

$$\begin{array}{c} \rho_{g}(u)=c,\;\text{for }u>0. \end{array}$$ (52)

In the second case, the part of the memory stored in the connections targeting one output unit is completely lost as soon as this output unit is active one additional time for another pattern.

#### Noisy input patterns during retrieval.

So far we always assumed that during recall the original input pattern is shown to the network. Due to the inherent presence of noise in biological systems, it makes sense to assume that the input pattern presented to the network during retrieval is not exactly the same as the input pattern used for training. In the following, we discuss the consequences on the previous derivations when a noisy cue is presented to the network by putting noise onto the input pattern (cf. [46,47]). To be able to compare the results in a fair way, the input activation ratio *f*_in_ should not be changed, hence a fraction ε of the input units that are active in the original pattern (genuine input units) is deactivated while the same absolute number of input units that are inactive in the original pattern (spurious input units) is activated. Thus there is a number $m_{g}=M_{\text{in}}\cdot (1-\varepsilon )$ of original input units (genuine input units) and a number $m_{s}=M_{\text{in}}\cdot \varepsilon$ of wrong input units (spurious input units) active, in total we have $M_{\text{in}}=m_{g}+m_{s}$ active units (see [45,46]). The parameter ε is restricted to values that yield $m_{g},m_{s}\in \mathbb{N}$. Note that, in the numerical simulations, we set each of the genuine input units to zero with probability ε, count how many changed and flip the same amount of spurious input units to one. This means $m_{g}$ and $m_{s}$ are not fixed exact numbers but follow distributions. Only *on average*, $M_{\text{in}}\varepsilon$ genuine units are deactivated. The additional variability coming from deactivating each genuine input unit with probability ε would make the analytical description more involved while hardly affecting the expected result for large numbers. It is therefore neglected in the theory derived in this section. Instead, we assume that, for generating a noisy cue, exactly $M_{\text{in}}\varepsilon$ of the genuine input units are deactivated and $M_{\text{in}}\varepsilon$ of the spurious input units are activated. Note that, nevertheless, in both numerical simulations and theory, it is always ensured that the absolute number of active units *M*_in_ in the noisy input pattern presented to the network does not change.

Noisy cues do not alter the distribution of dendritic sums of spurious output units, but they do alter the distribution of dendritic sums of genuine output units. Regarding the latter, the probability of a functional genuine-genuine connection is the same as before, that is, $\rho_{g}$. But we now need to consider, in addition, the probability of a functional spurious-genuine connection, which is derived in S1 Appendix as


$$\begin{array}{c} \rho_{n}(u)=\frac{-f_{\text{in}}\eta (c_{m}-c)}{1-f_{\text{in}}}{\left(1-\frac{f_{\text{in}}\eta c_{m}}{c}\right)}^{u}+c. \end{array}$$ (53)

Similarly to $\rho_{g}(u)$, also $\rho_{n}(u)$ depends on the number of times *u* an output unit has been active across the pattern set. The probability of a functional spurious-genuine connection $\rho_{n}$ is lower than the probability of a functional genuine-genuine connection $\rho_{g}$ (and even lower than the functional connectivity *c*, see Figures in S1 Appendix).

For a particular output unit usage *u*, the dendritic sums $d_{g}^{\left[P\right]}$ are distributed following


**Fig 8:**
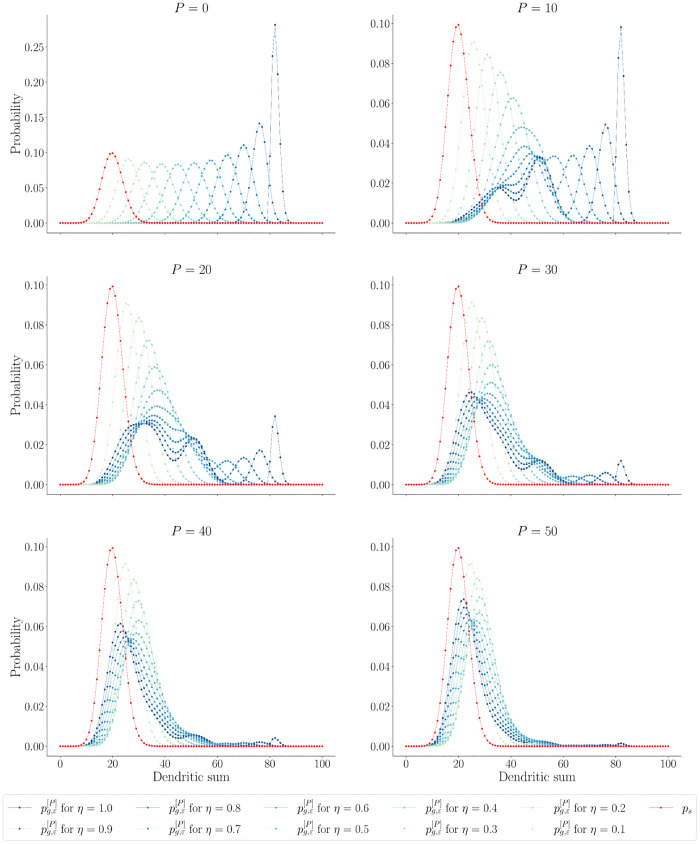
Distributions of dendritic sums with noise. Analytical probability mass functions of dendritic sums with noise on the input patterns during retrieval after storing *P* = 0, 10, 20, 30, 40, 50 patterns (Eq (56) with Eqs (41) and (53); Eq (36) with Eq (40)). Spurious units in red; genuine units in blue shades, for several transition probabilities η. With an increasing number of subsequent patterns *P*, the distributions of the dendritic sums of genuine units approach the distribution of the dendritic sums of spurious units. Compared to the noise-less case (see Fig 7), the genuine distributions are closer to the spurious distribution. Other parameters: $N_{\text{in}}=1000,f_{\text{in}}=0.1,N_{\text{out}}=1000,f_{\text{out}}=0.1,c=0.2,c_{m}=1,\varepsilon =0.2$. The same figure but with ε=0.4 can be found in S6 Fig.

𝒫(dg[P]=x | r=u)=∑xg=0x((mgxg)ρg(u)xg(1−ρg(u))mg−xg ·(msxs)ρn(u)xs(1−ρn(u))ms−xs), (54)

where $x_{s}=x-x_{g}$. In total, the probability of the dendritic sum $d_{g}^{\left[P\right]}$ of a genuine output unit to have a particular value *x* can be described by


pg,ε[P](x)=𝒫(dg[P]=x)=∑u=0P(Pu)[foutu(1−fout)P−u∑xg=0x((mgxg)ρg(u)xg(1−ρg(u))mg−xg·(msxs)ρn(u)xs(1−ρn(u))ms−xs)] (55)

$$\begin{array}{cc} & \;=\sum_{u=0}^{P}\left[{ℬ}_{P,f_{\text{out}}}(u)\sum_{x_{g}=0}^{x}\left({ℬ}_{m_{g},\rho_{g}(u)}(x_{g})\;{ℬ}_{m_{s},\rho_{n}(u)}(x_{s})\right)\right] \end{array}$$ (56)

as derived in [45] but, as an extension, with the probabilities of functional connections $\rho_{g}$ and $\rho_{n}$ derived in S1 Appendix that include the transition probability η and the homeostatic constraint of a fixed in-degree. Compared to the distribution of the dendritic sums for the noise-less input pattern, this distribution is shifted to the left. Thus, it is closer to the distribution of the dendritic sums of the spurious units, the Hamming distance between the target output and the calculated output pattern is larger, and thus the capacity decreases (compare Fig 8 and S6 Fig to Fig 7). The distribution of dendritic sums in Eq (56) will be used in Section ‘Extension to noisy input patterns during retrieval’ to derive the capacity for retrieval with noisy cues.

### Analytical calculation of the capacity of the network

In what follows, our aim is to analytically quantify the capacity of the network. The capacity is defined as the number of subsequent patterns *P* for which the signal quality has declined to the retrieval threshold. Therefore, we first discuss the signal quality as a function of *P*. Before we enter the details, we briefly recap basic quantities: The difference between a calculated output pattern ${\hat{𝐲}}^{\left[k\right]}(P)$ given a particular input pattern ${𝐱}^{\left[k\right]}$ after learning *P* subsequent pattern pairs and the corresponding target output pattern ${𝐲}^{\left[k\right]}$ is measured in terms of the Hamming distance between the two, denoted by $H\left({𝐲}^{\left[k\right]},{\hat{𝐲}}^{\left[k\right]}(P)\right)$. This Hamming distance is always averaged across a large number of patterns, and it lies between 0 and the average Hamming distance between two random patterns of length *N*_out_ and activation ratio *f*_out_, which is


$$\begin{array}{c} H_{avg}:=2N_{\text{out}}f_{\text{out}}(1-f_{\text{out}}). \end{array}$$ (57)

**Fig 9:**
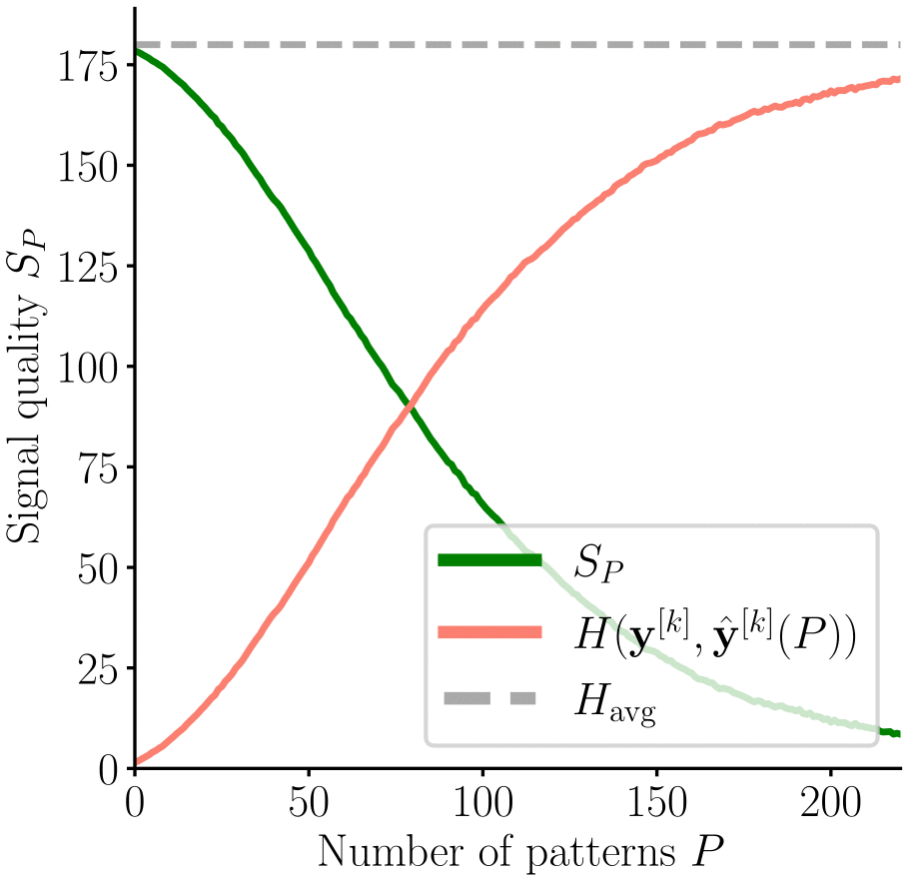
Hamming distance and signal quality. The Hamming distance $H\left({𝐲}^{\left[k\right]},{\hat{𝐲}}^{\left[k\right]}(P)\right)$ between the target and the calculated output pattern (salmon line) increases with an increasing number of patterns *P* and approaches *H*_avg_ (gray line) for P→∞. The signal quality $S_{P}$ (green line) decreases with increasing *P* and approaches 0.

We define the memory signal quality $S_{P}$ at *P* patterns as the difference between $H_{avg}$ and the Hamming distance between the target output and the calculated output $H\left({𝐲}^{\left[k\right]},{\hat{𝐲}}^{\left[k\right]}(P)\right)$:


$$\begin{array}{c} S_{P}:=H_{avg}-H\left({𝐲}^{\left[k\right]},{\hat{𝐲}}^{\left[k\right]}(P)\right), \end{array}$$ (58)

see Fig 9, green curve. The signal quality can be expressed as a fraction $s_{P}$ of *H*_avg_


$$\begin{array}{c} S_{P}=s_{P}\cdot H_{avg},\;\text{where }s_{P}\in [0,1]. \end{array}$$ (59)

We call $s_{P}$ the normalized signal quality. With $s_{P}$, the Hamming distance reads as


$$\begin{array}{c} H\left({𝐲}^{\left[k\right]},{\hat{𝐲}}^{\left[k\right]}(P)\right)=\left(1-s_{P}\right)\cdot H_{avg}=\overset{―}{s_{P}}\cdot H_{avg} \end{array}$$ (60)

(see Fig 9, salmon line), where we introduce the additional symbol


$$\begin{array}{c} \overset{―}{s_{P}}:=1-s_{P} \end{array}$$ (61)

out of convenience for the derivations in this section.

#### Analytical Hamming distance.

In this section, we derive the expected Hamming distance $𝔼\left(H\left({𝐲}^{\left[k\right]},{\hat{𝐲}}^{\left[k\right]}(P)\right)\right)$ between the target output ${𝐲}^{\left[k\right]}$ and the output ${\hat{𝐲}}^{\left[k\right]}(P)$ calculated after learning *P* additional patterns. We follow a similar approach as several earlier works (e.g., [45–47,51,52,61]): The distributions of dendritic sums allow us to describe the error in retrieval (expressed by the Hamming distance) via the probabilities of wrongly active and wrongly inactive units. However, the constraint of a fixed output sparseness adds a layer of complexity to our derivations (see the following Subsection ‘The activation threshold and balancing tails of the distributions’).

*The activation threshold and balancing tails of the distribution.* Since the Hamming distance depends on the activation threshold $T_{\text{in}}^{\left[P\right]}$, we first discuss the choice of $T_{\text{in}}^{\left[P\right]}$, which is self-consistently defined such that the correct number of output units, *M*_out_, is activated:


$$\begin{array}{c} \sum_{j=1}^{N_{\text{out}}}{\hat{y}}_{j}^{\left[k\right]}(P)\overset{!}{=}M_{\text{out}}. \end{array}$$ (62)

The activation of output unit *j* is calculated as


$$\begin{array}{c} {\hat{y}}_{j}^{\left[k\right]}(P)=\Theta \left(\left(\sum_{i=1}^{N_{\text{in}}}J_{ji}^{\left[k+P\right]}x_{i}^{\left[k\right]}\right)-T_{\text{in}}^{\left[P\right]}\right)=\Theta \left({\left(J^{\left[k+P\right]}{𝐱}^{\left[k\right]}\right)}_{j}-T_{\text{in}}^{\left[P\right]}\right), \end{array}$$ (63)

where $\Theta (x)=\left\{ \begin{array}{c} 1,\;\text{for }x\geq 0\\ 0,\;\text{for }x<0 \end{array}\right.$ is the Heaviside step function. The number of active output units is hence


$$\begin{array}{c} \sum_{j=1}^{N_{\text{out}}}{\hat{y}}_{j}^{\left[k\right]}(P)=\sum_{j=1}^{N_{\text{out}}}\Theta \left({\left(J^{\left[k+P\right]}{𝐱}^{\left[k\right]}\right)}_{j}-T_{\text{in}}^{\left[P\right]}\right). \end{array}$$ (64)

Since the weight matrix *J*^[*k*+*P*]^ is updated in every learning step and hence depends on the number of subsequently learned patterns *P*, the activation threshold $T_{\text{in}}^{\left[P\right]}$ also depends on *P*. It thus has to be newly determined in every update step in order to obtain the given number of active output units *M*_out_.

**Fig 10:**
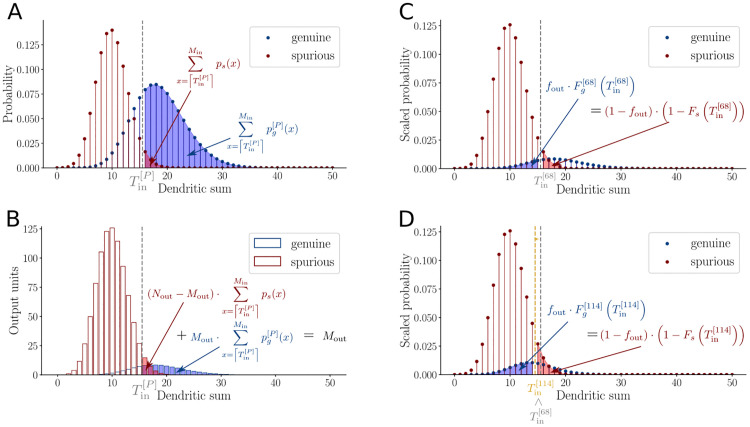
The activation threshold and balancing tails of the distributions. (**A**) Distributions of dendritic sums of genuine (blue) and spurious (red) output units. Output units with a dendritic sum larger than the activation threshold $T_{\text{in}}^{\left[P\right]}$ are activated. (**B**) Distributions of dendritic sums scaled by the number of units whose dendritic sums follow the respective distributions. The sum of the number of genuine (blue) and the number of spurious (red) units that have a dendritic sum larger than the threshold $T_{\text{in}}^{\left[P\right]}$ corresponds to the predefined number of active output units *M*_out_. (**C**) Distributions of dendritic sums of genuine (blue) and spurious (red) units scaled by *f*_out_ and $1-f_{\text{out}}$, respectively. The mass of the part of the genuine distribution left of the activation threshold $T_{\text{in}}^{\left[P\right]}$ (blue area) must correspond to the mass of the part of the spurious distribution right of $T_{\text{in}}^{\left[P\right]}$ (red area). (**D**) Same as (**C**) but for larger number of patterns *P* = 114 instead of *P* = 68. The threshold $T_{\text{in}}^{\left[114\right]}$ ($<T_{\text{in}}^{\left[68\right]}$) moves to the left in order to maintain the balance of the tails, which ensures the right amount of active output units.

The active output units can either be genuine, hence active in the target output pattern, or spurious, hence inactive in the target output pattern. The dendritic sum


$$\begin{array}{c} {\left(d_{g}^{\left[P\right]}\right)}_{j}={\left(J^{\left[k+P\right]}{𝐱}^{\left[k\right]}\right)}_{j}\sim p_{g}^{\left[P\right]} \end{array}$$ (65)

of a genuine output unit *j* follows the genuine distribution $p_{g}^{\left[P\right]}$ (Eq (46)). A genuine output unit is activated if its dendritic sum is at least as large as the activation threshold $T_{\text{in}}^{\left[P\right]}$, hence if


$$\begin{array}{c} {\left(J^{\left[k+P\right]}{𝐱}^{\left[k\right]}\right)}_{j}\geq T_{\text{in}}^{\left[P\right]} \end{array}$$ (66)

(blue area in Fig 10A). There is a total amount of *M*_out_ genuine output units. Thus, if we assume $M_{\text{out}}\gg 1$, the number of genuine output units that are activated for a given threshold $T_{\text{in}}^{\left[P\right]}$ can be approximated by using the law of large numbers as


$$\begin{array}{cc} \sum_{j\in G}{\hat{y}}_{j}^{\left[k\right]}(P) & \approx M_{\text{out}}\cdot {\left\langle \Theta \left({\left(d_{g}^{\left[P\right]}\right)}_{j}-T_{\text{in}}^{\left[P\right]}\right)\right\rangle }_{{\left(d_{g}^{\left[P\right]}\right)}_{j}\sim p_{g}^{\left[P\right]}} \end{array}$$ (67)

$$\begin{array}{cc} & =M_{\text{out}}\cdot \sum_{x=\left\lceil T_{\text{in}}^{\left[P\right]}\right\rceil }^{M_{\text{in}}}p_{g}^{\left[P\right]}(x), \end{array}$$ (68)

where *G* is the set of indices of genuine output units. Analogously, assuming $N_{\text{out}}-M_{\text{out}}\gg 1$, the number of spurious units that are activated can be approximated as


$$\begin{array}{cc} \sum_{j\in S}(y_{j}^{\left[P\right]}) & \approx (N_{\text{out}}-M_{\text{out}})\cdot {\left\langle \Theta \left({\left(d_{s}\right)}_{j}-T_{\text{in}}^{\left[P\right]}\right)\right\rangle }_{{\left(d_{s}\right)}_{j}\sim p_{s}} \end{array}$$ (69)

$$\begin{array}{cc} & =(N_{\text{out}}-M_{\text{out}})\cdot \sum_{x=\left\lceil T_{\text{in}}^{\left[P\right]}\right\rceil }^{M_{\text{in}}}p_{s}(x), \end{array}$$ (70)

where ${\left(d_{s}\right)}_{j}$ denotes the dendritic sum of the *j*-th output unit, which is spurious, and *S* is the set of indices of spurious output units (cf. red area in Fig 10A). In order to activate the right amount of output units, the activation threshold $T_{\text{in}}^{\left[P\right]}$ thus has to be chosen such that


$$\begin{array}{c} M_{\text{out}}\cdot \sum_{x=\left\lceil T_{\text{in}}^{\left[P\right]}\right\rceil }^{M_{\text{in}}}p_{g}^{\left[P\right]}(x)+(N_{\text{out}}-M_{\text{out}})\cdot \sum_{x=\left\lceil T_{\text{in}}^{\left[P\right]}\right\rceil }^{M_{\text{in}}}p_{s}(x)=M_{\text{out}}, \end{array}$$ (71)

see Fig 10B. By basic computations, we have that


$$\begin{array}{cc} M_{\text{out}}\cdot \sum_{x=0}^{\left\lfloor T_{\text{in}}^{\left[P\right]}\right\rfloor }p_{g}^{\left[P\right]}(x) & =(N_{\text{out}}-M_{\text{out}})\cdot \sum_{x=\left\lceil T_{\text{in}}^{\left[P\right]}\right\rceil }^{M_{\text{in}}}p_{s}(x) \end{array}$$ (72)

$$\begin{array}{cc} \Leftrightarrow f_{\text{out}}\cdot F_{g}^{\left[P\right]}\left(T_{\text{in}}^{\left[P\right]}\right) & =(1-f_{\text{out}})\cdot \left(1-F_{s}\left(T_{\text{in}}^{\left[P\right]}\right)\right), \end{array}$$ (73)

where $F_{g}^{\left[P\right]}$ and $F_{s}$ are the cumulative distribution functions (CDF) of $p_{g}^{\left[P\right]}$ and $p_{s}$, respectively (Fig 10C). We call the last equation the Balance Equation because it balances the false positive and the false negative errors.

With an increasing number of patterns *P*, the center of mass of the genuine distribution $p_{g}^{\left[P\right]}$ moves to the left, while the spurious distribution stays constant. For a fixed *T*_in_, the left-hand side of Eq (73) would hence grow while the right-hand side would not, which would lead to an output activation ratio that is smaller than *f*_out_. In order to keep the Balance Equation fulfilled, the activation threshold $T_{\text{in}}^{\left[P\right]}$ has to move to the left for increasing *P*; see Fig 10C for *P* = 68 and Fig 10D for *P* = 114.

*Deriving the Hamming distance from dendritic sums.* The Hamming distance


$$\begin{array}{c} H\left({𝐲}^{\left[k\right]},{\hat{𝐲}}^{\left[k\right]}(P)\right):=\#\left\{j\in \{0,\ldots ,N_{\text{out}}\}|y_{j}^{\left[k\right]}\neq {\left({\hat{y}}^{\left[k\right]}(P)\right)}_{j}\right\} \end{array}$$ (74)

is the sum of the number of genuine units that are wrongly set to 0 and the number of spurious units that are wrongly set to 1 in the calculated output ${\hat{𝐲}}^{\left[k\right]}(P)$:


$$\begin{array}{c} H\left({𝐲}^{\left[k\right]},{\hat{𝐲}}^{\left[k\right]}(P)\right)=\sum_{j\in G}\left(1-{\hat{y}}_{j}^{\left[k\right]}(P)\right)+\sum_{j\in S}{\hat{y}}_{j}^{\left[k\right]}(P). \end{array}$$ (75)

**Fig 11:**
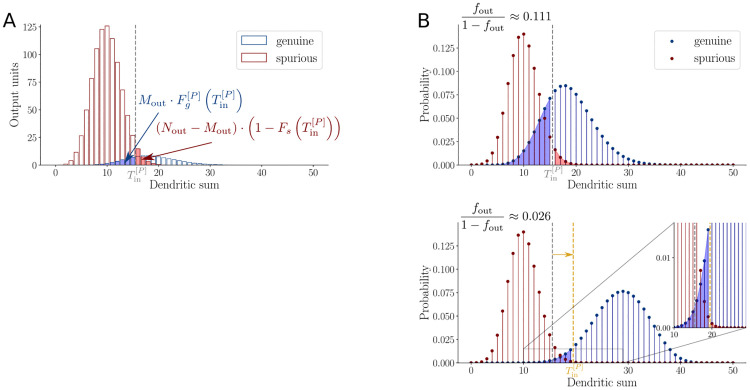
Deriving the Hamming distance from dendritic sums. (**A**) Hamming distance is the sum of falsely deactivated genuine units (blue area) and falsely activated spurious units (red area). (**B**) If *f*_out_ is changed, the threshold $T_{\text{in}}^{\left[P\right]}$ needs to be adapted such that the ratio of the red area to the blue area corresponds to the ratio $f_{\text{out}}/(1-f_{\text{out}})$. Upper: $f_{\text{out}}=0.1,f_{\text{out}}/(1-f_{\text{out}})\approx 0.111$, lower: $f_{\text{out}}=0.025,f_{\text{out}}/(1-f_{\text{out}})\approx 0.026$ and hence the red area is much smaller compared to the blue area in the lower panel than in the upper panel. This is achieved by a larger $T_{\text{in}}^{\left[P\right]}$, which can also affect the Hamming distance.

In terms of the distributions $p_{s}$ and $p_{g}^{\left[P\right]}$, the expected Hamming distance reads as


$$\begin{array}{cc} 𝔼\left(H\left({𝐲}^{\left[k\right]},{\hat{𝐲}}^{\left[k\right]}(P)\right)\right)= & \;M_{\text{out}}\cdot {\left\langle 1-\Theta \left({\left(d_{g}^{\left[P\right]}\right)}_{j}-T_{\text{in}}^{\left[P\right]}\right)\right\rangle }_{{\left(d_{g}^{\left[P\right]}\right)}_{j}\sim p_{g}^{\left[P\right]}}\\ & +(N_{\text{out}}-M_{\text{out}})\cdot {\left\langle \Theta \left({\left(d_{s}\right)}_{j}-T_{\text{in}}^{\left[P\right]}\right)\right\rangle }_{{\left(d_{s}\right)}_{j}\sim p_{s}} \end{array}$$ (76)

$$\begin{array}{cc} & =M_{\text{out}}F_{g}^{\left[P\right]}\left(T_{\text{in}}^{\left[P\right]}\right)+(N_{\text{out}}-M_{\text{out}})\left(1-F_{s}\left(T_{\text{in}}^{\left[P\right]}\right)\right), \end{array}$$ (77)

see Fig 11A. The Hamming distance is hence the sum of the part of the genuine distribution that lies below the activation threshold $T_{\text{in}}^{\left[P\right]}$ and the part of the spurious distribution that lies above $T_{\text{in}}^{\left[P\right]}$ — weighted by the corresponding number of units. Using the Balance Equation (73), we can express $F_{g}^{\left[P\right]}\left(T_{\text{in}}^{\left[P\right]}\right)$ in terms of $F_{s}\left(T_{\text{in}}^{\left[P\right]}\right)$, and the expected Hamming distance simplifies to


$$\begin{array}{c} 𝔼\left(H\left({𝐲}^{\left[k\right]},{\hat{𝐲}}^{\left[k\right]}(P)\right)\right)=2N_{\text{out}}(1-f_{\text{out}})\left(1-F_{s}\left(T_{\text{in}}^{\left[P\right]}\right)\right). \end{array}$$ (78)

The dependence on the number of patterns *P* is now solely contained in the activation threshold $T_{\text{in}}^{\left[P\right]}$ because $F_{s}$ is constant in *P*. The Hamming distance increases with decreasing $T_{\text{in}}^{\left[P\right]}$ and, as discussed in the previous Section ‘The activation threshold and balancing tails of the distributions’, $T_{\text{in}}^{\left[P\right]}$ decreases with increasing *P*. Thus, the larger the number of subsequently learned patterns *P*, the larger the Hamming distance between target output and calculated output and, according to Eq (58), the smaller the signal quality $S_{P}$.

Note that the dependence of the Hamming distance on the output activation ratio *f*_out_ is less straightforward. On the one hand, if *f*_out_ is increased, the factor $1-f_{\text{out}}$ in Eq (78) yields a decrease in the Hamming distance. On the other hand, an increase of *f*_out_ also decreases the activation threshold $T_{\text{in}}^{\left[P\right]}$ and thus increases the factor $1-F_{s}(T_{\text{in}}^{\left[P\right]})$ in Eq (78), which yields an increase in the Hamming distance (see Fig 11B). It is not easy to determine analytically which of these opposing effects is stronger. As mentioned in the Results in Section ‘Activation ratios in the distributions of dendritic sums’, we speculate that the combined effect on the Hamming distance is small in the parameter range that we investigated numerically.

#### Signal quality after *P* subsequent patterns.

In order to derive the signal quality $S_{P}=s_{P}H_{\text{avg}}$ (see Eqs (58) and (59)) after *P* additional learning steps, we combine Eqs (60) and (78) and obtain


$$\begin{array}{c} \overset{―}{s_{P}}\cdot H_{\text{avg}}=2N_{\text{out}}(1-f_{\text{out}})(1-F_{s}(T_{\text{in}}^{\left[P\right]})). \end{array}$$ (79)

Inserting $H_{\text{avg}}:=2N_{\text{out}}f_{\text{out}}(1-f_{\text{out}})$ yields


$$\begin{array}{cc} \overset{―}{s_{P}}f_{\text{out}} & =1-F_{s}(T_{\text{in}}^{\left[P\right]}) \end{array}$$ (80)

$$\begin{array}{cc} \Leftrightarrow T_{\text{in}}^{\left[P\right]} & =F_{s}^{-1}\left(1-\overset{―}{s_{P}}f_{\text{out}}\right). \end{array}$$ (81)

Using Eq (80), we find that the Balance Equation (73) is fulfilled if and only if


$$\begin{array}{cc} F_{g}^{\left[P\right]}(T_{\text{in}}^{\left[P\right]}) & =\overset{―}{s_{P}}(1-f_{\text{out}}) \end{array}$$ (82)

$$\begin{array}{cc} \Leftrightarrow T_{\text{in}}^{\left[P\right]} & ={F_{g}^{\left[P\right]}}^{-1}\left(\overset{―}{s_{P}}(1-f_{\text{out}})\right). \end{array}$$ (83)

**Fig 12:**
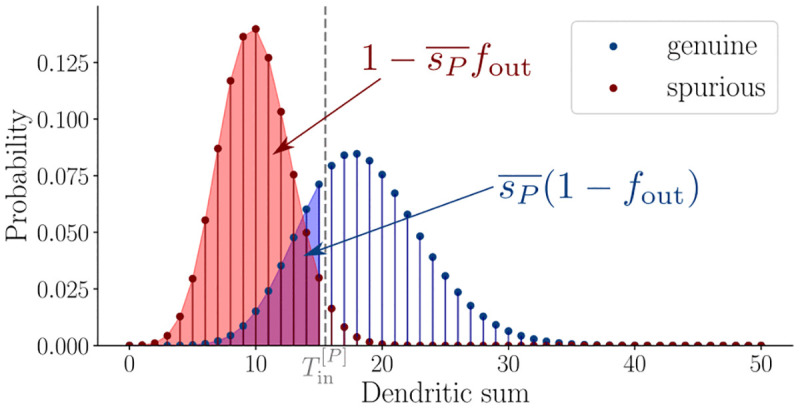
Signal Quality Equation. The activation threshold $T_{\text{in}}^{\left[P\right]}$ corresponds to the $1-\overset{―}{s_{P}}f_{\text{out}}$-quantile of the spurious distribution and to the $\overset{―}{s_{P}}(1-f_{\text{out}})$-quantile of the genuine distribution.

Fig 12 illustrates the two equations. We can equate the right-hand sides of Eqs (81) and (83) to get rid of the explicit value of $T_{\text{in}}^{\left[P\right]}$ and obtain a single equation


$$\begin{array}{c} F_{s}^{-1}\left(1-\overset{―}{s_{P}}f_{\text{out}}\right)={F_{g}^{\left[P\right]}}^{-1}\left(\overset{―}{s_{P}}(1-f_{\text{out}})\right), \end{array}$$ (84)

which we call the Signal Quality Equation. If this equation is solved for $\overset{―}{s_{P}}$, inserting its solution into Eq (59) with $s_{P}=1-\overset{―}{s_{P}}$ determines the signal quality $S_{P}$. There is an explicit solution if $F_{s}^{-1}$ and $F_{g}^{-1}$ are easy to calculate.

Notably, since the CDF of a discrete probability distribution is a piecewise constant function, the CDFs in Eqs (81) and (83) are not generally invertible. A strict inverse $F^{-1}(y)$ such that $F(F^{-1}(y))=y$ does not always exist because the same CDF value *F*(*x*) can correspond to multiple arguments *x*. Being aware of this fact, we use the notation $F_{s}^{-1}$ and ${F_{g}^{\left[P\right]}}^{-1}$ here anyway. Later in this section, the binomial distributions (see Eqs (44) and (46)) are approximated by normal distributions (also see S4 Appendix), and their approximated CDFs become strictly increasing and continuous, and thus invertible.

Details regarding an approximate analytical solution and a numerical solution of the Signal Quality Equation (84) are provided in S3 Appendix.

#### Memory capacity of the network.

Instead of calculating the signal quality at any *P* subsequent patterns (see S3 Appendix), which can then be used for finding the *P* for which the signal quality reaches the retrieval threshold, the network capacity can be determined by directly equating the signal quality to the retrieval threshold (expressed in terms of $s_{P}$) and solving the resulting equation for *P*.

We are searching for the number of patterns *P* for which the signal quality as defined in Eq (58) equals the retrieval threshold $T_{S}$, which can be expressed as a fraction $t_{S}$ of *H*_avg_:


$$\begin{array}{c} S_{P}:=H_{avg}-H\left({𝐲}^{\left[k\right]},{\hat{𝐲}}^{\left[k\right]}(P)\right)\overset{!}{=}t_{S}H_{\text{avg}}=:T_{S}, \end{array}$$ (85)

with $t_{S}\in (0,1)$. In analogy to $\overset{―}{s_{P}}=1-s_{P}$, we define $\overset{―}{t_{S}}:=1-t_{S}$ for convenience. Instead of calculating $\overset{―}{s_{P}}$ for a given *P*, we now use the Signal Quality Equation (84) to find the number of patterns *P* for which $\overset{―}{s_{P}}\equiv \overset{―}{t_{S}}$ (which is equivalent to $s_{P}\equiv t_{S}$) and $\overset{―}{t_{S}}$ is given. We thus want to solve the Capacity Equation


$$\begin{array}{c} F_{s}^{-1}\left(1-\overset{―}{t_{S}}f_{\text{out}}\right)={F_{g}^{\left[P\right]}}^{-1}\left(\overset{―}{t_{S}}(1-f_{\text{out}})\right) \end{array}$$ (86)

for *P*, which is the only unknown in this equation for a fixed set of network parameters. Notably, the Signal Quality Equation (84) and the Capacity Equation (86) are of the same form but, in the first, the number of patterns *P* is given and the fraction $\overset{―}{s_{P}}$ is unknown, which determines the signal quality $S_{P}=(1-\overset{―}{s_{P}}H_{\text{avg}})$ while, in the second, the retrieval ratio $\overset{―}{t_{S}}$ is given and the number of patterns *P* is unknown, which corresponds to the capacity.

In order to solve the Capacity Equation (86), we first approximate the linear combination of binomial PMFs in $p_{g}^{\left[P\right]}$ by a single binomial PMF and we then approximate this binomial distribution as well as $p_{s}$ by normal distributions (see S4 Appendix for details). Regarding the first approximation step, [61] provides conditions for when this type of distribution converges to a binomial distribution and finds that a binomial distribution is a good approximation for a wide range of parameters, even though it can slightly overestimate storage capacity due to an underestimation of retrieval errors. In S4 Appendix, we present an empirical analysis of the quality of this approximation in dependence of the parameters of the model used here. For the second approximation step, the two assumptions


$$\begin{array}{c} M_{\text{in}}c\gg 1\text{ and }M_{\text{in}}(1-\rho_{g}(\lfloor f_{\text{out}}(P+1)\rfloor ))\gg 1 \end{array}$$ (87)

need to be respected (more details in S4 Appendix). We define the function


$$\begin{array}{c} R(x,y):={erf}^{-1}(1-2xy) \end{array}$$ (88)

and introduce the abbreviations


$$\begin{array}{c} R_{s}:=R(\overset{―}{t_{S}},f_{\text{out}})={erf}^{-1}\left(1-2\overset{―}{t_{S}}f_{\text{out}}\right) \end{array}$$ (89)

and


$$\begin{array}{c} R_{g}:=R(\overset{―}{t_{S}},1-f_{\text{out}})=-{erf}^{-1}\left(2\overset{―}{t_{S}}(1-f_{\text{out}})-1\right). \end{array}$$ (90)

Then the mentioned approximation steps allow us to express Eq (86) as


$$\begin{array}{c} \frac{{\bar{\mu }}_{g}^{\left[P\right]}-\mu_{s}}{\sqrt{2}\left(\sigma_{s}\cdot R_{s}+{\bar{\sigma }}_{g}^{\left[P\right]}\cdot R_{g}\right)}=1. \end{array}$$ (91)

Solving Eq (91) for *P* (for details see S4 Appendix) yields the capacity


$$\begin{array}{c} P^{*}=\left\lfloor \frac{\ln\left(\frac{A}{\eta B}\right)}{\ln\left(1-\frac{f_{\text{in}}\eta c_{m}}{c}\right)f_{\text{out}}}+\frac{1}{2}\right\rfloor . \end{array}$$ (92)

where


$$\begin{array}{cc} A:\;= & \;(1-2c)R_{g}^{2}+\sqrt{2M_{\text{in}}c(1-c)}R_{s}\\ & +|R_{g}|\cdot \sqrt{R_{g}^{2}+2c(1-c)(M_{\text{in}}-2R_{s}^{2})+2(1-2c)\sqrt{2M_{\text{in}}c(1-c)}R_{s}}, \end{array}$$ (93)

$$\begin{array}{c} B:\;=(c_{m}-c)(M_{\text{in}}+2R_{g}^{2}). \end{array}$$ (94)

If $R_{g}$ and $R_{s}$ are known, this expression allows us to directly calculate the capacity of the network. The approximation of the error function used for calculating $R_{g}$ and $R_{s}$ can be found in S4 Appendix.

#### Optimal transition probability and maximal capacity.

From Eq (92), we can derive the optimal transition probability $\eta_{\text{opt}}$ and therewith the maximal capacity $P_{\text{max}}^{*}$ for a fixed set of network parameters.

We assume $\frac{f_{\text{in}}\eta c_{m}}{c}\ll 1$ and use the Taylor series of ln(1−x) at *x* = 0 to approximate


$$\begin{array}{c} \ln\left(1-\frac{f_{\text{in}}\eta c_{m}}{c}\right)\approx -\frac{f_{\text{in}}\eta c_{m}}{c}. \end{array}$$ (95)

The capacity as a function of η then reads as


$$\begin{array}{c} P^{*}(\eta )\approx \left\lfloor \frac{\ln(A)-\ln(B)-\ln(\eta )}{-\eta C}+\frac{1}{2}\right\rfloor , \end{array}$$ (96)

with *A* and *B* as defined in Eqs (93) and (94) and


$$\begin{array}{c} C:=\frac{M_{\text{in}}c_{m}f_{\text{out}}}{N_{\text{in}}c}. \end{array}$$ (97)

In the following, we will ignore the rounding of $P^{*}$ to an integer value in (96) and allow the capacity to take any (non-negative) real value. We then have


$$\begin{array}{c} P^{*}(\eta )\approx \frac{\ln(A)-\ln(B)-\ln(\eta )}{-\eta C} \end{array}$$ (98)

as also stated in the Results in Eq (21). Next, we calculate the optimal transition probability $\eta_{opt}$ by differentiating $P^{*}$ with respect to η


$$\begin{array}{c} {P^{*}}^{\prime }(\eta )=\frac{-\ln(\eta )+\ln(A)-\ln(B)+1}{\eta^{2}C} \end{array}$$ (99)

and solving


$$\begin{array}{cc} & {P^{*}}^{\prime }(\eta_{0})=0 \end{array}$$ (100)

$$\begin{array}{cc} \Leftrightarrow \; & \eta_{0}=\exp\left(1+\ln(A)-\ln(B)\right)=\frac{eA}{B}. \end{array}$$ (101)

This is a global maximum of $P^{*}(\eta )$ because


$$\begin{array}{c} {P^{*}}^{\prime \prime }(\eta_{0})=\frac{2\ln(\frac{eA}{B})-2\ln(A)+2\ln(B)-3}{{\left(\frac{eA}{B}\right)}^{3}C}=\frac{-1}{{\left(\frac{eA}{B}\right)}^{3}C}<0. \end{array}$$ (102)

**Fig 13:**
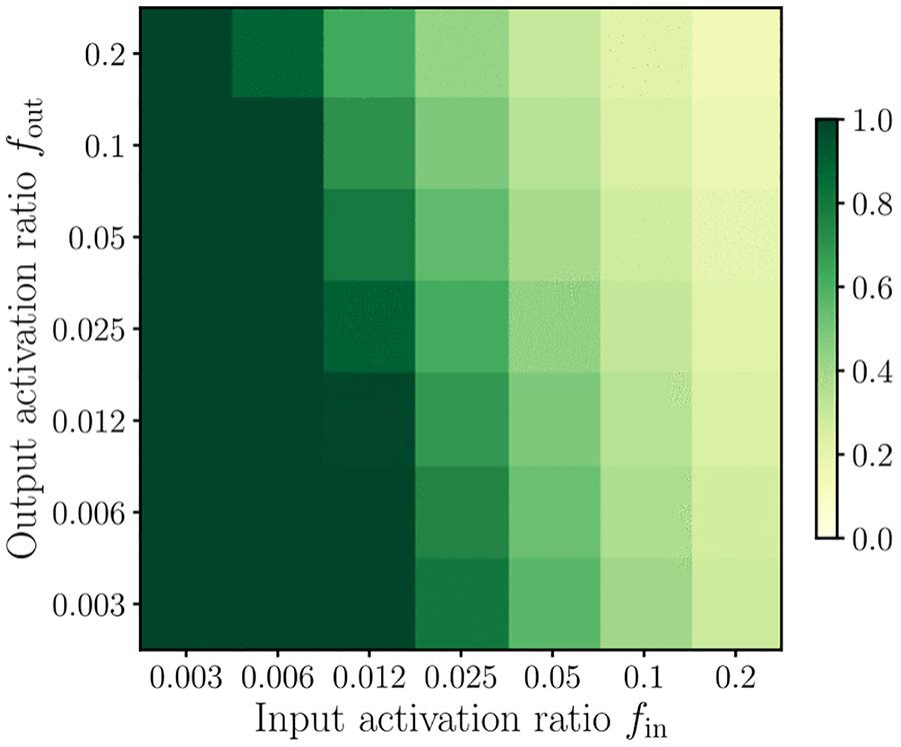
Analytical optimal transition probability. Optimal transition probability $\eta_{\text{opt}}$ (Eq (103)), color coded, as a function of *f*_in_ and *f*_out_. The probability $\eta_{\text{opt}}$ decreases both as a function of *f*_in_ and as a function of *f*_out_ but the slope is larger for *f*_in_. For comparison, in Fig 3E in the Results, the corresponding optimal transition probability $\eta_{\text{opt}}$ obtained from numerical simulations is shown. Other parameters: $N_{\text{in}}=1000,c=0.2,c_{m}=1,t_{S}=0.5$..

The optimal transition probability must be bounded to (0,1]. We can assume that $\frac{eA}{B}>0$ (otherwise there is no real solution for Eq (92)). If $\frac{eA}{B}\leq 1$, we have $\eta_{opt}=\frac{eA}{B}$. If $\frac{eA}{B}>1$, the capacity as a function of the transition probability $P^{*}(\eta )$ is monotonically increasing in the range η∈(0,1] that we are interested in because we have shown that $P^{*}$ has a global maximum at eA/B>1. We thus have


$$\begin{array}{c} \eta_{opt}=\min\left(\frac{eA}{B},1\right), \end{array}$$ (103)

as also displayed in Eq (22) in the Results. Fig 13 shows $\eta_{\text{opt}}$ as a function of the input and output activation ratios *f*_in_ and *f*_out_.

The maximal capacity that can be obtained for a given set of network parameters by optimizing the transition probability is then defined as


$$\begin{array}{c} P_{\text{max}}^{*}:=P^{*}(\eta_{\text{opt}})\approx \frac{\ln(A)-\ln(B)-\ln(\eta_{\text{opt}})}{-\eta_{\text{opt}}C}. \end{array}$$ (104)

Comparisons of the analytical approximation of the optimal transition probability $\eta_{\text{opt}}$ (Eq (103)) and the maximal capacity $P_{\text{max}}^{*}$ (Eq (104)) with results obtained from numerical network simulations (cf. Section ‘Network simulations’) are presented in Figs 3B, 3C, 5.

**Fig 14:**
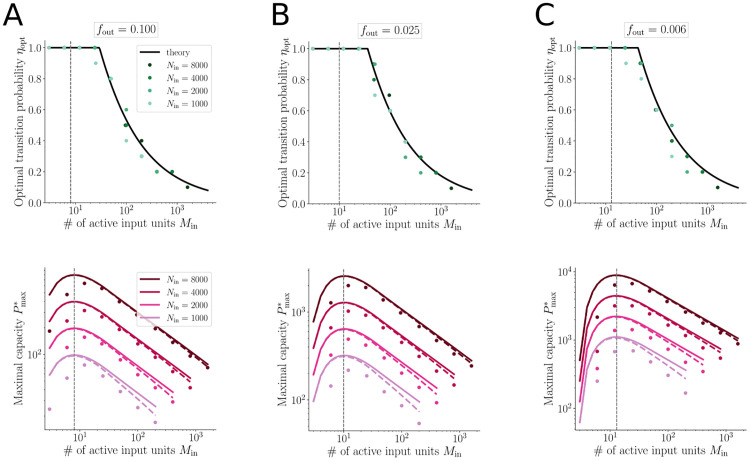
Optimal transition probability and maximal capacity for various *N*_in_. Dependence of optimal transition probability (top) and maximal capacity (bottom) on number of active input units ($M_{\text{in}}=f_{\text{in}}N_{\text{in}}$) for various input layer sizes ($N_{\text{in}}\in \{1000,2000,4000,8000\}$). Dots: numerical simulations; solid lines: optimal η obtained from Eq (103) and maximal capacity calculated with Eq (98); dashed lines: maximal capacity calculated with Eq (92), i.e., without the Taylor approximation (95). In (**A**), *f*_out_ = 0.1, in (**B**), *f*_out_ = 0.025 and in (**C**), *f*_out_ = 0.006. Other parameters: $N_{\text{out}}=1000,c=0.2,c_{m}=1,\overset{―}{t_{S}}=0.1$.

*Dependence on input parameters.* Since in Eq (104) only $C=M_{\text{in}}c_{m}f_{\text{out}}/(N_{\text{in}}c)$ depends on *N*_in_ while *A* and *B* do not (see also Eqs (93)–(97)), the capacity is proportional to the input layer size *N*_in_ (Figs 5B, 14 bottom row). Further, we note that the optimal transition probability $\eta_{opt}$ in Eq (103) does not depend on the input layer size *N*_in_, but it does depend on the number of active input units $M_{\text{in}}=f_{\text{in}}N_{\text{in}}$ (Figs 5A, 14 top row).

We further observe that the maximal capacity as a function of the number of active input units *M*_in_ has a maximum that is independent of *N*_in_. In the range where $\eta_{\text{opt}}=eA/B$, the maximal capacity as a function of *M*_in_


$$\begin{array}{c} P_{\text{max}}^{*}(M_{\text{in}})=\frac{\ln(A(M_{\text{in}}))-\ln(B(M_{\text{in}}))-\ln\left(\frac{eA(M_{\text{in}})}{B(M_{\text{in}})}\right)}{-\frac{eA(M_{\text{in}})}{B(M_{\text{in}})}C(M_{\text{in}})}=\frac{B(M_{\text{in}})}{eA(M_{\text{in}})C(M_{\text{in}})} \end{array}$$ (105)

is strictly monotonically decreasing: For $M_{1}<M_{2}$, we have


$$\begin{array}{cc} & P_{\text{max}}^{*}(M_{1})>P_{\text{max}}^{*}(M_{2})\Leftrightarrow \frac{B(M_{1})}{eA(M_{1})C(M_{1})}>\frac{B(M_{2})}{eA(M_{2})C(M_{2})} \end{array}$$ (106)

$$\begin{array}{cc} & ⇐\frac{B(M_{1})}{A(M_{1})C(M_{1})}>\frac{B(M_{2})}{A(M_{1})C(M_{2})}\Leftrightarrow \frac{B(M_{1})}{C(M_{1})}>\frac{B(M_{2})}{C(M_{2})} \end{array}$$ (107)

$$\begin{array}{cc} & \Leftrightarrow \frac{M_{1}+2R_{g}^{2}}{M_{1}}>\frac{M_{2}+2R_{g}^{2}}{M_{2}}\Leftrightarrow M_{2}>M_{1}, \end{array}$$ (108)

where we used that $A(M_{1})<A(M_{2})$ because *A* is monotonically increasing as a function of *M*_in_ (due to $R_{s}>0$ for *f*_out_<0.5) and that *A* has to be positive in order to yield a real valued number for the capacity in Eq (92). Thus, if there is a maximum of $P_{\text{max}}^{*}(M_{\text{in}})$, it must occur within the range of *M*_in_ values that have an optimal transition probability of 1 (see Fig 14A,14B,14C). In order to find this maximum, we set η=1 in Eq (98), and then we set the derivative (with respect to *M*_in_) of the maximal capacity


$$\begin{array}{c} P_{\text{max}}^{*}(M_{\text{in}})=\frac{\ln(A(M_{\text{in}}))-\ln(B(M_{\text{in}}))}{-C(M_{\text{in}})} \end{array}$$ (109)

to zero:


$$\begin{array}{c} {P_{\text{max}}^{*}}^{\prime }(M_{\text{in}})\overset{!}{=}0. \end{array}$$ (110)

This equation can be solved numerically for fixed values of $c,c_{m},f_{\text{out}}$, and $\overset{―}{t_{S}}$. For example, for $c=0.2,c_{m}=1,f_{\text{out}}=0.006$ and $\overset{―}{t_{S}}=0.1$, we obtain an optimal number of active input units *M*_in_ = 13, which yields a maximal capacity $P_{max}(13)\approx 1.11\cdot N_{\text{in}}$ (see Fig 14C).

The fit between the theoretical approximation and the actual capacity obtained in numerical simulations improves for increasing input layer size *N*_in_, increasing input activation ratio *f*_in_, and increasing output activation ratio *f*_out_ (see comparison in Table 2). As discussed before, we need $M_{\text{in}}\cdot c\gg 1$ and $M_{\text{in}}\cdot (1-\rho_{g}(u_{mod}))\gg 1$ in order to approximate the distributions of the dendritic sums by Gaussian distributions. A large *f*_out_ reduces the multimodality of the genuine distribution and hence also allows for a better approximation (see Discussion in S4 Appendix).

**Table 2:** Deviation between theory and simulations: Maximal capacity obtained from theory divided by maximal capacity obtained from simulations for various values of *N*_in_ and *f*_out_ averaged across all *M*_in_ values shown in Fig 14 except the two smallest *M*_in_ = 3 and *M*_in_ = 6 (which do not fulfill the condition $M_{in}\gg 1$ and for which the fit is thus not accurate enough). Deviation calculated based on Eq (92) (corresponding to dashed lines in Fig 14). In brackets: Deviation calculated based on (98), i.e., with the Taylor approximation (95) (corresponding to solid lines in Fig 14).

	*f*_out_ = 0.1	*f*_out_ = 0.025	*f*_out_ = 0.006
*N*_in_ = 1000	1.21 (1.36)	1.34 (1.53)	1.44 (1.67)
*N*_in_ = 2000	1.13 (1.21)	1.22 (1.32)	1.33 (1.45)
*N*_in_ = 4000	1.11 (1.16)	1.16 (1.22)	1.22 (1.28)
*N*_in_ = 8000	1.06 (1.09)	1.11 (1.14)	1.24 (1.18)

#### Extension to noisy input patterns during retrieval.

An extension of the derivation of the memory capacity to the case of noisy input patterns during retrieval with noise level ε∈(0,1) (see the earlier section ‘Noisy input patterns during retrieval’ for details on the distributions of dendritic sums with noisy cues) yields the following capacity (see S4 Appendix for details on the derivation):


$$\begin{array}{c} P_{\varepsilon }^{*}=\left\lfloor \frac{\ln\left(\frac{A_{\varepsilon }}{\eta B_{\varepsilon }}\right)}{\ln\left(1-\frac{f_{\text{in}}\eta c_{m}}{c}\right)f_{\text{out}}}+\frac{1}{2}\right\rfloor \approx \frac{c}{-f_{\text{in}}f_{\text{out}}\eta c_{m}}\ln\left(\frac{A_{\varepsilon }}{\eta B_{\varepsilon }}\right) \end{array}$$ (111)

with


$$A_{\varepsilon }≔\left((1-2c)R_{g}^{2}+\sqrt{2M_{\text{in}}c(1-c)}R_{s}\right)\left(1-\frac{\varepsilon }{1-f_{\text{in}}}\right)+|R_{g}|\;\cdot \sqrt{\left(2\sqrt{2M_{\text{in}}c(1-c)}(1-2c)R_{s}+{\left(1-2c\right)}^{2}R_{g}^{2}+2c(1-c)M_{\text{in}}\right)\;\cdot {\left(1-\frac{\varepsilon }{1-f_{\text{in}}}\right)}^{2}-4c(1-c)(R_{s}^{2}-R_{g}^{2})\left(1-\frac{\varepsilon (1-2f_{\text{in}})}{{\left(1-f_{\text{in}}\right)}^{2}}\right)}$$ (112)

and


$$\begin{array}{c} B_{\varepsilon }:=(c_{m}-c)\left[M_{\text{in}}{\left(1-\frac{\varepsilon }{1-f_{\text{in}}}\right)}^{2}+2R_{g}^{2}\left(1-\frac{\varepsilon (1-2f_{\text{in}})}{(1-f_{\text{in}}{)}^{2}}\right)\right]. \end{array}$$ (113)

The optimal transition probability $\eta_{\text{opt}}$ and hence the maximal capacity $P_{\text{max}}^{*}$ of the network can be calculated in the same way as without noise (compare to Section ‘Optimal transition probability and maximal capacity’). The only difference lies in the expressions $A_{\varepsilon }$ and $B_{\varepsilon }$ that were previously called *A* and *B*. Expression $C=M_{\text{in}}c_{m}f_{\text{out}}/(N_{\text{in}}c)$ remains the same as in Eq (97). We have


$$\begin{array}{c} \eta_{\text{opt},\varepsilon }\approx min\left(\frac{eA_{\varepsilon }}{B_{\varepsilon }},1\right) \end{array}$$ (114)

and


$$\begin{array}{c} P_{\text{max},\varepsilon }^{*}\approx \frac{\ln(A_{\varepsilon })-\ln(B_{\varepsilon })-\ln(\eta_{\text{opt},\varepsilon })}{-\eta_{\text{opt},\varepsilon }C}, \end{array}$$ (115)

as also stated in Eqs (24) and (27) in the Results (Section ‘Noisy input patterns during retrieval’). In the range where $\eta_{\text{opt},\varepsilon }<1$, we thus can express the maximal capacity as


$$\begin{array}{c} P_{\text{max},\varepsilon }^{*}=\frac{B_{\varepsilon }}{eA_{\varepsilon }C}. \end{array}$$ (116)

We can assume that $P_{\text{max}}^{*}>P_{\text{max},\varepsilon }^{*}$, where $P_{\text{max}}^{*}$ is the maximal capacity for ε=0 defined in Eq (98), because the additional noise on the input patterns during retrieval naturally has a detrimental effect on the capacity. In the range of parameters where $\eta_{\text{opt}}<1$ (see Eq (105)) and $\eta_{\text{opt},\varepsilon }<1$, we thus have


$$\begin{array}{c} P_{\text{max}}^{*}>P_{\text{max},\varepsilon }^{*}\Leftrightarrow \frac{B}{eAC}>\frac{B_{\varepsilon }}{eA_{\varepsilon }C}\Leftrightarrow \frac{eA}{B}<\frac{eA_{\varepsilon }}{B_{\varepsilon }}\Leftrightarrow \eta_{\text{opt}}<\eta_{\text{opt},\varepsilon }, \end{array}$$ (117)

since *C* > 0 and we can assume *A*/*B* > 0 and $A_{\varepsilon }/B_{\varepsilon }>0$ in order to obtain a real-valued capacity.

The optimal transition probability with noise during retrieval $\eta_{\text{opt},\varepsilon }$ can be further approximated (details in S4 Appendix) as a multiple of the optimal transition probability without noise $\eta_{\text{opt}}$ by


$$\begin{array}{c} \eta_{\text{opt},\varepsilon }\approx \eta_{\text{opt},\varepsilon }^{\text{approx}}=\min\left(\frac{eA}{\left(1-\frac{\varepsilon }{1-f_{\text{in}}}\right)B},1\right)=\frac{\eta_{\text{opt}}}{\left(1-\frac{\varepsilon }{1-f_{\text{in}}}\right)} \end{array}$$ (118)

and the maximal capacity $P_{\text{max},\varepsilon }^{*}$ by


$$\begin{array}{c} P_{\text{max},\varepsilon }^{*}\approx P^{*}{|}_{\eta =\eta_{\text{opt},\varepsilon }}+\frac{\ln\left(1-\frac{\varepsilon }{1-f_{\text{in}}}\right)c}{f_{\text{in}}f_{\text{out}}\eta_{\text{opt},\varepsilon }c_{m}}. \end{array}$$ (119)

For ε≪1 and $f_{\text{in}}\ll 1$, we thus obtain the approximation


$$\begin{array}{c} P_{\text{max},\varepsilon }^{*}\approx P^{*}{|}_{\eta =\eta_{\text{opt},\varepsilon }}-\frac{\varepsilon c}{f_{\text{in}}f_{\text{out}}\eta_{\text{opt},\varepsilon }c_{m}}. \end{array}$$ (120)

A comparison of $P_{\text{max}}^{*}$ and $P_{\text{max},\varepsilon }^{*}$ and its approximation given in Eq (118) as well as of $\eta_{\text{opt}}$ and $\eta_{\text{opt},\varepsilon }$ and its approximation given in Eq (120) is shown in Fig 6D,6E (also see S4 Fig).

### Network simulations

In this section, we describe the network simulations performed to obtain the numerical results presented in this paper. First, we report how the main parameters were sampled. Then, we explain the simulation paradigm in detail. Last, we clarify how we average across results for different patterns before briefly broaching the issue of enforcing the output activation ratio.

All simulations were performed in python 3.9. All code written in support of this publication will be made publicly available at https://itbgit.biologie.hu-berlin.de/auer/sparseness_plasticity_lifetime before publication.

#### Parameter sampling.

Unless stated otherwise, we perform all simulations for transition probabilities


$$\begin{array}{c} \eta \in \{1,0.9,0.8,0.7,0.6,0.5,0.4,0.3,0.2,0.1\} \end{array}$$ (121)

and define the value for which the capacity is the largest as the optimal transition probability. For the reference network size of $N_{\text{in}}=N_{\text{out}}=1000$, the activation ratios are typically sampled as


$$\begin{array}{c} f_{\text{in}},f_{\text{out}}\in \{0.2,0.1,0.05,0.025,0.012,0.006,0.003\}. \end{array}$$ (122)

For larger network sizes, smaller values *f*_in_ and *f*_out_ are added to achieve minimal *M*_in_ and *M*_out_ values comparable to those of the small reference network.

#### The simulation paradigm.

We now describe how patterns are sampled and how the weight matrix is initialized as well as how a simulation step is executed and how many steps are carried out in total. As also explained in the Results, Section ‘Network model, learning paradigm, and quantification of capacity’, we aim at quantifying the memory capacity of the network by tracking the signal quality, which depends on the Hamming distance between a calculated output vector and the corresponding target output vector. The capacity is defined as the number of subsequently learned patterns for which the signal quality reaches a specific value. In each simulation step the association between one pattern pair is learned, and in order to calculate the capacity we need a number of simulation steps that is larger than the capacity itself.

*Input and output patterns.* Input patterns are generated as vectors **x** of length *N*_in_ by randomly choosing *M*_in_ entries to be one while the others are zero. Analogously, output patterns are random vectors **y** of length *N*_out_ with *M*_out_ entries equal to one. One random input pattern **x** together with one random output pattern **y** constitute a pattern pair (𝐱,𝐲). Note that we do not explicitly exclude the possibility of generating the same vector twice as an input or as an output pattern, but for $M_{\text{in}},M_{\text{out}}\gg 1$ and $N_{\text{in}}\gg M_{\text{in}}$, $N_{\text{out}}\gg M_{\text{out}}$, this is very unlikely.

If noisy input patterns are used as retrieval cues, they are generated based on the noise-less input patterns in the following way: Each originally active input unit is deactivated with probability ε. We count the number of deactivated units and randomly choose the same number of originally inactive input units to be activated. The number of active input units *M*_in_ is thus maintained.

*Initialization of the weight matrix.* First, we generate a matrix $J_{m}\in \{0,1{\}}^{N_{\text{out}}\times N_{\text{in}}}$ that represents the morphological connections with connectivity $c_{m}$. In every row of $J_{m}$, we set $c_{m}N_{\text{in}}$ randomly chosen entries to one, and the other entries are zero. The binary matrix $J_{m}$ acts as a mask on the actual weight matrix *J* in every update step such that only the entries in *J* that correspond to a one in $J_{m}$ can be altered. The weight matrix *J* of size $N_{\text{out}}\times N_{\text{in}}$ is initialized such that per row *cN*_in_ entries of those that are morphologically available (i.e., have a value of one in $J_{m}$) are set to one, the rest is set to zero.

*One simulation step.* Each simulation step *k* corresponds to one pattern pair $\left({𝐱}^{\left[k\right]},{𝐲}^{\left[k\right]}\right)$ being learned. In one simulation step *k*, the weights of the matrix in the previous step, $J^{\left[k-1\right]}$, are first updated according to Hebbian learning and then updated according to the homeostatic mechanism (described in the Results, Section ‘Network model, learning paradigm, and quantification of capacity’). This corresponds to transforming $J^{\left[k-1\right]}$ into *J*^[*k*]^.

Then, for a fixed *J*^[*k*]^, we evaluate the output of the network for all input patterns of pairs 0,…,k that have been learned up to this point; the input patterns are presented to the network again one after the other with plasticity off, and the corresponding output patterns are calculated. By ${\hat{𝐲}}^{\left[i\right]}(j)$ we denote the output of the *i*-th input pattern calculated after learning *j* subsequent pattern pairs. To summarize, the outputs


$$\begin{array}{c} {\hat{𝐲}}^{\left[0\right]},\ldots ,{\hat{𝐲}}^{\left[k\right]} \end{array}$$ (123)

for each of the input patterns


$$\begin{array}{c} {𝐱}^{\left[0\right]},\ldots ,{𝐱}^{\left[k\right]} \end{array}$$ (124)

are calculated and compared to the original target outputs


$$\begin{array}{c} {𝐲}^{\left[0\right]},\ldots ,{𝐲}^{\left[k\right]}, \end{array}$$ (125)

respectively. The *k* + 1 Hamming distances


$$\begin{array}{c} H\left({𝐲}^{\left[0\right]},{\hat{𝐲}}^{\left[0\right]}(k)\right),H\left({𝐲}^{\left[1\right]},{\hat{𝐲}}^{\left[1\right]}(k-1)\right),\ldots ,H\left({𝐲}^{\left[k-1\right]},{\hat{𝐲}}^{\left[k-1\right]}(1)\right),H\left({𝐲}^{\left[k\right]},{\hat{𝐲}}^{\left[k\right]}(0)\right) \end{array}$$ (126)

between target and calculated output are stored. Note that these Hamming distances that are stored after one particular simulation step belong to different numbers of additional pattern pairs that are stored after the pattern that is tested. With respect to pattern 0, the maximum number *k* of additional pattern pairs have been stored, while with respect to pattern *k*, no additional pattern pair has been stored.

*Number of simulation steps*. The average Hamming distance between two random *f*_out_-sparse vectors of length *N*_out_ is


$$\begin{array}{c} H_{avg}=2N_{\text{out}}f_{\text{out}}(1-f_{\text{out}})\;. \end{array}$$ (127)

To define a threshold $T_{S}$ for the successful retrieval of an output pattern, we use a certain fraction of $H_{avg}$,


$$\begin{array}{c} T_{S}=t_{S}H_{avg}\;, \end{array}$$ (128)

where $t_{S}\in (0,1)$. If not stated otherwise, the retrieval threshold is chosen as


$$\begin{array}{c} T_{S}=N_{\text{out}}f_{\text{out}}(1-f_{\text{out}}) \end{array}$$ (129)

and hence, $t_{S}=0.5$.

The degradation of the calculated output vector is quantified by means of the signal quality *S*, which is defined as the difference between the average Hamming distance of two random vectors, $H_{avg}$, and $H(𝐲,\hat{𝐲})$:


$$\begin{array}{c} S:=H_{avg}-H(𝐲,\hat{𝐲})\;. \end{array}$$ (130)

For a successful retrieval of the calculated output, we then require $S>t_{S}H_{avg}$. This translates to the assumption that an output pattern can be recovered if the Hamming distance $H(𝐲,\hat{𝐲})$ between the target output **y** and the calculated output $\hat{𝐲}$ is smaller than the threshold $(1-t_{S})H_{\text{avg}}$. In this case, the output, which is typically degraded by subsequent storage of other input-output pairs, is still close enough to the original output pattern. To be more specific, let us consider the example of input-output pattern pair 0 and *k* subsequent learning steps. The signal quality is then


$$\begin{array}{c} S_{k}:=H_{avg}-H\left({𝐲}^{\left[0\right]},{\hat{𝐲}}^{\left[0\right]}(k)\right), \end{array}$$ (131)

which is based on the Hamming distance $H\left({𝐲}^{\left[0\right]},{\hat{𝐲}}^{\left[0\right]}(k)\right)$ of the target output ${𝐲}^{\left[0\right]}$ of the very first pattern pair and its output ${\hat{𝐲}}^{\left[0\right]}(k)$ calculated with weight matrix *J*^[*k*]^.

The maximum number of simulation steps for a particular parameter combination was determined by the observed degradation of calculated output patterns. Once the signal quality for the first time is less than $0.4\cdot H_{\text{avg}}$ (or equivalently, when the Hamming distance is above $0.6\cdot H_{\text{avg}}$), 200 additional patterns are learned before the simulation is stopped.

We denote the total number of patterns that have been learned by the network at the end of a simulation by *K*.

#### Averaging.

The signal quality that is shown in various figures in this manuscript is always averaged across many patterns. Recall that the number of subsequently learned patterns *P* is understood as relative to the pattern number k∈{0,…,K}. For each pattern $\left({𝐱}^{\left[k\right]},{𝐲}^{\left[k\right]}\right)$ that the network learns, we compute the Hamming distance of the target output and the calculated output after P=0,1,2,… patterns additionally learned after the *k*-th pattern, hence with weight matrix *J*^[*k*+*P*]^. For a fixed *P* (always relative to a particular pattern), we average across all patterns for which we have learned at least *P* subsequent patterns:


$$\begin{array}{c} H_{K}\left(𝐲,\hat{𝐲}(P)\right):=\frac{1}{K-P}\sum_{k=0}^{K-P}H\left({𝐲}^{\left[k\right]},{\hat{𝐲}}^{\left[k\right]}(P)\right). \end{array}$$ (132)

All simulation data shown in this manuscript are obtained by averaging across at least *N*_avg_ = 200 patterns. This means, if the largest *P* shown in a figure is $N_{P}$, simulations ran for at least $K=N_{P}+N_{\text{avg}}$ steps.

Whenever we investigate the distributions of dendritic sums for a particular number of subsequent patterns *P*, two kinds of averaging are done: First, we pool the dendritic sums of all genuine output units and of all spurious output units of one pattern after learning *P* subsequent patterns, respectively. In addition, we pool a certain number of patterns. A comparison of distributions of dendritic sums obtained from numerical simulations and from theory (Eqs (44) and (46)) is shown in S5 Fig.

#### A note on the output sparseness.

During retrieval, the activation of the output units is calculated as


$$\begin{array}{c} {\hat{y}}_{j}^{\left[k\right]}(P)=\Theta \left(\left(\sum_{i=1}^{N_{\text{in}}}J_{ji}^{\left[k+P\right]}x_{i}^{\left[k\right]}\right)-T_{\text{in}}^{\left[P\right]}\right) \end{array}$$ (133)

where Θ is the Heaviside step function. Note that, due to the discrete nature of the dendritic sums of the output units, it is unlikely that an activation threshold $T_{\text{in}}^{\left[P\right]}$ exists that allows to activate exactly *M*_out_ output units. Most of the times, there is a group of units with identical dendritic sums that are exactly at the critical value. If the threshold is chosen larger than these dendritic sums, too few units are activated, if it is chosen smaller, too many units are activated. Since we want to keep *M*_out_ fixed, we have to find a way to enforce the exact output sparseness. Hence, we typically randomly choose the right number of these units to be activated and the rest to be inactive. On average, this has a slightly deteriorative effect on the capacity of the network because spurious units might be chosen to be activated even though some genuine units have the same dendritic sum. In S7 Appendix, we discuss in more detail for which parameters this might have significant effects on the capacity of the network and how the enforcement of the output sparseness level could be handled differently in order to avoid this problem.

## Supporting information

S1 FigComparison of optimal transition probability $\eta_{opt}$ and maximal capacity $P_{max}^{*}$ for different retrieval thresholds $T_{S}$.(PDF)

S2 FigComparison of optimal transition probability $\eta_{opt}$ and maximal capacity $P_{max}^{*}$ for different morphological connectivity levels $c_{m}$.(PDF)

S3 FigComparison of optimal transition probability $\eta_{opt}$ and maximal capacity $P_{max}^{*}$ for different functional connectivity levels *c.*(PDF)

S4 FigComparison of optimal transition probability $\eta_{opt,\varepsilon }$ and maximal capacity $P_{max,\varepsilon }^{*}$ for different input noise levels ε during retrieval.(PDF)

S5 Fig Comparison of distributions of dendritic sums obtained from network simulations and from theory. (PDF)

S6 FigDistributions of dendritic sums with noise (ε=0.4).(PDF)

S1 Appendix Probabilities of functional connections. Derivation of probabilities of functional connections used in the distributions of dendritic sums.
(PDF)

S2 Appendix Analysis of shape of distributions. Discussion of the multimodality of the distributions of dendritic sums.
(PDF)

S3 Appendix Solution of the Signal Quality Equation. Numerical and analytical calculation of the signal quality as a function of *P*, including an analytical approximation of the signal quality with noisy input patterns.
(PDF)

S4 Appendix Solution of the Capacity Equation. Details regarding the derivation of the memory capacity for noise-less and noisy input patterns.
(PDF)

S5 Appendix Monotonicity of capacity for small *f*_in_. Showing monotonic increase of capacity for input activation ratios close to zero.
(PDF)

S6 Appendix Comparison to other versions of homeostasis. Optimal transition probability and maximal capacity for fixed number of functional connections per input neuron and fixed number of functional connections averaged across the whole network.
(PDF)

S7 Appendix Can the output activation ratio be enforced? Derivation of an upper bound for the error in activating the right number of output units and discussion of practical solutions for when the exact output activation ratio cannot be achieved.
(PDF)

S8 Appendix Information-theoretic synaptic capacity. Translation of main results from pattern capacity to an information-theoretic measure of synaptic capacity.
(PDF)
