# Supplementary material for: Population sparseness determines strength of Hebbian plasticity for maximal memory lifetime in associative networks
Source: PLoS Comput Biol. 2026 Jul 6;22(7):e1013235. doi: 10.1371/journal.pcbi.1013235 (PMC13390959; doi:10.1371/journal.pcbi.1013235)
Supplement: S1 Fig — (PDF) [file pcbi.1013235.s001.pdf]

S1 Figure

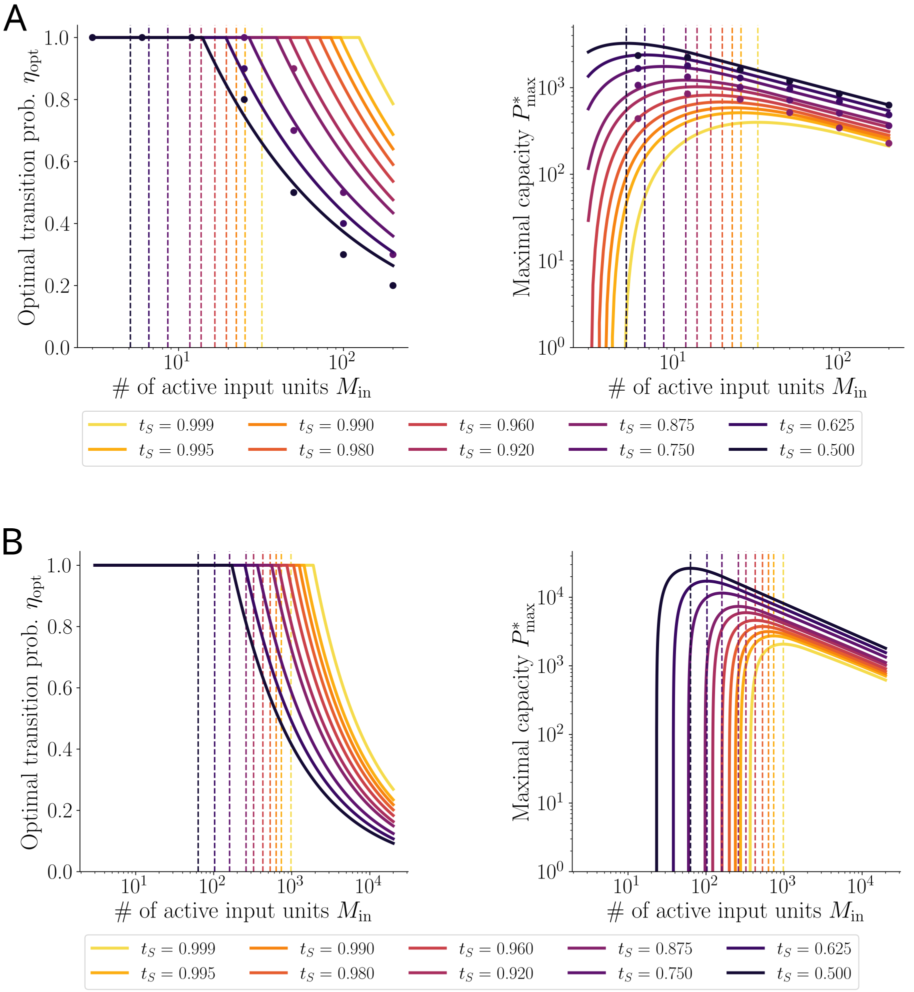

Fig S1. Comparison of optimal transition probability $\eta_{\mathbf{opt}}$ and maximal capacity $P^*_{\mathbf{max}}$ for different retrieval thresholds $T_S = t_S H_{\mathbf{avg}}$.
Left: The optimal transition probability $\eta_{\rm opt}$ increases with increasing t_S (color coded). Right: The maximal capacity decreases with increasing t_S. The number of active input units $M_{\rm in}$ that yields the largest capacity increases with increasing retrieval ratio t_S (vertical dashed lines). Solid curves show theoretical results obtained from Eq (22) and Eq (23), and dots show numerical results. (**A**) Morphological connectivity $c_m = 1$, functional connectivity $c = 0.2$. (**B**) $c_m = 0.1$, $c = 0.02$. Further parameter values: $N_{\rm in} = N_{\rm out} = 1000$, $f_{\rm out} = 0.006$, $N_{\rm avg} = 200$.
