# Supplementary material for: Population sparseness determines strength of Hebbian plasticity for maximal memory lifetime in associative networks
Source: PLoS Comput Biol. 2026 Jul 6;22(7):e1013235. doi: 10.1371/journal.pcbi.1013235 (PMC13390959; doi:10.1371/journal.pcbi.1013235)
Supplement: S2 Fig — (PDF) [file pcbi.1013235.s002.pdf]

S2 Figure

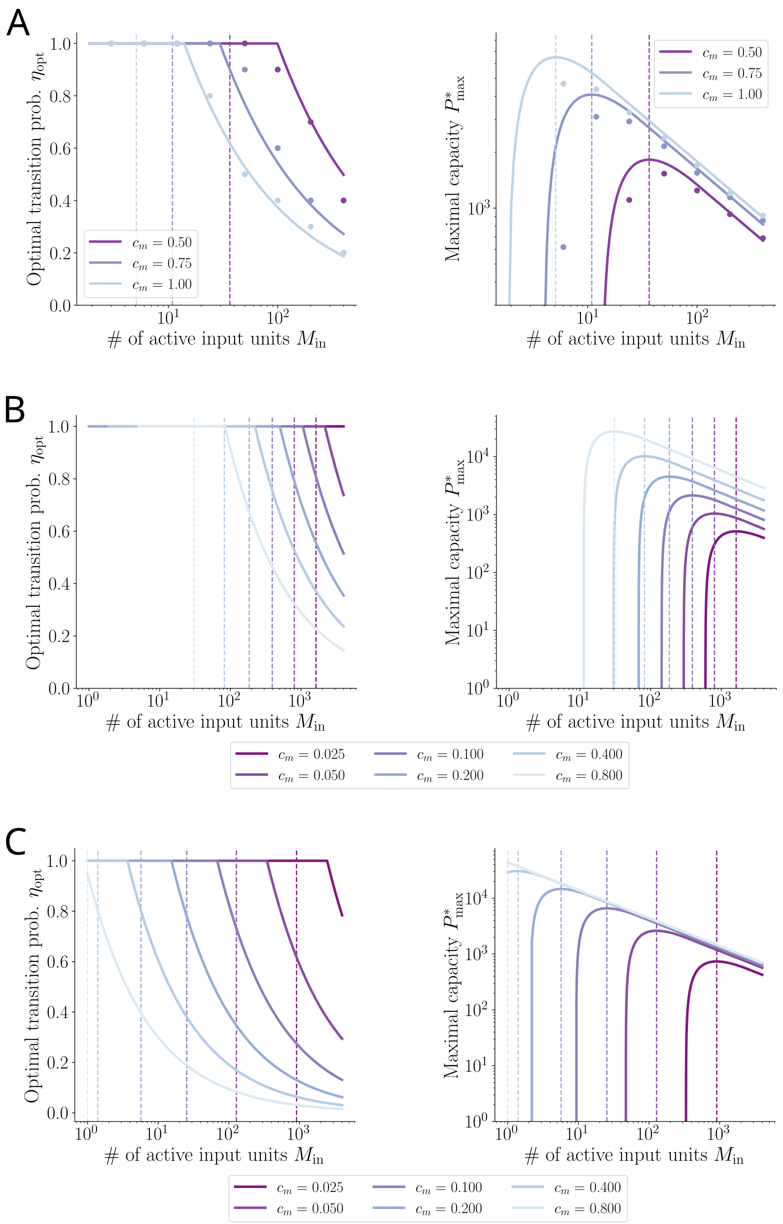

Fig S2. Comparison of optimal transition probability η_{opt} and maximal capacity $P^*_{\max}$ for different morphological connectivity levels c_m.
Left: The optimal transition probability η_{opt} decreases with increasing c_m (color coded). Right: The maximal capacity increases with increasing c_m. The number of active input units M_{in} that yields the largest capacity decreases with increasing morphological connectivity c_m (vertical dashed lines). Solid curves show theoretical results obtained from Eq (22) and Eq (23), and dots show numerical results. (**A**) Comparison between theory and simulations. $N_{\mathrm{in}} = N_{\mathrm{out}} = 2000$, $f_{\mathrm{out}} = 0.006$, $c = 0.2$, $t_S = 0.5$, $N_{\mathrm{avg}} = 200$. (**B**) Different values of c_m with fixed c. $N_{\mathrm{in}} = N_{\mathrm{out}} = 20000$, $f_{\mathrm{out}} = 0.006$, $c = 0.01$, $t_S = 0.5$. (**C**) Different values of c_m with $c = c_m/2$. $N_{\mathrm{in}} = N_{\mathrm{out}} = 2000$, $f_{\mathrm{out}} = 0.006$, $c = c_m/2$, $t_S = 0.5$.
