# Supplementary material for: Population sparseness determines strength of Hebbian plasticity for maximal memory lifetime in associative networks
Source: PLoS Comput Biol. 2026 Jul 6;22(7):e1013235. doi: 10.1371/journal.pcbi.1013235 (PMC13390959; doi:10.1371/journal.pcbi.1013235)
Supplement: S3 Fig — (PDF) [file pcbi.1013235.s003.pdf]

S3 Figure

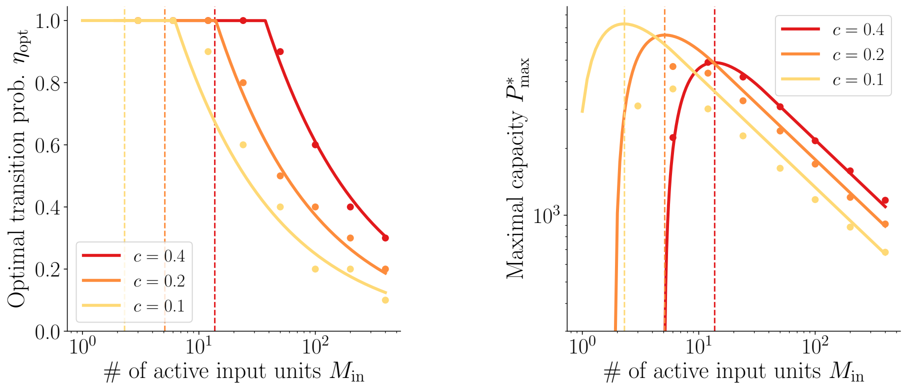

Fig S3. Comparison of optimal transition probability $\eta_{\mathbf{opt}}$ and maximal capacity $P^*_{\mathbf{max}}$ for different functional connectivity levels c.
Left: The optimal transition probability η_{opt} increases with increasing c. Right: The maximal capacity increases with increasing c for large enough M_{in}. The number of active input units M_{in} that yields the largest capacity increases with increasing functional connectivity c (vertical dashed lines). Solid lines show theoretical results obtained from Eq (22) and Eq (23), and dots show numerical results. For small c, the analytical approximation strongly overestimates the maximal capacity, especially if only a small number of input units is active because the approximation of the binomial distributions by normal distributions is unsatisfactory for small $M_{\text{in}}c$. Further parameter values: $N_{\text{in}} = N_{\text{out}} = 2000, f_{\text{out}} = 0.006, c_m = 1, t_S = 0.5, N_{\text{avg}} = 200$.
