# Supplementary material for: Population sparseness determines strength of Hebbian plasticity for maximal memory lifetime in associative networks
Source: PLoS Comput Biol. 2026 Jul 6;22(7):e1013235. doi: 10.1371/journal.pcbi.1013235 (PMC13390959; doi:10.1371/journal.pcbi.1013235)
Supplement: S4 Fig — (PDF) [file pcbi.1013235.s004.pdf]

S4 Figure

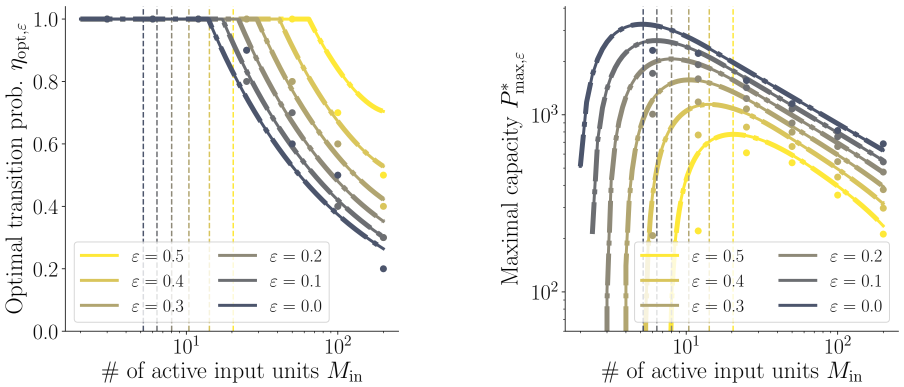

Fig S4. Comparison of optimal transition probability η_{opt} and maximal capacity P^*_{max} for different input noise levels ε during retrieval.
Left: The optimal transition probability $\eta_{\text{opt},\varepsilon}$ increases with increasing ε. Right: The maximal capacity decreases with increasing ε. The number of active input units M_{in} that yields the largest capacity increases with increasing noise level ε (vertical dashed lines). Solid lines show theoretical results obtained from Eq (24) and Eq (27), dash-dotted lines show an approximation for small ε (Eqs (26) and (28)) and dots show numerical results. Further parameter values: $N_{\text{in}} = N_{\text{out}} = 1000$, $f_{\text{out}} = 0.006$, $c = 0.2$, $c_m = 1$, $t_S = 0.5$, $N_{\text{avg}} = 200$.
