# Supplementary material for: Population sparseness determines strength of Hebbian plasticity for maximal memory lifetime in associative networks
Source: PLoS Comput Biol. 2026 Jul 6;22(7):e1013235. doi: 10.1371/journal.pcbi.1013235 (PMC13390959; doi:10.1371/journal.pcbi.1013235)
Supplement: S5 Fig — (PDF) [file pcbi.1013235.s005.pdf]

S5 Figure

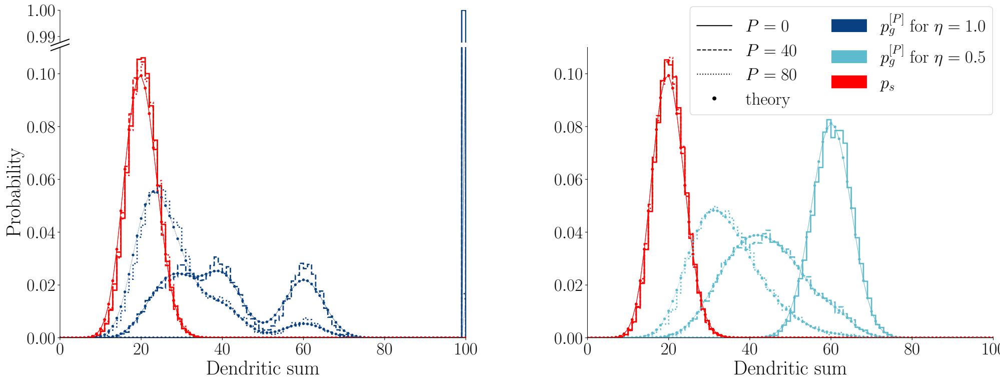

Fig S5. Comparison of distributions of dendritic sums obtained from network simulations and from theory.
Histograms show distributions of dendritic sums estimated from numerical simulations, pooled across 200 genuine units and 1800 spurious units and 50 patterns. Different line styles represent different numbers of subsequent patterns ($P = 0$ solid lines, $P = 40$ dashed lines, $P = 80$ dotted lines). Theoretical approximations of the distributions are shown as dots (connected by lines in the respective line styles). Left: $\eta = 1$, right: $\eta = 0.5$. Further parameters: $N_{\text{in}} = 1000, N_{\text{out}} = 2000, f_{\text{in}} = f_{\text{out}} = 0.1, c = 0.2, c_m = 1$.
