# Supplementary material for: Population sparseness determines strength of Hebbian plasticity for maximal memory lifetime in associative networks
Source: PLoS Comput Biol. 2026 Jul 6;22(7):e1013235. doi: 10.1371/journal.pcbi.1013235 (PMC13390959; doi:10.1371/journal.pcbi.1013235)
Supplement: S6 Fig — (PDF) [file pcbi.1013235.s006.pdf]

S6 Figure

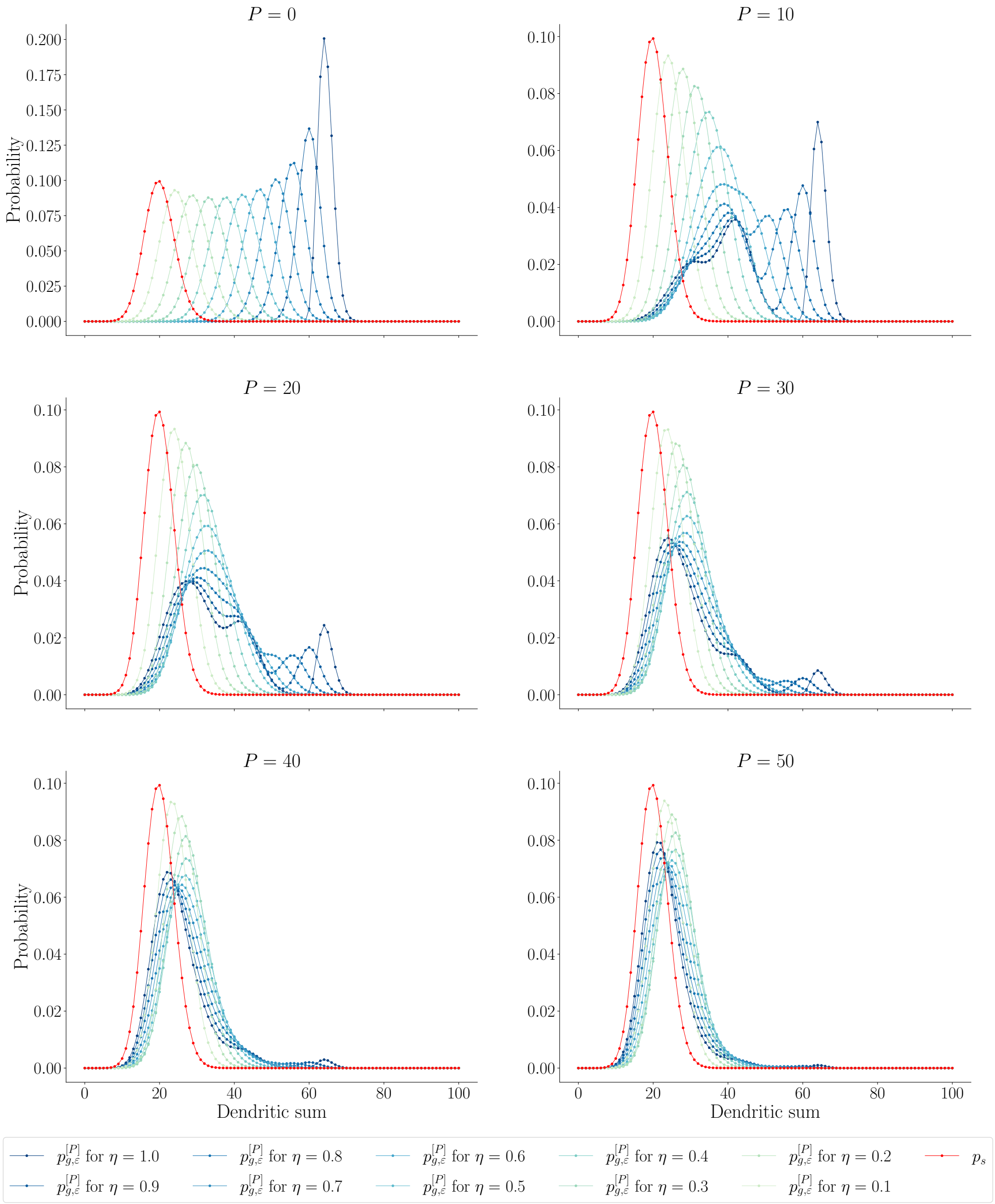

Fig S6. Distributions of dendritic sums with noise ($\varepsilon = 0.4$).
Same as Fig 8, in which we used $\varepsilon = 0.2$. Analytical probability mass functions of the distributions of dendritic sums with noise on the input patterns during retrieval after storing $P = 0, 10, 20, 30, 40, 50$ patterns. Spurious units in red; genuine units in blues, for several transition probabilities η. Other parameters: $N_{\text{in}} = 1000$, $f_{\text{in}} = f_{\text{out}} = 0.1$, $c = 0.2$, $c_m = 1$.
