# Supplementary material for: Population sparseness determines strength of Hebbian plasticity for maximal memory lifetime in associative networks
Source: PLoS Comput Biol. 2026 Jul 6;22(7):e1013235. doi: 10.1371/journal.pcbi.1013235 (PMC13390959; doi:10.1371/journal.pcbi.1013235)
Supplement: S1 Appendix — Derivation of probabilities of functional connections used in the distributions of dendritic sums. (PDF) [file pcbi.1013235.s007.pdf]

S1 Appendix

Probabilities of functional connections

In this appendix, we provide detailed derivations of the probabilities of connections. These derivations extend earlier works on the distributions of dendritic sums in Willshaw-like networks, for example [1–5], by considering an explicit plasticity parameter η and a homeostatic constraint on the number of functional connections.

We distinguish spurious and genuine output units. We first consider the probability of a functional connection to a spurious output unit (ρ_s), and in this case we will show that it is irrelevant whether the input unit is spurious or genuine. In contrast, for a genuine output unit, we must distinguish connections from a genuine input unit (ρ_g) and a spurious input unit (ρ_n).

Probability ρ_s of a functional connection to a spurious unit

In this subsection, the probability for a connection to a spurious unit to be functional is derived in detail. The final result, $\rho_s = c$, can be found in Eq (S1.5).

To be more specific, we consider an output unit that is spurious for the k-th pattern. Due to the initialization of connections, i.e., turning on cN_{in} connections per output unit (blue part in Fig S1.1A) and the fact that storing the k-th pattern does not affect the connections to spurious units, we have $\rho_s(0) = c$ (orange part in Fig S1.1B). Hence, we do not need to distinguish between genuine-spurious and spurious-spurious connections in the k-th pattern (but we will have to do so in the next section where we discuss the probability of a functional connection to a genuine unit).

While storing additional patterns, connections targeting output units that are spurious with respect to the k-th pattern will be updated because they might not be spurious with respect to the subsequent patterns. If an output unit in a subsequent pattern is also spurious, connections to this output unit are unchanged, and it is obvious that the connectivity remains c. More involved is the case where an output unit in the other pattern is genuine. Then the connections to this output unit are changed due to Hebbian learning, but there is also a homeostasis mechanism. As shown in the rest of this section, in this case, the connectivity also remains c.

Let us explain this latter result in more detail: We assume that the spurious output unit that we are investigating is active for one additional pattern $k + \kappa$ with $\kappa \in \mathbb{N}^+$ fixed. The probability $\rho_s(1)$ of a connection to this unit to be functional after the update step can be decomposed using the law of total probability

$$\mathcal{P}(A) = \sum_{i \in I} \mathcal{P}(A|B_i)\mathcal{P}(B_i), \tag{S1.1}$$

where $\{B_i\}_{i \in I}$ is a partition of the sample space. As conditions B_i, we distinguish between the four possible combinations that arise from two initial states of the connection and two activity states of the input unit: either the connection is already functional before learning pattern $k + \kappa$ (with probability c, orange part in Fig S1.1B) or it is silent (but morphologically available) (with probability $c_m - c$, gray part in Fig S1.1B); furthermore, the originating input unit is either active (with probability f_{in}) or inactive (with probability $1 - f_{\text{in}}$) in pattern $k + \kappa$. These four conditions have the following probabilities:

(i) $\mathcal{P}($*'connection previously functional and input unit active'*$) = cf_{\text{in}}$

(ii) $\mathcal{P}($*'connection previously silent and input unit active'*$) = (c_m - c)f_{\text{in}}$

(iii) $\mathcal{P}($*'connection previously silent and input unit inactive'*$) = (c_m - c)(1 - f_{\text{in}})$

(iv) $\mathcal{P}($*'connection previously functional and input unit inactive'* $) = c(1 - f_{\text{in}})$

Recall that the output unit that we investigate is spurious with respect to the k-th pattern but it is active in pattern $k + \kappa$. For each of the four cases above, we derive the conditional probability of a connection becoming functional based on the update rule that consists of Hebbian and homeostatic learning:

(i) If the connection is already functional and the corresponding input unit is active, the connection remains functional:
$\mathcal{P}($*'connection functional'* $\mid$ *'connection previously functional and input unit active'* $) = 1$ (yielding the green frame on the far left of Fig S1.1C)

(ii) If the connection is silent and the corresponding input unit is active, the connection is made functional with transition probability η:
$\mathcal{P}($*'connection functional'* $\mid$ *'connection previously silent and input unit active'* $)$ $= \eta$ (yielding the green area in Fig S1.1C)

(iii) If the connection is silent and the input unit is inactive, the connection remains silent:
$\mathcal{P}($*'connection functional'* $\mid$ *'connection previously silent and input unit inactive'* $)$ $= 0$ (represented by the gray area on the right of Fig S1.1C)

(iv) If the connection is functional and the input unit is inactive, the connection could be silenced due to the homeostasis mechanism (shown by the red frame in Fig S1.1C) or it could remain functional. As derived in Eq (39) in Section 'Hebbian and homeostatic update rules from a probabilistic perspective' in the Methods, the probability that it is silenced is $\eta f_{\text{in}}(c_m - c)/\left(c(1 - f_{\text{in}})\right)$ and the probability that it remains functional is thus
$\mathcal{P}($*'connection functional'* $\mid$ *'connection previously functional and input unit inactive'* $) = 1 - \frac{\eta f_{\text{in}}(c_m - c)}{c(1 - f_{\text{in}})}$ (represented by the orange area without the red frame and without the green frame in Fig S1.1C).

In total, according to Eq (S1.1), the probability of the connection to be functional can be expressed as

$$\rho_s(1) = \mathcal{P}(\textit{`connection functional'}) \tag{S1.2}$$

$$= 1 \cdot c f_{\text{in}} + \eta \cdot (c_m - c) f_{\text{in}} + 0 \cdot (c_m - c)(1 - f_{\text{in}}) + \left(1 - \frac{\eta f_{\text{in}}(c_m - c)}{c(1 - f_{\text{in}})}\right) \cdot c(1 - f_{\text{in}}) \tag{S1.3}$$

$$= c \tag{S1.4}$$

which is represented by the purple parts in Fig S1.1C.

The preservation of $\rho_s(k)$ relies on the statistical identity of units in the distribution of patterns. Specifically, every additional pattern in which a particular spurious output unit is active manipulates the connections to the spurious output unit in the same way. It follows that

$$\rho_s(u) = c, \quad \text{for all } u \in \mathbb{N}. \tag{S1.5}$$

In the following, we denote the distribution of the dendritic sums of spurious units for any P as

$$p_s(x) := p_s^{[0]}(x). \tag{S1.6}$$

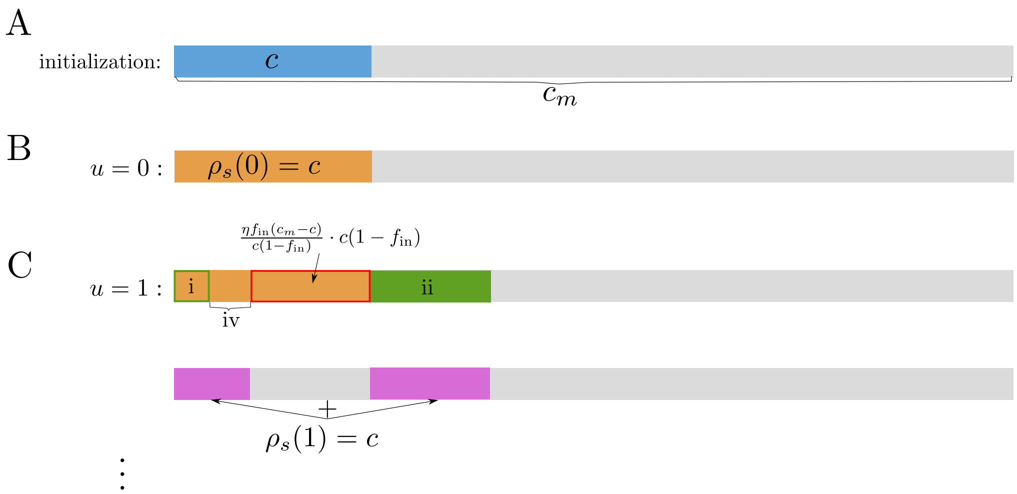

Fig S1.1. Illustration of the probability of a functional connection to a spurious unit.
Fractions of functional connections targeting a spurious output unit if the output unit is not active ($u = 0$) or active once ($u = 1$) across the pattern set. Gray parts represent fractions of silent connections. Colored parts represent fractions of functional connections. (**A**) Configuration after initialization. (**B**) Configuration for $u = 0$. (**C**) Configuration for $u = 1$.

Probability ρ_g of a functional connection to a genuine unit

We have seen that the probability ρ_s that a connection to a spurious unit is functional does not change with the output unit usage u. The probability $\rho_g(u)$ that a connection to a genuine unit is functional, however, strongly depends on u. In the following, we first derive $\rho_g(0)$ and $\rho_g(1)$ and then generalize this derivation recursively to $\rho_g(u)$ for $u > 1$. The final result can be found in Eq (S1.20).

Derivation of $\rho_g(0)$ and $\rho_g(1)$. We aim at calculating the probability $\rho_g(u)$ that a genuine-genuine connection of the k-th pattern (Fig S1.2 left, labeled by f_{in}) is functional under the condition that the respective output unit is active u times in the subsequent patterns $k+1, \ldots, k+P$. Here, we compute $\rho_g(u)$ for $u = 0, 1$. For large numbers of such connections, i.e., for $f_{\text{in}} N_{\text{in}} \cdot f_{\text{out}} N_{\text{out}} \gg 1$, the probabilities $\rho_g(0)$ and $\rho_g(1)$ can, by the law of large numbers, be interpreted as fractions.

Similarly to describing $\rho_g(u)$, we can describe the fraction of functional connections originating from input units that are inactive in the k-th pattern and targeting one specific genuine output unit, which are called spurious-genuine connections (Fig S1.2 right, labeled by $1 - f_{\text{in}}$). For the output unit being active never or once, we name these fractions $\rho_n(0)$ and $\rho_n(1)$, respectively. These probabilities of functional spurious-genuine connections are needed for deriving ρ_g, but they do not explicitly contribute to the dendritic sum of the output unit because they do not receive any input.

As discussed in the previous section, connections targeting a spurious output unit are not modified during learning the k-th pattern, and the probability of a functional connection to a spurious unit after learning the k-th pattern is the same as the probability at the initialization. Since there is no correlation between input patterns, the probability of functionality is identical for genuine-spurious and spurious-spurious connections. In contrast, the probability of a functional connection to a genuine output

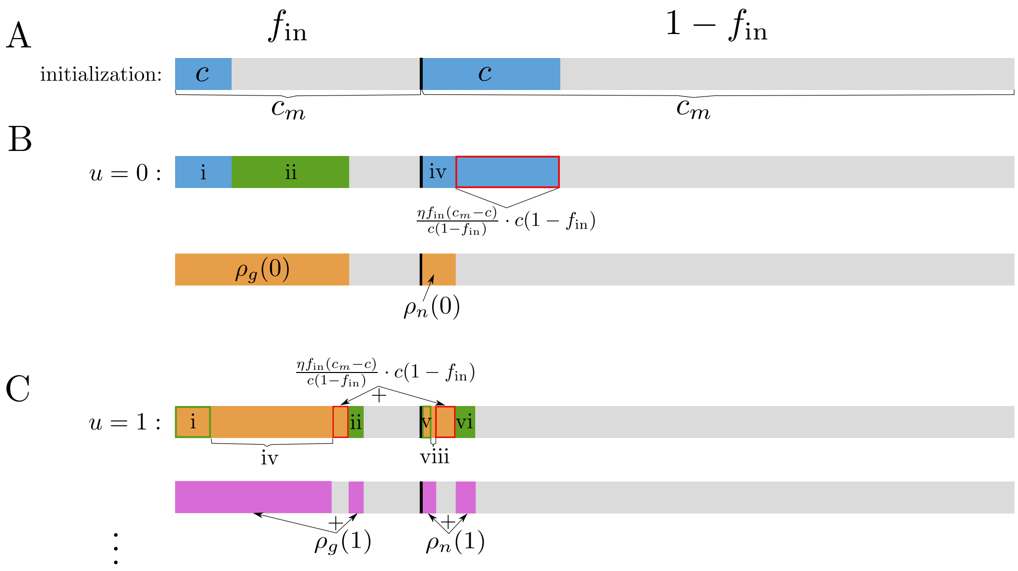

Fig S1.2. Illustration of the probability of a functional connection to a genuine unit.
Fractions of functional connections targeting a genuine output unit if the output unit is active u times across the pattern set. Left (below "f_{in}"): fraction f_{in} of connections that originate from an input unit that is active in the k-th pattern and will thus contribute to the dendritic sum of the output unit when the k-th input pattern is applied — the genuine-genuine connections; right (below "$1 - f_{\text{in}}$"): fraction $1 - f_{\text{in}}$ of connections that originate from an input unit that is inactive in the k-th pattern — the spurious-genuine connections. Gray areas represent fractions of silent connections. Colored areas represent fractions of functional connections. (**A**) Configuration after initialization. (**B**) Configuration for $u = 0$. (**C**) Configuration for $u = 1$.

unit after learning the k-th pattern (i.e., for $u = 0$) depends on whether the connection originates at an input unit that is active in the k-th pattern or not. Thus, we separately examine the probability of a functional genuine-genuine connection, ρ_g, and the probability of a spurious-genuine connection, ρ_n. Note that the two probabilities ρ_g and ρ_n, when weighted by the probabilities $\mathcal{P}(\text{'input unit active'}) = f_{\text{in}}$ and $\mathcal{P}(\text{'input unit inactive'}) = 1 - f_{\text{in}}$, respectively, should, nevertheless, sum up to the functional connectivity c:

$$\begin{aligned} &\mathcal{P}(\textit{'connection functional'}) \\ &= \rho_g(u) \cdot \mathcal{P}(\textit{'input unit active'}) + \rho_n(u) \cdot \mathcal{P}(\textit{'input unit inactive'}) = c \quad \forall u. \end{aligned} \tag{S1.7}$$

Output unit never active after learning the k-th pattern ($u = 0$): For $\rho_g(0)$ and $\rho_n(0)$, only the storage of the k-th pattern plays a role. Starting from a random fraction c (thus a total number of cN_{in}) of functional connections targeting each output unit (blue parts in Fig S1.2A), we first consider connections only from an active input unit (left part, titled f_{in}, in Fig S1.2). We can split up the probability of a genuine-genuine connection to become functional into the probabilities of two mutually exclusive conditions and their corresponding conditional probabilities (see Eq (S1.1)). We have the two conditions

(i) $\mathcal{P}(\textit{'g-g connection previously functional'}) = c$

(ii) $\mathcal{P}($*'g-g connection previously silent'*$) = c_m - c$,

for each of which we compute the conditional probability of having a functional genuine-genuine connection after learning:

(i) If the connection is already functional and the corresponding input unit is active, the connection is protected and remains functional:
$\mathcal{P}($*'g-g connection functional'* $|$ *'g-g connection previously functional'*$) = 1$
(yielding the blue part in Fig S1.2B left)

(ii) If the connection is silent and the corresponding input unit is active, the connection is made functional with transition probability η:
$\mathcal{P}($*'g-g connection functional'* $|$ *'g-g connection previously silent'*$) = \eta$
(yielding the green part in Fig S1.2B)

According to the law of total probability, the probability of a functional genuine-genuine connection is thus

$$\rho_g(0) = 1 \cdot c + \eta \cdot (c_m - c), \tag{S1.8}$$

which is depicted by the left orange part in Fig S1.2B.

Analogously, the probability of a functional spurious-genuine connection after learning is derived (right part, titled $1 - f_{\text{in}}$, in Fig S1.2). Given that the corresponding input unit is inactive, the two conditions have the probabilities

(iii) $\mathcal{P}($*'s-g connection previously silent'*$) = c_m - c$

(iv) $\mathcal{P}($*'s-g connection previously functional'*$) = c$,

and the corresponding conditional probabilities are the following:

(iii) If the connection is silent and the input unit is inactive, the connection remains silent:
$\mathcal{P}($*'s-g connection functional'* $|$ *'s-g connection previously silent'*$) = 0$
(represented by the gray part on the right of Fig S1.2B)

(iv) If the connection is functional and the input unit is inactive, the connection could be silenced due to the homeostasis mechanism (represented by the red frame in Fig S1.2B) or could remain functional. As before (same case (iv)), the probability of connections to remain functional is
$\mathcal{P}($*'s-g connection functional'* $|$ *'s-g connection previously functional'*$)$
$= 1 - \frac{\eta f_{\text{in}}(c_m - c)}{c(1-f_{\text{in}})}$
(yielding the blue part without the red frame in Fig S1.2B right).

Then, the total probability of a functional spurious-genuine connection is

$$\rho_n(0) = 0 \cdot (c_m - c) + \left(1 - \frac{\eta f_{\text{in}}(c_m - c)}{c(1 - f_{\text{in}})}\right) \cdot c = c - \frac{c_m - c}{1 - f_{\text{in}}} \eta f_{\text{in}}, \tag{S1.9}$$

which is represented by the right orange part in Fig S1.2B. By using Eqs (S1.8) and (S1.9) in Eq (S1.7), we see that the total probability of a functional connection to a genuine unit after learning the k-th pattern is again

$$\mathcal{P}(\text{'connection functional'}) = \rho_g(0) \cdot f_{\text{in}} + \rho_n(0) \cdot (1 - f_{\text{in}}) = c. \quad \checkmark \tag{S1.10}$$

Output unit active once after learning the k-th pattern ($u = 1$): Next, we derive $\rho_g(1)$ and $\rho_n(1)$, which correspond to the probabilities that a genuine-genuine or a spurious-genuine connection (with respect to the k-th pattern) is functional under the condition that the respective output unit is active exactly once among all subsequent patterns. The derivation of $\rho_g(1)$ and $\rho_n(1)$ is more involved because functional connections that originated from an active input unit in the k-th pattern (genuine-genuine connections) can be silenced while storing additional patterns, and in turn silent connections originating from inactive input units in the k-th pattern (spurious-genuine connections) can be made functional. We assume that the genuine output unit that we are investigating is active only for one pattern $k + \kappa$ with $\kappa \in \mathbb{N}^+$ fixed.

Let us start with the derivation of $\rho_g(1)$. The fraction of genuine-genuine connections being functional after storing pattern $k + \kappa$ (in addition to pattern k) can be split into four conditions depending on the previous state of the connection (i.e., depending on $\rho_g(0)$) and on the activity state of the corresponding input unit in pattern $k + \kappa$. The four conditions have the probabilities

(i) $\mathcal{P}(\text{‘}\textit{g-g connection previously functional and input unit active}\text{’}) = \rho_g(0) f_{\text{in}}$

(ii) $\mathcal{P}(\text{‘}\textit{g-g connection previously silent and input unit active}\text{’}) = (c_m - \rho_g(0)) f_{\text{in}}$

(iii) $\mathcal{P}(\text{‘}\textit{g-g connection previously silent and input unit inactive}\text{’})$
$= (c_m - \rho_g(0))(1 - f_{\text{in}})$

(iv) $\mathcal{P}(\text{‘}\textit{g-g connection previously functional and input unit inactive}\text{’}) = \rho_g(0)(1 - f_{\text{in}})$

and the corresponding conditional probabilities are

(i) If the connection is already functional before storing pattern $k + \kappa$ and the corresponding input unit is active, the connection is protected and remains functional:
$\mathcal{P}(\text{‘}\textit{g-g connection functional}\text{’} \mid \text{‘}\textit{g-g connection previously functional and input unit active}\text{’}) = 1$
(represented by the green frame in Fig S1.2C left)

(ii) If the connection is silent and the corresponding input unit is active in pattern $k + \kappa$, the connection is made functional with transition probability η:
$\mathcal{P}(\text{‘}\textit{g-g connection functional}\text{’} \mid \text{‘}\textit{g-g connection previously silent and input unit active}\text{’}) = \eta$
(yielding the green area in Fig S1.2C left)

(iii) If the connection is silent and the input unit is inactive, the connection remains silent:
$\mathcal{P}(\text{‘}\textit{g-g connection functional}\text{’} \mid \text{‘}\textit{g-g connection previously silent and input unit inactive}\text{’}) = 0$ (represented by the gray part on the left of Fig S1.2C)

(iv) If the connection is functional and the input unit is inactive, the connection remains functional with probability
$\mathcal{P}(\text{‘}\textit{g-g syn. functional}\text{’} \mid \text{‘}\textit{g-g syn. previously functional and input unit inactive}\text{’})$
$= 1 - \frac{\eta f_{\text{in}}(c_m - c)}{c(1 - f_{\text{in}})}$
(shown by the orange area without the red frame and without the green frame in Fig S1.2C left).

Using the law of total probability, we thus have

$$\rho_g(1) :=1 \cdot \rho_g(0) f_{\text{in}} + \eta \cdot (c_m - \rho_g(0))\, f_{\text{in}} + 0 \cdot (c_m - \rho_g(0))\,(1 - f_{\text{in}}) \tag{S1.11}$$

$$+ \left(1 - \frac{\eta f_{\text{in}}(c_m - c)}{c(1 - f_{\text{in}})}\right) \rho_g(0)(1 - f_{\text{in}}) \tag{S1.12}$$

$$= \rho_g(0) \left(1 - \frac{\eta f_{\text{in}} c_m}{c}\right) + \eta f_{\text{in}} c_m, \tag{S1.13}$$

shown by the purple parts in Fig S1.2C left.

Analogously, the probability $\rho_n(1)$ of spurious-genuine connections being functional after storing pattern $k + \kappa$ (in addition to pattern k) consists of four conditions with probabilities

(v) $\mathcal{P}($*'s-g connection previously functional and input unit active'* $) = \rho_n(0) f_{\text{in}}$

(vi) $\mathcal{P}($*'s-g connection previously silent and input unit active'* $) = (c_m - \rho_n(0)) f_{\text{in}}$

(vii) $\mathcal{P}($*'s-g connection previously silent and input unit inactive'* $)$
$= (c_m - \rho_n(0))(1 - f_{\text{in}})$

(viii) $\mathcal{P}($*'s-g connection previously functional and input unit inactive'* $) = \rho_n(0)(1 - f_{\text{in}})$

and the four corresponding conditional probabilities

(v) $\mathcal{P}($*'s-g connection functional'* $\mid$ *'s-g connection previously functional and input unit active'* $) = 1$
(represented by the green frame in Fig S1.2C right)

(vi) $\mathcal{P}($*'s-g connection functional'* $\mid$ *'s-g connection previously silent and input unit active'* $) = \eta$
(yielding the green area in Fig S1.2C right)

(vii) $\mathcal{P}($*'s-g connection functional'* $\mid$ *'s-g connection previously silent and input unit inactive'* $) = 0$ (represented by the gray part on the right of Fig S1.2C)

(viii) $\mathcal{P}($*'s-g syn. functional'* $\mid$ *'s-g syn. previously functional and input unit inactive'* $)$
$= 1 - \frac{\eta f_{\text{in}}(c_m - c)}{c(1 - f_{\text{in}})}$
(represented by the orange area without the red frame and without the green frame in Fig S1.2C right)

According to the law of total probability, the probability of a functional spurious-genuine connection amounts to

$$\rho_n(1) :=1 \cdot \rho_n(0) f_{\text{in}} + \eta \cdot (c_m - \rho_n(0))\, f_{\text{in}} + 0 \cdot (c_m - \rho_n(0))\,(1 - f_{\text{in}}) \tag{S1.14}$$

$$+ \left(1 - \frac{\eta f_{\text{in}}(c_m - c)}{c(1 - f_{\text{in}})}\right) \rho_n(0)(1 - f_{\text{in}}) \tag{S1.15}$$

$$= \rho_n(0) \left(1 - \frac{\eta f_{\text{in}} c_m}{c}\right) + \eta f_{\text{in}} c_m, \tag{S1.16}$$

which corresponds to the purple parts in Fig S1.2C right.

Generalization for any output unit usage $u > 0$. The probability of a functional connection, if the corresponding output unit is active u times across the whole pattern set, can be computed recursively. The conditional probabilities remain the same in every step, while the four conditions always depend on the probabilities $\rho_g(u-1)$ and $\rho_n(u-1)$ of the previous step. We thus obtain $\rho_g(2)$ from $\rho_g(1)$ in the same way as we obtained $\rho_g(1)$ from $\rho_g(0)$,

$$\rho_g(2) = \rho_g(1)\left(1 - \frac{\eta f_{\mathrm{in}} c_m}{c}\right) + \eta f_{\mathrm{in}} c_m \ , \tag{S1.17}$$

and in general we get

$$\rho_g(u) = \rho_g(u-1)\left(1 - \frac{\eta f_{\mathrm{in}} c_m}{c}\right) + \eta f_{\mathrm{in}} c_m. \tag{S1.18}$$

$\rho_n(u)$ can be derived in the same way and we have

$$\rho_n(u) = \rho_n(u-1)\left(1 - \frac{\eta f_{\mathrm{in}} c_m}{c}\right) + \eta f_{\mathrm{in}} c_m. \tag{S1.19}$$

Thus, the recursive equations for $\rho_n(u)$ and $\rho_g(u)$ are identical, but the initial values $\rho_n(0) \neq \rho_g(0)$ are different (see Eqs (S1.8) and (S1.9)).

From the recursive description of $\rho_g(u)$ (Eqs (S1.8) and (S1.18)), we can derive the explicit description

$$\rho_g(u) = (c_m - c)\eta\left(1 - \frac{f_{\mathrm{in}}\eta c_m}{c}\right)^u + c. \tag{S1.20}$$

Examples of the probability $\rho_g(u)$ that a genuine-genuine connection is functional are shown in Fig S1.3 as a function of the output unit usage u.

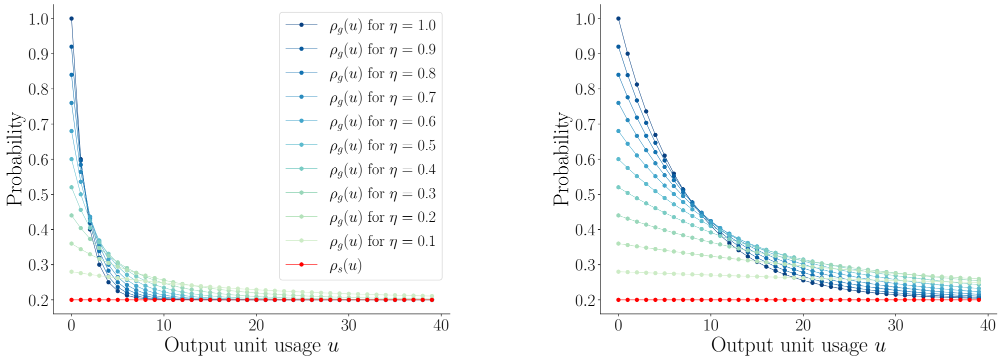

Fig S1.3. Probabilities of functional connections $\rho_g(u)$ and $\rho_s(u)$. Probability $\rho_l(u)$, $l \in \{s, g\}$, of a connection to be functional depending on output unit usage u for various transition probabilities η. Blue dots: genuine-genuine connections (Eq (S1.20)); red dots: genuine-spurious connections (Eq (S1.5)). Left: $f_{\mathrm{in}} = 0.1$, right: $f_{\mathrm{in}} = 0.025$. Probability ρ_s of genuine-spurious connections to be functional is constant as a function of u. Probability ρ_g of genuine-genuine connections to be functional decreases exponentially with u. Other parameters: $c = 0.2$, $c_m = 1$.

Analogously, the explicit description of ρ_n is

$$\rho_n(u) = \frac{-f_{\mathrm{in}}\eta(c_m - c)}{1 - f_{\mathrm{in}}}\left(1 - \frac{f_{\mathrm{in}}\eta c_m}{c}\right)^u + c, \tag{S1.21}$$

(see Fig S1.4 for examples).

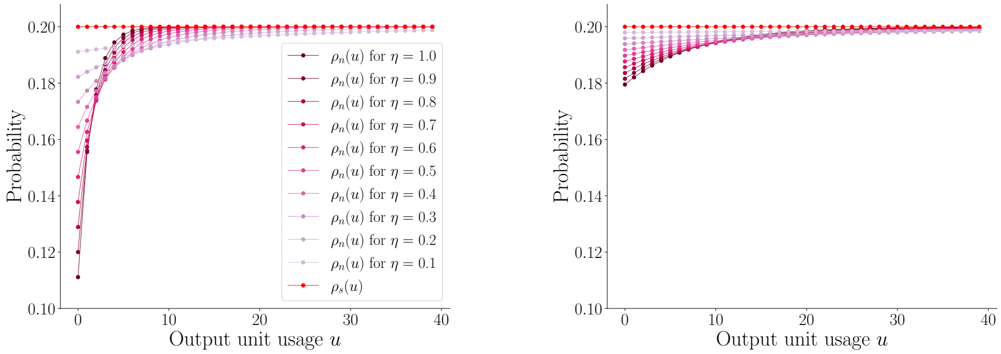

Fig S1.4. Probabilities of functional connections $\rho_n(u)$ and $\rho_s(u)$. Probability $\rho_l(u)$, $l \in \{n, s\}$, of a connection to be functional depending on output unit usage u. Pink shades: spurious-genuine connections ($\rho_n(u)$, Eq (S1.21)); red: connections to spurious units ($\rho_s(u)$, Eq (S1.5)). Left: $f_{\text{in}} = 0.1$, right: $f_{\text{in}} = 0.025$. Probability ρ_s of genuine-spurious connections to be functional is constant as a function of u. Probability ρ_n of spurious-genuine connections to be functional increases exponentially with u. Other parameters: $c = 0.2$, $c_m = 1$.

References

1. Buckingham JT. Delicate Nets, Faint Recollections: A Study of Partially Connected Associative Network Memories [PhD thesis]. University of Edinburgh; 1991.

2. Buckingham J, Willshaw D. Performance characteristics of the associative net. Network: Computation in Neural Systems. 1992;3(4):407–414.

3. Palm G, Sommer FT. Associative data storage and retrieval in neural networks. In: Models of Neural Networks III: Association, Generalization, and Representation. Springer; 1996. p. 79–118.

4. Bosch H, Kurfess FJ. Information storage capacity of incompletely connected associative memories. Neural Networks. 1998;11(5):869–876.

5. Knoblauch A. Neural associative memory and the Willshaw–Palm probability distribution. SIAM Journal on Applied Mathematics. 2008;69(1):169–196.
