# Supplementary material for: Population sparseness determines strength of Hebbian plasticity for maximal memory lifetime in associative networks
Source: PLoS Comput Biol. 2026 Jul 6;22(7):e1013235. doi: 10.1371/journal.pcbi.1013235 (PMC13390959; doi:10.1371/journal.pcbi.1013235)
Supplement: S2 Appendix — Discussion of the multimodality of the distributions of dendritic sums. (PDF) [file pcbi.1013235.s008.pdf]

S2 Appendix

Analysis of shape of distributions

The distributions of the dendritic sums are not necessarily simple binomial distributions. For many parameter combinations, the distributions are multi-modal and hence the means and standard deviations alone are not useful to characterize the distributions. In the following, we first discuss the multimodality of the distributions using an example. Further, we investigate in which cases the distributions are similar to binomial distributions and in which cases they are not, and we analyze the shape of the distributions in the latter case in more detail. We suggested an approximation using a single binomial distribution, which was necessary to calculate the capacity of the network. This Appendix supports the derivations in the Methods by arguing why the approximation by a single binomial distribution could be satisfactory.

Location of bumps — an example

To better understand the location and magnitude of bumps of the distribution of net inputs to a genuine unit, $p_g^{[P]}$, let us first consider one specific example (see Fig S2.1) with parameters:

$$N_{\text{in}} = 1000, f_{\text{in}} = f_{\text{out}} = 0.1, \eta = 0.8, c = 0.2, c_m = 1, P = 10. \tag{S2.1}$$

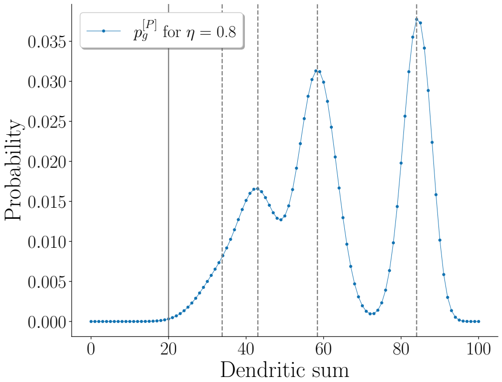

Fig S2.1. Bumps in distribution of dendritic sums for a genuine output unit. Vertical dashed gray lines correspond to the locations of the peaks of the first four bumps. The center of the right-most peak is at $\rho_g(0)M_{\text{in}} = 84$, the second at $\rho_g(1)M_{\text{in}} = 58.4$, etc. For large values of u, the value of $\rho_g(u)$ asymptotically approaches c, and then there would be only a single bump at $cM_{\text{in}} = 20$ (solid vertical line). Parameters: $f_{\text{in}} = 0.1, f_{\text{out}} = 0.1, N_{\text{in}} = 1000, c = 0.2, c_m = 1, \eta = 0.8, P = 10$.

For a small P, such as $P = 10$ here, a large group of genuine output units are active only during the initial storage of a pattern (here also called the k-th pattern) and not in any of the other ten patterns. In fact, the probability that an output unit is active in no subsequent pattern is $\mathcal{B}_{10,0.1}(0) \approx 0.35$, which is the weight of the rightmost binomial distribution $\mathcal{B}_{M_{\text{in}},\rho_g(0)}$. To understand the location of its peak, let us recall that when the k-th pattern was learned, the silent genuine-genuine connections became functional with the transition probability $\eta = 0.8$. In addition, cM_{in} genuine-genuine connections per output unit were already functional due to the initial random connectivity of cN_{in} connections per output unit. No connections to genuine units were silenced. In total, the genuine units hence receive on average a net input of slightly more than

$M_{\text{in}} \cdot \eta = 100 \cdot 0.8 = 80$, namely

$$\rho_g(0) \cdot M_{\text{in}} = (c + (c_m - c)\eta)M_{\text{in}} = (0.2 + (1 - 0.2) \cdot 0.8) \cdot 100 = 84. \tag{S2.2}$$

This is the location of the right-most peak of the distribution.

Some genuine output units are active once across all additional P patterns, say in pattern $k + \kappa$. This decreases their dendritic sum because some of the functional genuine-genuine connections (of the k-th pattern) are silenced in order to counteract the connections that became functional for pattern $k + \kappa$ and maintain the functional connectivity level c. Note that, nevertheless, a few previously silent genuine-genuine connections of the k-th pattern might be made functional if there is an overlap between the active input units of the k-th pattern and the active input units of the other pattern $k + \kappa$ for which the respective output unit is active. Overall, the average number of connections that remain functional and receive input, thus contributing to the dendritic sum, is

$$\rho_g(1) \cdot M_{\text{in}} = \left(0.2 + (1 - 0.2) \cdot 0.8 \cdot \left(1 - \frac{0.1 \cdot 0.8 \cdot 1}{0.2}\right)\right) \cdot 100 = 58.4, \tag{S2.3}$$

which is the location of the second peak from the right of the distribution of the dendritic sums in Fig S2.1. In our example, an output unit is active $f_{\text{out}} \cdot P = 0.1 \cdot 10 = 1$ time on average. The mass that belongs to the second bump from the right is $\mathcal{B}_{10,\,0.1}(1) \approx 0.39$, which is the largest weight because $\mathcal{B}_{10,\,0.1}(1) > B_{10,\,0.1}(u)$ holds, for any $u \neq 1$.

The third peak from the right corresponds to $\rho_g(2)M_{\text{in}} = 43.04$ with a weight of $\mathcal{B}_{10,\,0.1}(2) \approx 0.19$. The remaining peaks are not discriminable and are merging into this third bump.

The width of the peaks can be described by the standard deviation of $\mathcal{B}_{M_{\text{in}},\rho_g(u)}$:

$$\sqrt{M_{\text{in}}\, \rho_g(u)\, (1 - \rho_g(u))}, \tag{S2.4}$$

where u is the output unit usage. Typically, the width first increases with increasing u and then decreases again, but at some point the distance between the peaks becomes smaller than their width and the oscillatory structure fades out. This will be discussed in more detail in the next section.

Multimodality of distributions

For spurious units, the probability of a functional connection $\rho_s(u) = c$ is constant as a function of the output unit usage u. The distribution of dendritic sums p_s is thus a binomial distribution and always has a single peak (see Eq (44) in main text). For genuine units, $\rho_g(u)$ is not constant. It decays exponentially with the output unit usage u, also approaching the functional connectivity c (see Fig 9). The distribution of dendritic sums $p_g^{[P]}$ is thus a sum of several different binomial probability mass functions (PMFs) $\mathcal{B}_{M_{\text{in}},\rho_g(u)}$, which are weighted by prefactors $\mathcal{B}_{P,f_{\text{out}}}(u)$ (see Eq (46) in main text).

The multimodality of the distribution of the dendritic sums of genuine units depends on two aspects, which are discussed in this section:

- First, multimodality depends on the weights $\mathcal{B}_{P,f_{\text{out}}}(u)$ in Eq (46) (black curves in Fig S2.2). Important is how many u values have a large weight because each large weight adds a binomial distribution $\mathcal{B}_{M_{\text{in}},\rho_g(u)}$ in Eq (46) to the total distribution of dendritic sums. The more binomials $\mathcal{B}_{M_{\text{in}},\rho_g(u)}$ in Eq (46) have a large weight $\mathcal{B}_{P,f_{\text{out}}}(u)$, the more distinct bumps could occur in the total distribution.

- Second, multimodality depends on how different the distributions $\mathcal{B}_{M_{\mathrm{in}},\rho_g(u)}$ are, which is determined by the $\rho_g(u)$-values (blue curves in Fig S2.2). It is therefore important how different the $\rho_g(u)$-values are from each other, i.e., how steep the slope of the blue curves in Fig S2.2 is, in particular in the range of u values for which $\mathcal{B}_{P,f_{\mathrm{out}}}(u)$ is large (black curves in Fig S2.2). For example, the more different $\rho_g(u)$ is from $\rho_g(u+1)$, the more the contributions from $\mathcal{B}_{M_{\mathrm{in}},\rho_g(u)}$ and $\mathcal{B}_{M_{\mathrm{in}},\rho_g(u+1)}$ appear as distinct bumps in the total distribution. How well separated the bumps appear is also determined by the widths of the two binomials.

As discussed in the previous subsection for the example in Fig S2.1, the values of ρ_g determine the locations of the peaks. The first bump (centered at $M_{\mathrm{in}}\rho_g(0)$) represents the probability of the connection being functional if the output unit has never been active across all P additional patterns, the second bump (centered at $M_{\mathrm{in}}\rho_g(1)$) represents the probability of the connection being functional if the output unit has been active once across the pattern set, etc.

Different values $\rho_g(u)$, for $u \in \{1, \ldots, P\}$, lead to different contributions to the distribution of dendritic sums. The contributions depend on the corresponding values of $\mathcal{P}(r = u) = \mathcal{B}_{P,\, f_{\mathrm{out}}}(u)$ (cf. Eq (46)). In general, since only values of u that are less than or equal to P give a probability $\mathcal{B}_{P,\, f_{\mathrm{out}}}(u) > 0$, the number of $\rho_g(u)$-values that contribute to the overall distribution at all is small if P is small. Fig S2.2 shows the PMF $\mathcal{B}_{P,\, f_{\mathrm{out}}}(u)$ for $f_{\mathrm{out}} = 0.1$ (black, solid line) and the corresponding $\rho_g(u)$ for various transition probabilities η.

The PMF $\mathcal{B}_{P,\, f_{\mathrm{out}}}$ depends on P and f_{out} but — for f_{out} small enough — mostly on the product $P \cdot f_{\mathrm{out}}$. If f_{out} is decreased by a factor 10 (Fig S2.2, dark gray dashed line) or 100 (Fig S2.2, light gray dotted line) and P is increased accordingly by a factor 10 or 100, the PMFs look very similar even though their standard deviations are not exactly the same.

If the contribution of several $\rho_g(u)$-values is large and if these values are not too similar to each other for different u, each of them will create a separate bump in the distribution of the dendritic sums. For smaller u, the values of $\rho_g(u)$ are more different from each other than for larger u. They asymptotically approach c and hence become more and more similar with increasing u (cf. Fig 9). The range of u-values for which $\mathcal{B}_{P,\, f_{\mathrm{out}}}(u)$ is large moves to the right with increasing P (Fig S2.2). For small P, several $\rho_g(u)$-values that are very different from each other determine the distribution; this gives rise to clearly distinct bumps. Increasing P leads to the strongly contributing $\rho_g(u)$-values being increasingly similar and making the distribution loose its multi-modality.

The larger η, the larger the first value $\rho_g(0)$ and hence the more different the first few values are from each other and from the asymptotic value c. The exponential decay of $\rho_g(u)$ has a steeper slope the larger η is (cf. Fig 9 and Fig S2.2). If η is small we have $\rho_g(0) \gtrsim c$, and thus the decay of $\rho_g(u)$ with increasing u is gentle; and in the distribution of dendritic sum no visually distinct bumps occur. In this case, this distribution resembles a binomial distribution. In contrast, large η-values can lead to distributions that are less similar to a binomial distribution (at least for small P).

How different the $\rho_g(u)$-values must be such that they appear as separate bumps in the distribution and are not perceived as one binomial-like bump depends on the distance between two subsequent peaks compared to the width of the bumps (cf. Fig S2.3). If the width of the bumps is much larger than the distance between the peaks, they merge into one joint bump. The width of a single bump at $M_{\mathrm{in}}\rho_g(u)$ can be determined by the standard deviation of the corresponding binomial distribution that describes the probability of the connection being functional given a specific output unit

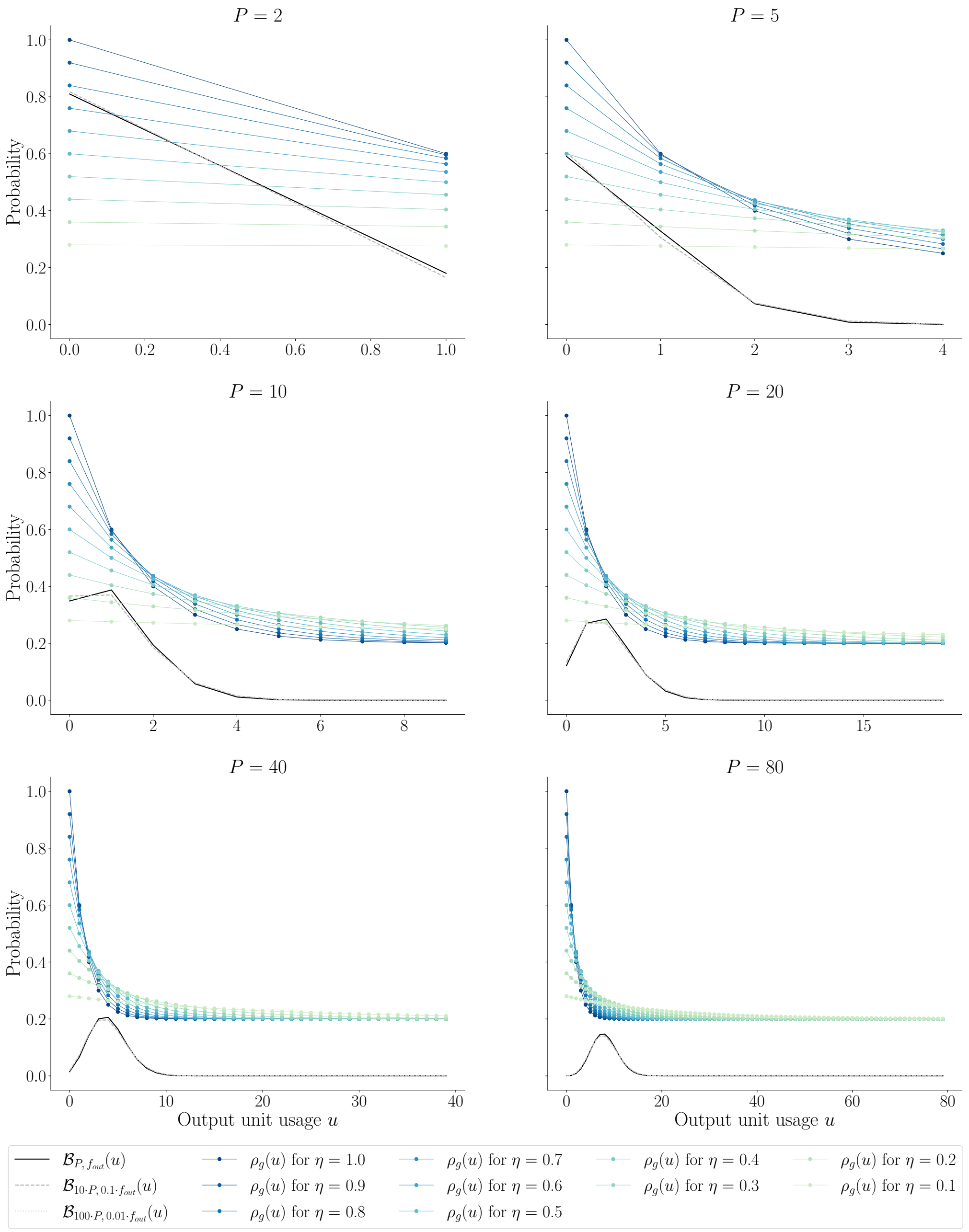

Fig S2.2. Probability of functional connection $\rho_g(u)$ and corresponding weight $\mathcal{B}_{P,\,f_{\text{out}}}(u)$.
The probability $\mathcal{B}_{P,\,f_{\text{out}}}(u)$ that an output unit is on u times across $P(= 2, 5, 10, 20, 40, 80)$ patterns and the corresponding $\rho_g(u)$-values for various η-values. Black, solid: $f_{\text{out}} = 0.1$; dark gray, dashed: $f_{\text{out}} = 0.01$ and given P-value increased by factor 10; light gray, dotted: $f_{\text{out}} = 0.001$ and given P-value increased by factor 100. The values of u for which the weight $\mathcal{B}_{P,f_{\text{out}}}(u)$ is large increase with increasing P. (Other parameters: $N_{\text{in}} = 1000, f_{\text{in}} = 0.1, f_{\text{out}} = 0.1, c = 0.2, c_m = 1$.)

usage u (cf. Eq (41)):

$$\sqrt{M_{\text{in}}\rho_g(u)(1-\rho_g(u))} \tag{S2.5}$$

$$= \sqrt{N_{\text{in}} f_{\text{in}} \left[(c_m - c)\eta \left(1 - \frac{f_{\text{in}}\eta c_m}{c}\right)^u + c\right] \left[1 - (c_m - c)\eta \left(1 - \frac{f_{\text{in}}\eta c_m}{c}\right)^u - c\right]}.$$

The distance between the peaks u and $u+1$ is given by

$$M_{\text{in}} \left|\rho_g(u+1) - \rho_g(u)\right| = M_{\text{in}} \left(\rho_g(u) - \rho_g(u+1)\right) \tag{S2.6}$$

$$= M_{\text{in}} \left(\rho_g(u) \left(1 - 1 + \frac{f_{\text{in}}\eta c_m}{c}\right) - f_{\text{in}}\eta c_m\right) = N_{\text{in}} f_{in}^2 \eta c_m \left(\rho_g(u)\frac{1}{c} - 1\right) \tag{S2.7}$$

$$= N_{\text{in}} f_{\text{in}}^2 \eta \frac{c_m}{c} \left((c_m - c)\eta \left(1 - \frac{f_{\text{in}}\eta c_m}{c}\right)^u + c - c\right) \tag{S2.8}$$

$$= N_{\text{in}} f_{\text{in}}^2 \eta^2 \frac{c_m(c_m - c)}{c} \left(1 - \frac{f_{\text{in}}\eta c_m}{c}\right)^u. \tag{S2.9}$$

We define distributions for which the ratio of the distance between the peaks and the standard deviation

$$\frac{M_{\text{in}} \left|\rho_g(u+1) - \rho_g(u)\right|}{\sqrt{M_{\text{in}}\rho_g(u)(1-\rho_g(u))}} \tag{S2.10}$$

is smaller than 1 for all $u \geq 0$ as *sufficiently binomial-like.* For $u = 0$ and the special case $\eta = 1$, the ratio of the distance between the peaks and the standard deviation is infinite because the standard deviation is zero since the distribution reduces to $\mathcal{P}(d_g = M_{\text{in}}) = 1$. It can be observed that this ratio decreases with increasing u. For $0 < \eta < 1$, we can thus investigate for which parameters the ratio is smaller than 1 at $u = 0$ and imply that it will also be smaller than 1 for any $u > 0$. We have

$$\frac{M_{\text{in}} \left|\rho_g(1) - \rho_g(0)\right|}{\sqrt{M_{\text{in}}\rho_g(0)(1-\rho_g(0))}} = \frac{N_{\text{in}} f_{\text{in}}^2 \eta^2 \frac{c_m(c_m-c)}{c}}{\sqrt{N_{\text{in}} f_{\text{in}} \left((c_m - c)\eta + c\right)\left(1 - (c_m - c)\eta - c\right)}} \tag{S2.11}$$

$$= \frac{\sqrt{N_{\text{in}} f_{\text{in}}} f_{\text{in}} \eta^2 c_m (c_m - c)}{c\sqrt{\left((c_m - c)\eta + c\right)\left(1 - (c_m - c)\eta - c\right)}} \tag{S2.12}$$

and hence

$$\frac{M_{\text{in}} \left|\rho_g(1) - \rho_g(0)\right|}{\sqrt{M_{\text{in}}\rho_g(0)(1-\rho_g(0))}} \ll 1 \tag{S2.13}$$

$$\Leftrightarrow N_{\text{in}} f_{\text{in}}^3 \ll \frac{c^2 \left((c_m - c)\eta + c\right)\left(1 - (c_m - c)\eta - c\right)}{\eta^4 c_m^2 (c_m - c)^2}. \tag{S2.14}$$

Making further biologically plausible assumptions, namely $c_m = 2c$ and $c \ll 1$, we arrive at the simpler condition

$$N_{\text{in}} f_{\text{in}}^3 \eta^4 c \ll \frac{1}{4}. \tag{S2.15}$$

To summarize, the width of the distribution $\mathcal{B}_{P, f_{\text{out}}}$, which determines the number of $\rho_g(u)$-values with a significant contribution, in combination with the normalized distance between the single peaks determine the multimodality of the distribution $p_l^{[P]}$.

In order to analytically derive the capacity of the network in the Methods, it is necessary to approximate the weighted sum of binomial PMFs by a single binomial PMF. This approximation is only justified if the original distribution is *sufficiently binomial-like.* Based on Eq (S2.15), we find that such an approximation is best for small N_{in} and, in particular, for small f_{in} and small η as well as small c.

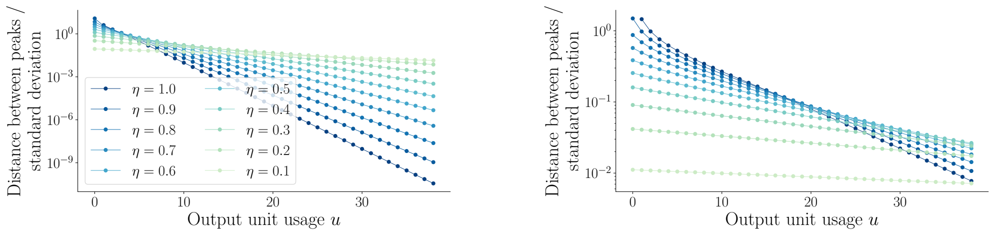

Fig S2.3. Distance between peaks of the bumps divided by the standard deviation of the bumps.

Distance between peaks of the bumps (Eq (S2.6)) divided by the standard deviation of the bumps (Eq (S2.5)) for various η values for genuine units decreases with output unit usage u. Left: $f_{\text{in}} = 0.1$, right: $f_{\text{in}} = 0.025$. The ratio decreases with increasing output unit usage u and with decreasing transition probability η. (It is ∞ for $u = 0$ and $\eta = 1$.) Further parameter values: $N_{\text{in}} = 1000, c = 0.2, c_m = 1$.
