# Supplementary material for: Population sparseness determines strength of Hebbian plasticity for maximal memory lifetime in associative networks
Source: PLoS Comput Biol. 2026 Jul 6;22(7):e1013235. doi: 10.1371/journal.pcbi.1013235 (PMC13390959; doi:10.1371/journal.pcbi.1013235)
Supplement: S3 Appendix — Numerical and analytical calculation of the signal quality as a function of P, including an analytical approximation of the signal quality with noisy input patterns. (PDF) [file pcbi.1013235.s009.pdf]

S3 Appendix

Solution of the Signal Quality Equation

In order to analytically describe the signal quality $S_P = (1 - \overline{s_P})H_{\text{avg}}$ for a number of subsequent patterns P, we have to solve the Signal Quality Equation

$$F_s^{-1}\left(1 - \overline{s_P} f_{\text{out}}\right) = F_g^{[P]^{-1}}\left(\overline{s_P}(1 - f_{\text{out}})\right) \tag{S3.1}$$

(see also Eq (84) in the Methods of the manuscript) for $\overline{s_P}$. In this Appendix, we discuss how this could be approached numerically as well as analytically.

Numerical calculation of the signal quality

The Signal Quality Equation (S3.1) could be solved numerically. As discussed in the Methods, the CDFs F_s and $F_g^{[P]}$ are not invertible. Thus, we approximate the underlying discrete distributions

$$p_s = \mathcal{B}_{M_{\text{in}},c}, \tag{S3.2}$$

$$p_g = \sum_{u=0}^{P}\left[\mathcal{B}_{P,\,f_{\text{out}}}(u) \cdot \mathcal{B}_{M_{\text{in}},\,\rho_g(u)}(x)\right] \tag{S3.3}$$

by continuous distributions

$$p_s(x) \approx \mathcal{N}\left(M_{\text{in}}c, M_{\text{in}}c(1-c)\right)(x), \tag{S3.4}$$

$$p_g^{[P]}(x) \approx \sum_{u=0}^{P}\left[\mathcal{B}_{P,\,f_{\text{out}}}(u) \cdot \mathcal{N}\left(M_{\text{in}}\rho_g(u), M_{\text{in}}\rho_g(u)(1-\rho_g(u))\right)(x)\right]. \tag{S3.5}$$

This is a good approximation for large enough M_{in} and sparse but not too sparse connectivity c. Specifically, we have to assume

$$M_{\text{in}}c \gg 1,\; M_{\text{in}}(1-c) \gg 1 \text{ and} \tag{S3.6}$$

$$M_{\text{in}}\rho_g(u) \gg 1,\; M_{\text{in}}(1-\rho_g(u)) \gg 1, \tag{S3.7}$$

for all $u \in \{0, \dots, P\}$. These four assumptions reduce to the two assumptions

$$M_{\text{in}}c \gg 1 \text{ and } M_{\text{in}}(1-\rho_g(u)) \gg 1 \tag{S3.8}$$

because $\rho_g(u) \geq c$. Then, the Signal Quality Equation (S3.1) can be written as

$$\frac{F_g^{[P]}\left(F_s^{-1}\left(1 - \overline{s_P} f_{\text{out}}\right)\right)}{1 - f_{\text{out}}} = \overline{s_P} \tag{S3.9}$$

and easily solved numerically without any additional approximation steps. This semi-analytical strategy yields a better approximation of the results from network simulations (see Fig S3.1) than the signal quality based on a fully analytical description (see following subsection).

Analytical calculation of the signal quality

If we approximate both p_s and $p_g^{[P]}$ by a single normal distribution as outlined in the Methods, the Signal Quality Equation can be replaced by

$$\frac{\bar{\mu}_g^{[P]} - \mu_s}{\sqrt{2}\left(\sigma_s \cdot R_s + \bar{\sigma}_g^{[P]} \cdot R_g\right)} = 1, \tag{S3.10}$$

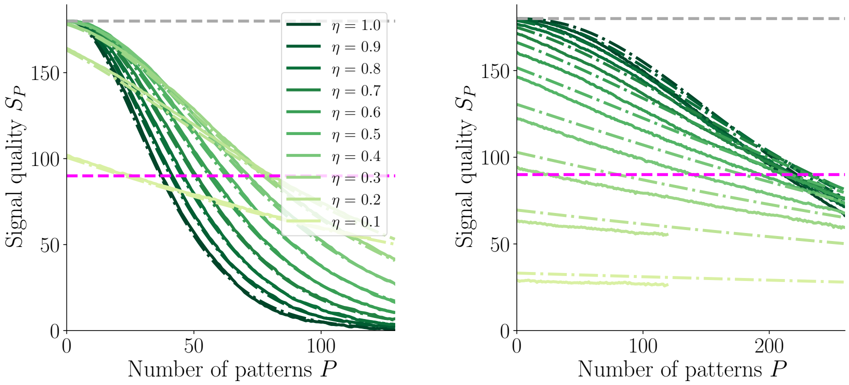

Fig S3.1. Comparison of signal quality obtained from network simulations and from a semi-analytical approximation.
Signal quality decays with the number P of subsequently learned patterns for various values of transition probability η. Solid lines: numerical network simulations dash-dotted lines: semi-analytical estimate (solution of Eq (S3.9) as discussed in this subsection) of the signal quality; dashed gray line: maximal signal quality, which is the average Hamming distance between two random f_{out}-sparse patterns; dashed magenta line: retrieval threshold. Left: $f_{\text{in}} = 0.1$, right: $f_{\text{in}} = 0.012$. Other parameters: $N_{\text{in}} = N_{\text{out}} = 1000$, $f_{\text{out}} = 0.1$, $c_m = 1$, $c = 0.2$.

where

$$R_s := \text{erf}^{-1}\left(1 - 2\overline{s_P} f_{\text{out}}\right), \tag{S3.11}$$

$$R_g := \text{erf}^{-1}\left(2\overline{s_P}(1 - f_{\text{out}}) - 1\right). \tag{S3.12}$$

Note that Eq (S3.10) is analytically solvable for $\overline{s_P}$ only if $f_{\text{out}} = 0.5$. In this case, we get[1]

$$s_P = \text{erf}\left(\frac{\bar{\mu}_g^{[P]} - \mu_s}{\sqrt{2}\left(\sigma_s + \bar{\sigma}_g^{[P]}\right)}\right) \tag{S3.13}$$

and, thus,

$$S_P = \text{erf}\left(\frac{\bar{\mu}_g^{[P]} - \mu_s}{\sqrt{2}\left(\sigma_s + \bar{\sigma}_g^{[P]}\right)}\right) H_{\text{avg}}, \tag{S3.14}$$

while for other $f_{\text{out}} \neq 0.5$, we need additional assumptions to solve Eq (S3.10).

---

[1]

$$\frac{\bar{\mu}_g^{[P]} - \mu_s}{\sqrt{2}\left(\sigma_s \cdot \text{erf}^{-1}\left(1 - \overline{s_P}\right) - \bar{\sigma}_g^{[P]} \cdot \text{erf}^{-1}\left(\overline{s_P} - 1\right)\right)} = 1$$

$$\Leftrightarrow \frac{\bar{\mu}_g^{[P]} - \mu_s}{\sqrt{2}\left(\sigma_s \cdot \text{erf}^{-1}\left(1 - \overline{s_P}\right) + \bar{\sigma}_g^{[P]} \cdot \text{erf}^{-1}\left(1 - \overline{s_P}\right)\right)} = 1$$

$$\Leftrightarrow \frac{\bar{\mu}_g^{[P]} - \mu_s}{\sqrt{2}\left(\sigma_s + \bar{\sigma}_g^{[P]}\right)} = \text{erf}^{-1}\left(1 - \overline{s_P}\right)$$

$$\Leftrightarrow 1 - \text{erf}\left(\frac{\bar{\mu}_g^{[P]} - \mu_s}{\sqrt{2}\left(\sigma_s + \bar{\sigma}_g^{[P]}\right)}\right) = \overline{s_P}$$

Approximation of $\bar{\sigma}_g^{[P]}$ by σ_s. For $f_{\text{out}} \neq 0.5$, Eq (S3.10) is not solvable. Comparing the variances of the genuine and the spurious distributions

$$\bar{\sigma}_g^{[P]\,2} = M_{\text{in}} \left[(c_m - c)\eta \left(1 - \frac{f_{\text{in}}\eta c_m}{c}\right)^{\lfloor f_{\text{out}}(P+1) \rfloor} + c \right] \tag{S3.15}$$

$$\cdot \left[1 - (c_m - c)\eta \left(1 - \frac{f_{\text{in}}\eta c_m}{c}\right)^{\lfloor f_{\text{out}}(P+1) \rfloor} - c \right], \tag{S3.16}$$

$$\sigma_s{}^2 = M_{\text{in}} c(1 - c), \tag{S3.17}$$

respectively, yields

$$\bar{\sigma}_g^{[P]\,2} - \sigma_s^2 = M_{\text{in}}(c_m - c)\eta \left(1 - \frac{f_{\text{in}}\eta c_m}{c}\right)^{\lfloor f_{\text{out}}(P+1) \rfloor} \tag{S3.18}$$

$$\cdot \left[1 - 2c - (c_m - c)\eta \left(1 - \frac{f_{\text{in}}\eta c_m}{c}\right)^{\lfloor f_{\text{out}}(P+1) \rfloor} \right] \tag{S3.19}$$

For $P \to \infty$, we have $\bar{\sigma}_g^{[P]} \to \sigma_s$ (Fig S3.2). The case of large P is particularly interesting for calculating the memory lifetime. We thus assume that, when S_P reaches the retrieval threshold, P is large enough for the assumption $\bar{\sigma}_g^{[P]} \approx \sigma_s$ to be reasonable. Eq (S3.10) now reads as

$$\frac{\bar{\mu}_g^{[P]} - \mu_s}{\sqrt{2}\sigma_s} = R_s + R_g. \tag{S3.20}$$

Note that for large η and small P, this assumption is less accurate (e.g., $\bar{\sigma}_g^{[0]} \to 0$ for $\eta \to 1$, while σ_s does not depend on η) (Fig S3.2).

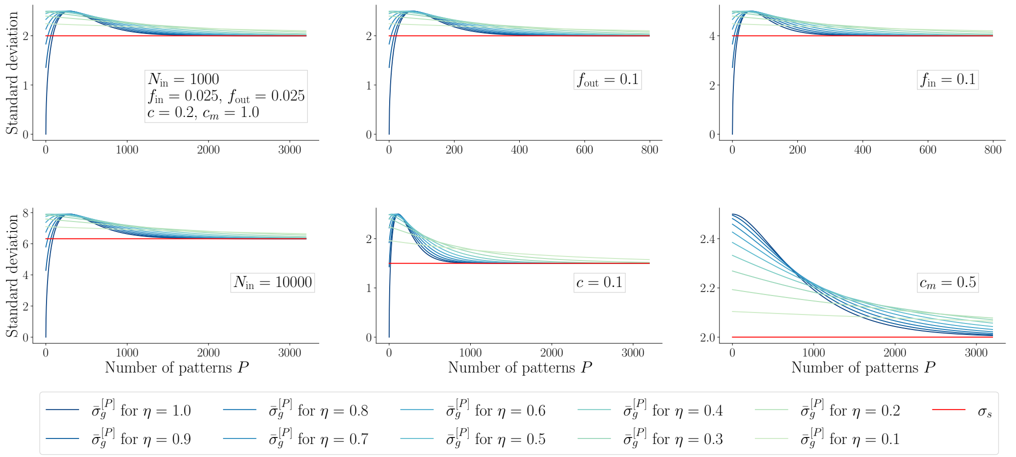

Fig S3.2. Comparison of $\bar{\sigma}_g^{[P]}$ and σ_s.
Comparison of the standard deviations of the genuine (blue lines) and the spurious (red lines) distributions of dendritic sums for various parameter combinations. Top left: default parameters $N_{\text{in}} = 1000, f_{\text{in}} = 0.025, f_{\text{out}} = 0.025, c = 0.2, c_m = 1$; top center: $f_{\text{out}} = 0.1$; top right: $f_{\text{in}} = 0.1$; bottom left: $N_{\text{in}} = 10000$; bottom center: $c = 0.1$; bottom right: $c_m = 0.5$.

Approximation of the error function. In order to solve Eq (S3.20) for $\overline{s_P}$, we now approximate the error function by

$$\mathrm{erf}(x) \approx \tanh\left(\frac{x\pi}{\sqrt{6}}\right) \tag{S3.21}$$

and use $\mathrm{arctanh}(x) = \frac{1}{2}\ln\left(\frac{1+x}{1-x}\right)$. This yields[2]

$$\frac{\bar{\mu}_g^{[P]} - \mu_s}{\sqrt{2}\sigma_s} = \frac{\sqrt{6}}{\pi}\left[\mathrm{arctanh}\left(1 - 2\overline{s_P} f_{\mathrm{out}}\right) - \mathrm{arctanh}\left(2\overline{s_P}(1 - f_{\mathrm{out}}) - 1\right)\right] \tag{S3.22}$$

$$\Leftrightarrow \overline{s_P} = \frac{-1 + \sqrt{1 + 4 f_{\mathrm{out}}(1 - f_{\mathrm{out}})\left(\exp\left(\frac{\pi}{\sqrt{3}}\frac{\bar{\mu}_g^{[P]} - \mu_s}{\sigma_s}\right) - 1\right)}}{2 f_{\mathrm{out}}(1 - f_{\mathrm{out}})\left(\exp\left(\frac{\pi}{\sqrt{3}}\frac{\bar{\mu}_g^{[P]} - \mu_s}{\sigma_s}\right) - 1\right)}. \tag{S3.23}$$

Note that the more accurate approximation of the error function employed in S4 Appendix (Eq (S4.42)) cannot be used here because it does not allow us to solve Eq (S3.20) for $\overline{s_P}$.

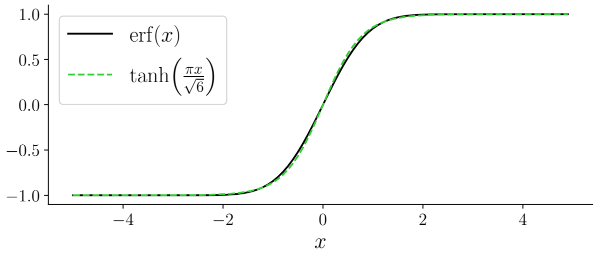

Fig S3.3. Approximation of error function by a hyperbolic tangent. The maximal absolute error is 0.0453, the maximal relative error is 0.1366.

The signal quality $S_P = s_P H_{\mathrm{avg}} = (1 - \overline{s_P}) H_{\mathrm{avg}}$ with $\overline{s_P}$ from Eq (S3.23) as a function of the number of patterns P is shown in Fig S3.4) (colored dashed lines). The

---

[2]

$$\begin{aligned}
\frac{\bar{\mu}_g^{[P]} - \mu_s}{\sqrt{2}\sigma_s} &= \frac{\sqrt{6}}{\pi}\left[\mathrm{arctanh}\left(1 - 2\overline{s_P} f_{\mathrm{out}}\right) - \mathrm{arctanh}\left(2\overline{s_P}(1 - f_{\mathrm{out}}) - 1\right)\right] \\
\Leftrightarrow \frac{\bar{\mu}_g^{[P]} - \mu_s}{\sqrt{2}\sigma_s} &= \frac{\sqrt{6}}{2\pi}\left[\ln\left(\frac{2 - 2\overline{s_P} f_{\mathrm{out}}}{2\overline{s_P} f_{\mathrm{out}}}\right) - \ln\left(\frac{2\overline{s_P}(1 - f_{\mathrm{out}})}{2 - 2\overline{s_P}(1 - f_{\mathrm{out}})}\right)\right] \\
\Leftrightarrow \frac{\bar{\mu}_g^{[P]} - \mu_s}{\sqrt{2}\sigma_s} &= \frac{\sqrt{6}}{2\pi}\left[\ln\left(\frac{(1 - \overline{s_P} f_{\mathrm{out}})(1 - \overline{s_P}(1 - f_{\mathrm{out}}))}{\overline{s_P} f_{\mathrm{out}} \overline{s_P}(1 - f_{\mathrm{out}})}\right)\right] \\
\Leftrightarrow \exp\left(\frac{\pi}{\sqrt{3}}\frac{\bar{\mu}_g^{[P]} - \mu_s}{\sigma_s}\right) &= \frac{1 - \overline{s_P} + \overline{s_P}^2 f_{\mathrm{out}}(1 - f_{\mathrm{out}})}{\overline{s_P}^2 f_{\mathrm{out}}(1 - f_{\mathrm{out}})} \\
\Leftrightarrow 0 &= \overline{s_P}^2 \cdot f_{\mathrm{out}}(1 - f_{\mathrm{out}})\left(\exp\left(\frac{\pi}{\sqrt{3}}\frac{\bar{\mu}_g^{[P]} - \mu_s}{\sigma_s}\right) - 1\right) + \overline{s_P} - 1 \\
\Leftrightarrow \overline{s_P} &= \frac{-1 + \sqrt{1 + 4 f_{\mathrm{out}}(1 - f_{\mathrm{out}})\left(\exp\left(\frac{\pi}{\sqrt{3}}\frac{\bar{\mu}_g^{[P]} - \mu_s}{\sigma_s}\right) - 1\right)}}{2 f_{\mathrm{out}}(1 - f_{\mathrm{out}})\left(\exp\left(\frac{\pi}{\sqrt{3}}\frac{\bar{\mu}_g^{[P]} - \mu_s}{\sigma_s}\right) - 1\right)}
\end{aligned}$$

As $\bar{\mu}_g^{[P]} > \mu_s$, the denominator is positive. The numerator also has to be positive to achieve $\overline{s_P} \in [0, 1]$. Since the term inside the square root is greater than one, the solution of the quadratic equation for $\overline{s_P}$ with the positive sign before the square root yields a valid $\overline{s_P}$. Using the negative sign before the square root in the numerator, which would of course also yield a solution of the second to last equation, would give a negative numerator and by that a negative $\overline{s_P}$ and should thus be neglected.

analytical estimate captures the trend of the lines and, most importantly, their relation to each other if different transition probabilities are compared. It matches the results of network simulations in the sense that the signal quality S_P for larger transition probability η is initially higher but decays more quickly with the number of patterns P. This yields different optimal transition probabilities depending on the input and output activation ratios f_{in} and f_{out}. The semi-analytical strategy presented in the Methods yields a better approximation of the results from network simulations than the signal quality obtained from Eq (S3.23) (compare Fig 17 to Fig S3.4). This benefit of the semi-analytical approximation compared to the fully analytical approximation is particularly prominent for large η, which is due to the fact that both the approximation of $p_g^{[P]}$ by a single binomial distribution and the approximation of $\bar{\sigma}_g^{[P]}$ by σ_s used in the derivation of Eq (S3.23) (see Methods) are less accurate for large η than for small η.

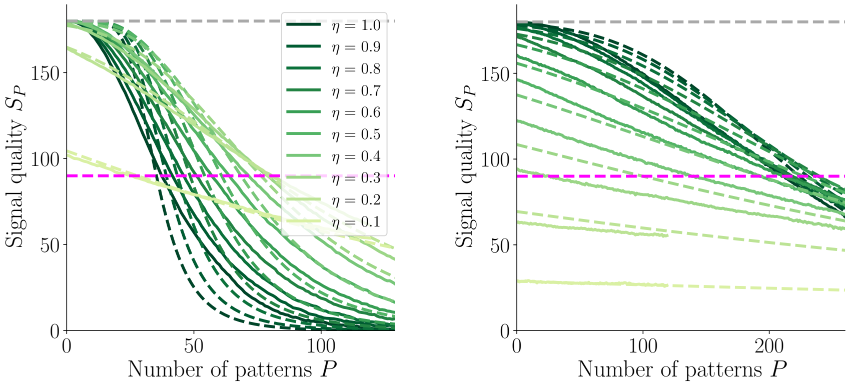

Fig S3.4. Comparison of signal quality obtained from network simulations and from analytical approximation.
Signal quality S_P decays with number P of subsequently learned patterns for various values of transition probability η. Solid lines: numerical network simulations (see Methods); dashed lines: analytical estimate (based on Eq (S3.23)); dashed gray line: maximal signal quality, which is the average Hamming distance between two random f_{out}-sparse patterns; dashed magenta line: retrieval threshold. Left: $f_{\text{in}} = 0.1$, right: $f_{\text{in}} = 0.012$. Other parameters: $N_{\text{in}} = N_{\text{out}} = 1000, f_{\text{out}} = 0.1, c_m = 1, c = 0.2$.

Signal quality with noisy input patterns. In this subsection, we evaluate the change of the signal quality when we apply noise to input patterns during retrieval (see Methods). The case without noise was already approximated in Eq (S3.23). If we include noise in this equation, the only term that changes there is the mean of the genuine distribution, which can be approximated by

$$\bar{\mu}_{g,\varepsilon}^{[P]} \approx m_g \rho_g(\lfloor f_{\text{out}}(P+1) \rfloor) + m_s \rho_n(\lfloor f_{\text{out}}(P+1) \rfloor) \tag{S3.24}$$

$$= m_g \left((c_m - c)\eta \left(1 - \frac{f_{\text{in}}\eta c_m}{c}\right)^{\lfloor f_{\text{out}}(P+1) \rfloor} + c \right) \tag{S3.25}$$

$$+ m_s \left(\frac{-f_{\text{in}}\eta(c_m - c)}{1 - f_{\text{in}}} \left(1 - \frac{f_{\text{in}}\eta c_m}{c}\right)^{\lfloor f_{\text{out}}(P+1) \rfloor} + c \right). \tag{S3.26}$$

The additional noise shifts the signal quality to lower values (compare Fig 6A top to Fig 6A bottom). Fig S3.5 compares the initial signal quality as a function of the transition probability η for several noise levels ϵ and, in addition, it shows the impact of other parameters on the initial signal quality.

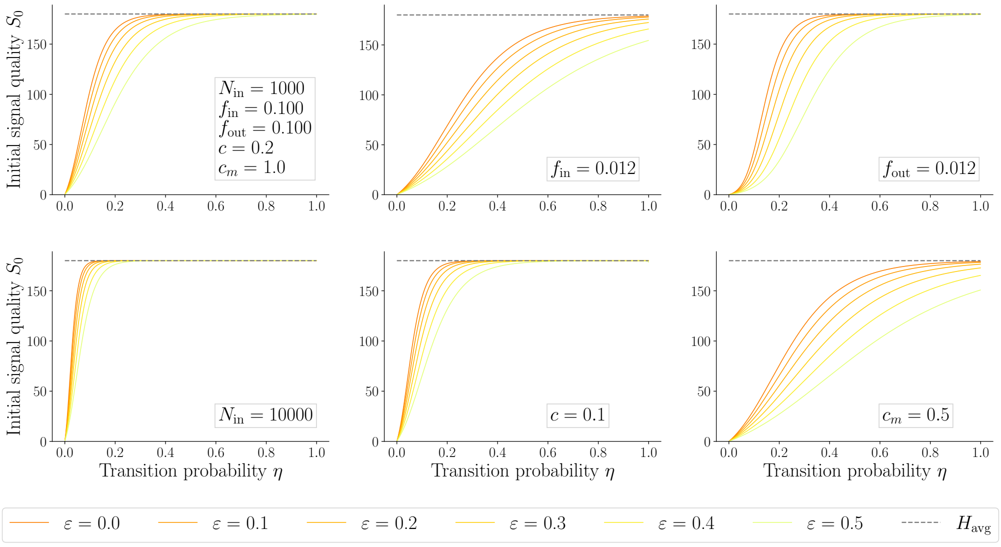

Fig S3.5. Effect of noise on the initial signal quality.
Comparison of initial signal quality based on Eq (S3.23) with $\mu_{g,\varepsilon}$ (Eq (S3.24)) for $P = 0$ as a function of the transition probability η for various noise levels ε. The initial signal quality is monotonically increasing as a function of the transition probability η. It approaches its maximal value H_{avg} more slowly if the noise level ε is increased. Default parameters (top left): $N_{\text{in}} = N_{\text{out}} = 1000, f_{\text{in}} = f_{\text{out}} = 0.1, c = 0.2, c_m = 1$. Top row: center – $f_{\text{in}} = 0.012$, right – $f_{\text{out}} = 0.012$. Bottom row: left – $N_{\text{in}} = 10^4$, center – $c = 0.1$, right – $c_m = 0.5$.

Given the signal quality without noise S_P (e.g. from numerical simulations), the corresponding signal quality with noisy input patterns during retrieval $S_{P,\varepsilon}$ can be estimated as

$$S_{P,\varepsilon} \approx S_P \cdot \frac{s_{P,\varepsilon} H_{\text{avg}}}{s_P H_{\text{avg}}} = S_P \cdot \frac{1 - \overline{s_{P,\varepsilon}}}{1 - \overline{s_P}}. \tag{S3.27}$$

The factor that relates the signal quality without noise S_P to the signal quality with noise $S_{P,\varepsilon}$ is thus given by

$$\frac{S_{P,\varepsilon}}{S_P} = \frac{1 - \frac{-1+\sqrt{1+4f_{\text{out}}(1-f_{\text{out}})\left(\exp\left(\frac{\pi}{\sqrt{3}}\frac{\bar{\mu}_{g,\varepsilon}^{[P]}-\mu_s}{\sigma_s}\right)-1\right)}}{2f_{\text{out}}(1-f_{\text{out}})\left(\exp\left(\frac{\pi}{\sqrt{3}}\frac{\bar{\mu}_{g,\varepsilon}^{[P]}-\mu_s}{\sigma_s}\right)-1\right)}}{1 - \frac{-1+\sqrt{1+4f_{\text{out}}(1-f_{\text{out}})\left(\exp\left(\frac{\pi}{\sqrt{3}}\frac{\bar{\mu}_{g}^{[P]}-\mu_s}{\sigma_s}\right)-1\right)}}{2f_{\text{out}}(1-f_{\text{out}})\left(\exp\left(\frac{\pi}{\sqrt{3}}\frac{\bar{\mu}_{g}^{[P]}-\mu_s}{\sigma_s}\right)-1\right)}}. \tag{S3.28}$$

Naturally, this factor is smaller for larger ε. For small transition probabilities, it is almost constant as a function of P (see solid lines with slope close to zero in Fig S3.6); however, for large transition probabilities, the factor is a non-monotonous function of P (dotted lines in Fig S3.6), and thus the maximal capacity and the optimal transition probability could exhibit a complex dependence on noise. Note that this analytical factor $S_{P,\varepsilon}/S_P$ is independent of H_{avg} and thus independent of N_{out}.

Fig S3.7 compares the signal quality $S_{P,\varepsilon}$ obtained from numerical simulations with noise $\varepsilon = 0.1$ (solid lines) to its approximation where the numerically obtained signal

quality S_P without noise is scaled by the analytical factor (dotted lines; see Eq (S3.28)). The factor Eq (S3.28) allows for a good approximation of the signal quality with noise based on the signal quality without noise.

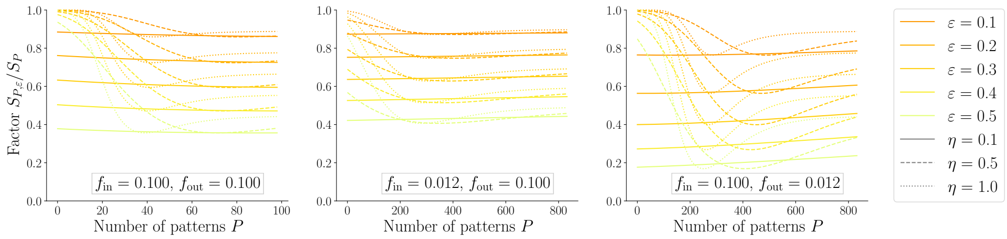

Fig S3.6. Factor relating S_P to $S_{P,\varepsilon}$.
Non-constant factor $S_{P,\varepsilon}/S_P$ that relates the signal quality without noise to the signal quality with noise level ε (cf. Eq (S3.28)) as a function of the number of patterns P for various noise levels $\varepsilon = 0.1, 0.2, 0.3, 0.4, 0.5$ (colors) and various transition probabilities $\eta = 0.1, 0.5, 1.0$ (solid, dashed and dotted). Default parameters (left): $N_{\text{in}} = N_{\text{out}} = 1000, f_{\text{in}} = f_{\text{out}} = 0.1, c = 0.2, c_m = 1$. Center: $f_{\text{in}} = 0.012$. Right: $f_{\text{out}} = 0.012$.

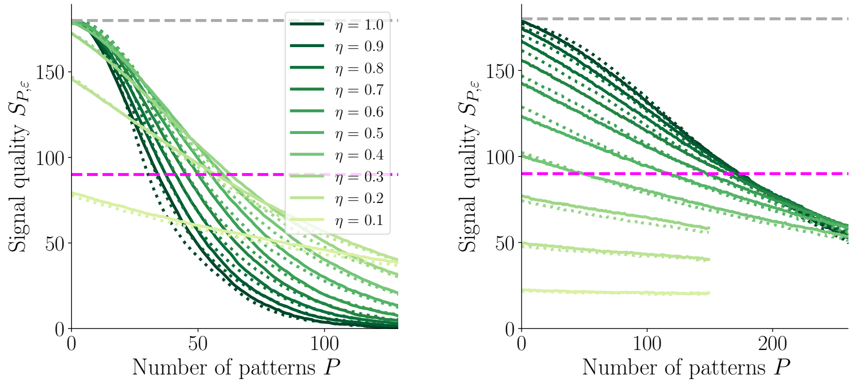

Fig S3.7. Approximation of $S_{P,\varepsilon}$ by scaling S_P.
Signal quality decays with number of subsequently learned patterns P for various values of transition probability η. Noise on input pattern during retrieval: $\varepsilon = 0.2$. The approximation of the signal quality $S_{P,\varepsilon}$ by Eq (S3.27), where S_P (without noise, $\varepsilon = 0$) is obtained from numerical simulations, fits the signal quality $S_{P,\varepsilon}$ obtained directly from numerical simulations with noise well. Solid lines - numerical simulations for $S_{P,\varepsilon}$ (see Methods); dotted lines - approximation of the signal quality $S_{P,\varepsilon}$ by Eq (S3.27) (with the factor Eq (S3.28)), where S_P (without noise, $\varepsilon = 0$) is obtained from numerical simulations (see Methods); dashed gray line - maximal signal quality which is the average Hamming distance between two random f_{out}-sparse patterns; dashed blue line - retrieval threshold. Left: $f_{\text{in}} = 0.1$, right: $f_{\text{in}} = 0.012$. (Other parameters: $N_{\text{in}} = N_{\text{out}} = 1000, f_{\text{out}} = 0.1, c_m = 1, c = 0.2$.)
