# Supplementary material for: Population sparseness determines strength of Hebbian plasticity for maximal memory lifetime in associative networks
Source: PLoS Comput Biol. 2026 Jul 6;22(7):e1013235. doi: 10.1371/journal.pcbi.1013235 (PMC13390959; doi:10.1371/journal.pcbi.1013235)
Supplement: S4 Appendix — Details regarding the derivation of the memory capacity for noise-less and noisy input patterns. (PDF) [file pcbi.1013235.s010.pdf]

S4 Appendix

Solution of the Capacity Equation

In order to solve the Capacity Equation (Eq (86) in the Methods)

$$F_s^{-1}\left(1 - \overline{t_S} f_{\text{out}}\right) = {F_g^{[P]}}^{-1}\left(\overline{t_S}(1 - f_{\text{out}})\right) \tag{S4.1}$$

for P, we use several approximation steps, which are outlined in this Appendix.

Approximation of $p_g^{[P]}$ by a single binomial distribution. In the following, we will approximate the distributions of dendritic sums such that the inverses of their distribution functions F_s and $F_g^{[P]}$ can be calculated. First, the linear combination of binomial PMFs in $p_g^{[P]}$ in Eq (11) is approximated by a single binomial PMF $\bar{p}_g^{[P]}$ (note the overline of the symbol p in the approximation).

A simple way to do so is by choosing the binomial distribution $\mathcal{B}_{M_{\text{in}},\,\rho_g(u)}$ that provides the largest contribution because it has the largest weight. The largest weight is $\binom{P}{u_{\text{mod}}} f_{\text{out}}^{u_{\text{mod}}}(1 - f_{\text{out}})^{P-u_{\text{mod}}}$ with $u_{\text{mod}} = \lfloor f_{\text{out}}(P+1)\rfloor$. We hence obtain the approximation

$$p_g^{[P]}(x) = \sum_{k=0}^{P}\binom{P}{k}{f_{\text{out}}}^k(1 - f_{\text{out}})^{P-k}\binom{M_{\text{in}}}{x}\rho_g(k)^x(1-\rho_g(k))^{M_{\text{in}}-x} \tag{S4.2}$$

$$\approx \binom{M_{\text{in}}}{x}\rho_g(u_{\text{mod}})^x(1-\rho_g(u_{\text{mod}}))^{M_{\text{in}}-x} =: \bar{p}_g^{[P]}(x). \tag{S4.3}$$

We are ultimately interested in a good approximation of the cumulative distribution function (see Capacity Equation (S4.1) and Fig 12 in the Methods). We denote the cumulative distribution function of the approximation (S4.3) as $\bar{F}_g^{[P]}$ (note, again, the overline). Fig S4.1 shows a numerical comparison of the summed probabilities

$$\sum_{x=0}^{X}\mathcal{P}(d_g^{[P]} = x) \tag{S4.4}$$

up to various values of X for the original distribution $p_g^{[P]}$ and the approximation $\bar{p}_g^{[P]}$ (Eq (S4.3)) in terms of the absolute difference between the two

$$\left|F_g^{[P]}(X) - \bar{F}_g^{[P]}(X)\right| \tag{S4.5}$$

and of the relative error

$$\frac{\left|F_g^{[P]}(X) - \bar{F}_g^{[P]}(X)\right|}{F_g^{[P]}(X)} \tag{S4.6}$$

as functions of P for several values of X between $M_{\text{in}}c$ and $M_{\text{in}}c_m$. The non-continuities in the errors as functions of P occur due to the discrete jumps in $u_{\text{mod}} = \lfloor f_{\text{out}}(P+1)\rfloor$. The approximation by an individual binomial distribution is better for smaller transition probabilities η (compare first to second and third to fourth row in Fig S4.1). As discussed in S2 Appendix, a large f_{in} (above all if combined with a transition probability η that is close to one) leads to a more pronounced multimodality of the distribution for small P values, which fades out more quickly with increasing P due to the steeper slope of $\rho_g(u)$ for small values of u (compare Fig S4.1 upper left to upper right). The more pronounced the multimodality, the less accurate the approximation by

a single binomial distribution can be. The output activation ratio f_{out} has a different effect than the input parameters on the quality of the approximation. Increasing f_{out} increases u_{mod}. A large u_{mod} allows for a low multimodality already for small values of P because the strongest weights are quickly in a range where $\rho_g(k)$ is not extremely steep anymore but approaching the asymptotic value c (compare Fig S4.1 upper left and upper center). We would like to emphasize that decreasing the morphological connectivity c_m strongly improves the match of $F_g^{[P]}$ and $\bar{F}_g^{[P]}$ (compare Fig S4.1 upper left to lower right). Most figures in this manuscript show results for $c_m = 1$. In terms of the approximation of the distributions of dendritic sums by a binomial distribution, this is the worst-case scenario. In biological nervous systems, there is typically no all-to-all morphological connectivity observed. The analytical approximation derived in this section hence improves significantly for more realistic values of $c_m \ll 1$.

For a detailed theoretical analysis of Willshaw-Palm distributions as in Eq (S4.2) see, for example, [1, 2].

Approximation of binomial distributions by normal distributions. In what follows, we approximate the binomial distributions p_s (Eq (**??**)) and $\bar{p}_g^{[P]}(x)$ (Eq (S4.3)) by normal distributions to be able to solve the Capacity Equation (S4.1) analytically. This assumption allows us to derive explicit expressions, which are excellent approximations for the regime of sparse input patterns but large input layer sizes and not too sparse connectivity. Similarly to the assumptions (in S3 Appendix) for the numerical solution of the Signal Quality Equation, we assume

$$M_{\mathrm{in}}c \gg 1,\ M_{\mathrm{in}}(1-c) \gg 1 \text{ and} \tag{S4.7}$$

$$M_{\mathrm{in}}\rho_g(\lfloor f_{\mathrm{out}}(P+1)\rfloor) \gg 1,\ M_{\mathrm{in}}(1-\rho_g(\lfloor f_{\mathrm{out}}(P+1)\rfloor)) \gg 1, \tag{S4.8}$$

for all P, which can be reduced to the two assumptions

$$M_{\mathrm{in}}c \gg 1 \text{ and } M_{\mathrm{in}}(1-\rho_g(\lfloor f_{\mathrm{out}}(P+1)\rfloor)) \gg 1 \tag{S4.9}$$

because $\rho_g(\lfloor f_{\mathrm{out}}(P+1)\rfloor) \geq c$. (Note that $M_{\mathrm{in}}(1-\rho_g(\lfloor f_{\mathrm{out}}(P+1)\rfloor)) \gg 1$ is not true for $\eta \approx 1, c_m \approx 1$ and small P.)

We approximate the binomial PMFs $p_s = \mathcal{B}(M_{\mathrm{in}}, c)$ and $\bar{p}_g^{[P]}$, as derived in the previous subsection, by normal probability densities

$$\tilde{p}_s = \mathcal{N}\left(\mu_s, \sigma_s^2\right) = \mathcal{N}\left(M_{\mathrm{in}}c, M_{\mathrm{in}}c(1-c)\right), \tag{S4.10}$$

$$\begin{aligned}\tilde{p}_g^{[P]} &= \mathcal{N}\left(\bar{\mu}_g^{[P]}, \bar{\sigma}_g^{[P]2}\right) \qquad \text{(S4.11)}\\ &= \mathcal{N}\left(M_{\mathrm{in}}\rho_g\left(\lfloor f_{\mathrm{out}}(P+1)\rfloor\right), M_{\mathrm{in}}\rho_g\left(\lfloor f_{\mathrm{out}}(P+1)\rfloor\right)\left(1-\rho_g\left(\lfloor f_{\mathrm{out}}(P+1)\rfloor\right)\right)\right),\end{aligned}$$

respectively, with cumulative distribution functions

$$\tilde{F}_s(x) = \frac{1}{2}\left(1+\operatorname{erf}\left(\frac{x-\mu_s}{\sigma_s\sqrt{2}}\right)\right) \text{ and} \tag{S4.12}$$

$$\tilde{F}_g^{[P]}(x) = \frac{1}{2}\left(1+\operatorname{erf}\left(\frac{x-\bar{\mu}_g^{[P]}}{\bar{\sigma}_g^{[P]}\sqrt{2}}\right)\right), \tag{S4.13}$$

respectively. Note that, in the following, we will often ignore the floor function in the probability density $\tilde{p}_g^{[P]}$ and use $\rho_g(f_{\mathrm{out}}P)$ instead of $\rho_g(\lfloor f_{\mathrm{out}}(P+1)\rfloor)$ because this simplifies some calculations and gives rise to a smooth dependence of the signal quality on P. This implies that the capacity can take any non-negative real value and not only integer values that can be interpreted as a number of patterns.

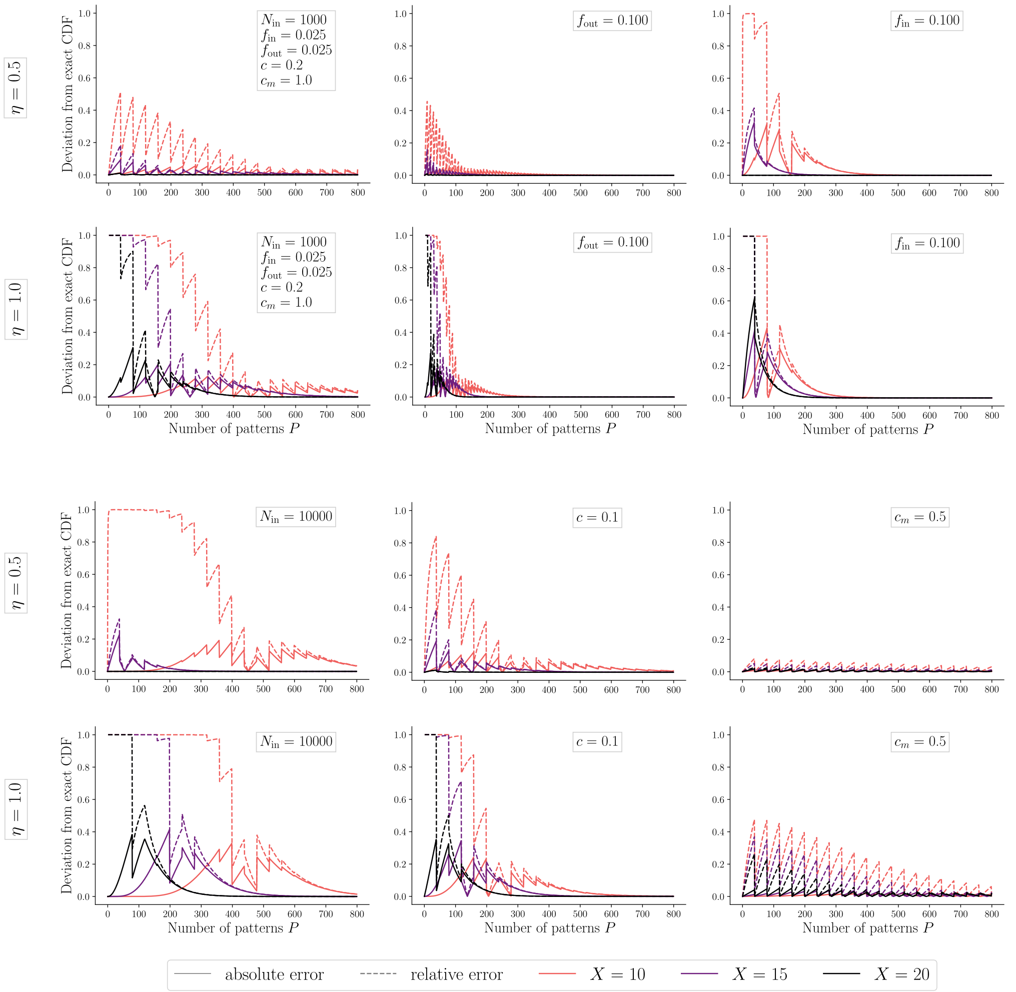

Fig S4.1. Error of approximation of $p_g^{[P]}$ by a single binomial distribution. Absolute error (solid lines, Eq (S4.5)) and relative error (dashed lines, Eq (S4.6)) of the approximation of $F_g^{[P]}$ by the cumulative distribution function $\bar{F}_g^{[P]}$ of a binomial distribution (Eq (S4.3)) for various parameter combinations. For three different values of X (argument of the CDF, color coded) between $cM_{\rm in}$ and $c_m M_{\rm in}$. In general, the errors decrease with an increase of X and with an increase of the number of patterns P. Default parameters: $N_{\rm in} = 1000$, $f_{\rm in} = f_{\rm out} = 0.025$, $c = 0.2$, $c_m = 1$ (upper left). First and third row: $\eta = 0.5$, second and fourth row: $\eta = 1$. Upper center: $f_{\rm out} = 0.1$, upper right: $f_{\rm in} = 0.1$, lower left: $N_{\rm in} = 10^4$, lower center: $c = 0.1$, lower right: $c_m = 0.5$.

The quantile functions in the Capacity Equation (S4.1) are hence approximated by

$$F_s^{-1}(x) \approx \mu_s + \sigma_s \sqrt{2}\, \mathrm{erf}^{-1}(2x-1)\,, \tag{S4.14}$$

$$F_g^{[P]^{-1}}(x) \approx \bar{\mu}_g^{[P]} + \bar{\sigma}_g^{[P]} \sqrt{2}\, \mathrm{erf}^{-1}(2x-1) \tag{S4.15}$$

and we obtain

$$F_s^{-1}\left(1-\overline{t_S}f_{\text{out}}\right) = F_g^{[P]^{-1}}\left(\overline{t_S}(1-f_{\text{out}})\right) \tag{S4.16}$$

$$\Leftrightarrow \quad \mu_s + \sigma_s\sqrt{2}\,\text{erf}^{-1}\left(2(1-\overline{t_S}f_{\text{out}})-1\right) = \bar{\mu}_g^{[P]} + \bar{\sigma}_g^{[P]}\sqrt{2}\,\text{erf}^{-1}\left(2(\overline{t_S}(1-f_{\text{out}})-1\right) \tag{S4.17}$$

$$\Leftrightarrow \quad \frac{\bar{\mu}_g^{[P]}-\mu_s}{\sqrt{2}\left(\sigma_s\cdot\text{erf}^{-1}\left(1-2\overline{t_S}f_{\text{out}}\right)-\bar{\sigma}_g^{[P]}\cdot\text{erf}^{-1}\left(2\overline{t_S}(1-f_{\text{out}})-1\right)\right)} = 1. \tag{S4.18}$$

We define the function

$$R(x,y) := \text{erf}^{-1}(1-2xy) \tag{S4.19}$$

and introduce the abbreviations

$$R_s := R(\overline{t_S}, f_{\text{out}}) = \text{erf}^{-1}\left(1-2\overline{t_S}f_{\text{out}}\right) \tag{S4.20}$$

and

$$R_g := R(\overline{t_S}, 1-f_{\text{out}}) = -\,\text{erf}^{-1}\left(2\overline{t_S}(1-f_{\text{out}})-1\right). \tag{S4.21}$$

They are called the weights of the standard deviations in Eq (S4.18) (Fig S4.2). Then we have

$$\frac{\bar{\mu}_g^{[P]}-\mu_s}{\sqrt{2}\left(\sigma_s\cdot R_s+\bar{\sigma}_g^{[P]}\cdot R_g\right)} = 1. \tag{S4.22}$$

Note that, for fixed $\overline{t_S}$, the weights' dependence on f_{out} is symmetric:

$$R_s\Big|_{f_{\text{out}}=f} = R_g\Big|_{f_{\text{out}}=1-f} \tag{S4.23}$$

For $f_{\text{out}} < 0.5$, the weight of the spurious standard deviation, R_s, is larger than the weight of the genuine distribution, R_g. For $\overline{t_S} \leq 0.5$, both R_g and R_s are non-negative for all f_{out}.

The left hand side of Eq (S4.22) can be interpreted as a generalized version of the sensitivity index

$$d' = \frac{|\mu_1-\mu_2|}{\frac{1}{2}(\sigma_1+\sigma_2)}, \tag{S4.24}$$

which can be used to quantify the discriminability of two distributions with means μ_1 and μ_2 and standard deviations σ_1 and σ_2. In our case, the additional weighting of the standard deviations by R_g and R_s takes into account the dissimilarly weighted contributions of the false positive and false negative activations due to the unequal numbers of genuine units, M_{out}, and spurious units, $N_{\text{out}}-M_{\text{out}}$, as reflected in the Balance Equation (see Eq (15) in the Methods).

Solving the Capacity Equation. With the approximations discussed in the previous subsections, the Capacity Equation (S4.1) is equivalent to Eq (S4.22). Now, we solve Eq (S4.22) for P with the means and standard deviations from the approximate distributions in Eqs (S4.10) and (S4.11), which we repeat here in a more

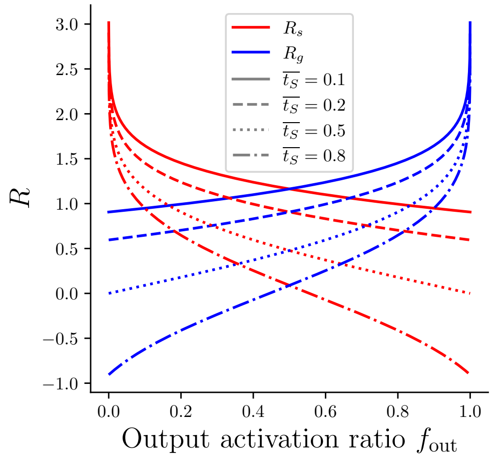

Fig S4.2. Weights of standard deviations.
Weights R_g and R_s as functions of f_{out} for $\overline{t_S} = 0.1, 0.2, 0.5$, and 0.8. R_g is R_s mirrored at $f_{\text{out}} = 0.5$. While R_s is decreasing as a function of increasing f_{out}, R_g is increasing as a function of increasing f_{out}. Both decrease with increasing $\overline{t_S}$. For $\overline{t_S} \leq 0.5$, R_g and R_s are non-negative. For $f_{\text{out}} \leq 0.5$, we have $R_s \geq 0$ and, for $f_{\text{out}} \geq 0.5$, we have $R_g \geq 0$.

explicit form for easier reference:

$$\mu_s = M_{\text{in}} c \tag{S4.25}$$

$$\sigma_s = \sqrt{M_{\text{in}} c(1-c)} \tag{S4.26}$$

$$\bar{\mu}_g^{[P]} = M_{\text{in}} \left[(c_m - c)\eta \left(1 - \frac{f_{\text{in}}\eta c_m}{c}\right)^{\lfloor f_{\text{out}}(P+1) \rfloor} + c \right] \tag{S4.27}$$

$$\bar{\sigma}_g^{[P]} = \sqrt{\begin{aligned} & M_{\text{in}} \left[(c_m - c)\eta \left(1 - \frac{f_{\text{in}}\eta c_m}{c}\right)^{\lfloor f_{\text{out}}(P+1) \rfloor} + c \right] \\ & \cdot \left[1 - (c_m - c)\eta \left(1 - \frac{f_{\text{in}}\eta c_m}{c}\right)^{\lfloor f_{\text{out}}(P+1) \rfloor} - c \right] \end{aligned}} . \tag{S4.28}$$

We obtain

$$\bar{\mu}_g^{[P]} - \mu_s = M_{\text{in}} (c_m - c)\eta \left(1 - \frac{f_{\text{in}}\eta c_m}{c}\right)^{\lfloor f_{\text{out}}(P+1) \rfloor} \tag{S4.29}$$

and

$$\sigma_s R_s + \bar{\sigma}_g^{[P]} R_g = \tag{S4.30}$$

$$\sqrt{M_{\text{in}}} \left(\sqrt{c(1-c)} R_s + \sqrt{\begin{aligned} & \left[(c_m - c)\eta \left(1 - \frac{f_{\text{in}}\eta c_m}{c}\right)^{\lfloor f_{\text{out}}(P+1) \rfloor} + c \right] \\ & \cdot \left[1 - (c_m - c)\eta \left(1 - \frac{f_{\text{in}}\eta c_m}{c}\right)^{\lfloor f_{\text{out}}(P+1) \rfloor} - c \right] \end{aligned}} R_g \right) .$$

Using the abbreviation $\alpha := (c_m - c)\eta \left(1 - \frac{f_{\text{in}}\eta c_m}{c}\right)^{\lfloor f_{\text{out}}(P+1)\rfloor}$, Eq (S4.22) becomes

$$\frac{\sqrt{M_{\text{in}}}\alpha}{\sqrt{2}\left(\sqrt{c(1-c)}R_s + \sqrt{[\alpha + c]\,[1-\alpha-c]}R_g\right)} = 1 \tag{S4.31}$$

$$\Leftrightarrow \sqrt{M_{\text{in}}}\alpha - \sqrt{c(1-c)}\sqrt{2}R_s = \sqrt{[\alpha + c]\,[1-\alpha-c]}\sqrt{2}R_g \tag{S4.32}$$

$$\begin{aligned}\Leftrightarrow M_{\text{in}}\alpha^2 - 2\sqrt{2M_{\text{in}}c(1-c)}R_s\alpha + 2c(1-c)R_s^2 \\ = 2\,[\alpha + c]\,[1-\alpha-c]\,R_g^2\end{aligned} \tag{S4.33}$$

$$\begin{aligned}\Leftrightarrow \alpha^2\left[M_{\text{in}} + 2R_g^2\right] + \alpha\left[2(2c-1)R_g^2 - 2\sqrt{2M_{\text{in}}c(1-c)}R_s\right] \\ + 2c(1-c)(R_s^2 - R_g^2) = 0.\end{aligned} \tag{S4.34}$$

Solving the quadratic equation gives

$$\alpha = \frac{2(1-2c)R_g^2 + 2\sqrt{2M_{\text{in}}c(1-c)}R_s \pm \sqrt{\begin{aligned}&\left[2(1-2c)R_g^2 + 2\sqrt{2M_{\text{in}}c(1-c)}R_s\right]^2 \\ &+8c(1-c)(M_{\text{in}} + 2R_g^2)(R_g^2 - R_s^2)\end{aligned}}}{2(M_{\text{in}} + 2R_g^2)}. \tag{S4.35}$$

For convenience, we introduce the abbreviations

$$\begin{aligned}A :=\ &(1-2c)R_g^2 + \sqrt{2M_{\text{in}}c(1-c)}R_s \\ &+ |R_g|\cdot\sqrt{R_g^2 + 2c(1-c)(M_{\text{in}} - 2R_s^2) + 2(1-2c)\sqrt{2M_{\text{in}}c(1-c)}R_s},\end{aligned} \tag{S4.36}$$

$$B := (c_m - c)(M_{\text{in}} + 2R_g^2) \tag{S4.37}$$

and have

$$\alpha = \frac{(c_m - c)A}{B}. \tag{S4.38}$$

The leading coefficient of the quadratic equation (S4.34) in α is positive. For increasing P, the term $(c_m - c)\eta\left(1 - \frac{f_{\text{in}}\eta c_m}{c}\right)^{\lfloor f_{\text{out}}(P+1)\rfloor}$ is decreasing. This means that, by increasing P, we are approaching zero from above and we are hence interested in the larger solution of the equation. Therefore, we have to choose the positive sign in Eq (S4.35). Since $c_m > c$, $\eta > 0$, and $\left(1 - \frac{f_{\text{in}}\eta c_m}{c}\right)^{\lfloor f_{\text{out}}(P+1)\rfloor} > 0$, α must be positive. Using the positive sign in front of the square root ensures a positive α. Note that the expression under the second square root could become negative if R_s becomes too large compared to R_g. For $M_{\text{in}} \gg 1$, this is only the case for very small f_{out} values combined with a small $\overline{t_S}$. These cases cannot be treated analytically in this way.

Next, we insert $(c_m - c)\eta\left(1 - \frac{f_{\text{in}}\eta c_m}{c}\right)^{f_{\text{out}}(P+1)}$ for α and approximate $\lfloor f_{\text{out}}(P+1)\rfloor \approx f_{\text{out}}P$ to obtain

$$f_{\text{out}}P\ln\left(1 - \frac{f_{\text{in}}\eta c_m}{c}\right) = \ln\left(\frac{A}{\eta B}\right) \tag{S4.39}$$

$$\Leftrightarrow \quad P = \frac{\ln\left(\frac{A}{\eta B}\right)}{\ln\left(1 - \frac{f_{\text{in}}\eta c_m}{c}\right)f_{\text{out}}}. \tag{S4.40}$$

Since P is a number of patterns, we round the obtained expression to the nearest integer and obtain the capacity of the network

$$P^* = \left\lfloor \frac{\ln\left(\frac{A}{\eta B}\right)}{\ln\left(1 - \frac{f_{\text{in}}\eta c_m}{c}\right) f_{\text{out}}} + \frac{1}{2} \right\rfloor . \tag{S4.41}$$

If R_g and R_s are known, this expression allows us to directly calculate the capacity of the network.

Approximation of the error function. At this point, the constants R_g and R_s (see Eqs (S4.20) and (S4.21)) could be evaluated numerically. Alternatively, to obtain an analytical expression of the capacity in terms of elementary functions, the constants R_s and R_g could be approximated with the help of any invertible approximation of the error function. We choose the highly accurate global Padé approximation

$$\text{erf}(x) \approx \text{sign}(x)\sqrt{1 - \exp\left(-x^2 \frac{\frac{4}{\pi} + ax^2}{1 + ax^2}\right)}, \tag{S4.42}$$

with $a = \frac{8(\pi-3)}{3\pi(4-\pi)}$ [3], see Fig S4.3, which allows for the approximation

$$\text{erf}^{-1}(x) \approx \text{sign}(x)\sqrt{\frac{-\frac{4}{\pi} - a\ln(1-x^2) + \sqrt{\left(\frac{4}{\pi} + a\ln(1-x^2)\right)^2 - 4a\ln(1-x^2)}}{2a}} \tag{S4.43}$$

to use for R_s and R_g in Eq (S4.22). All analytical approximations of the network capacity shown in figures are calculated with this approximation unless stated otherwise.

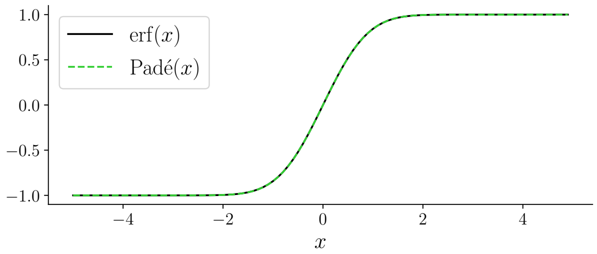

Fig S4.3. Approximation of the error function by a global Padé approximation. The maximal absolute error is 0.00033, the maximal relative error is 0.00035.

Extension to noisy input patterns during retrieval

In this section, we extend the derivation of the memory capacity and therewith the derivation of the maximal memory capacity and the optimal transition probability to the case of noisy input patterns during retrieval. We assume a noise level $\varepsilon \in (0, 1)$. See Section 'Noisy input patterns during retrieval' in the Methods for details on the distributions of dendritic sums with noisy cues; some of the expressions stated there are repeated in what follows to simplify the argument.

Mean and standard deviation. First, we approximate the distribution of dendritic sums of genuine output units

$$p_{g,\varepsilon}^{[P]}(x) = \sum_{u=0}^{P} \left[\binom{P}{u} {f_{\text{out}}}^{u} (1 - f_{\text{out}})^{P-u} \right. \tag{S4.44}$$

$$\left. \cdot \sum_{x_g=0}^{x} \left(\binom{m_g}{x_g} \rho_g(u)^{x_g} (1-\rho_g(u))^{m_g - x_g} \binom{m_s}{x_s} \rho_n(u)^{x_s} (1-\rho_n(u))^{m_s - x_s} \right) \right]$$

by the renormalized maximum summand ($u = u_{\text{mod}}$) and have

$$p_{g,\varepsilon}^{[P]}(x) \approx \bar{p}_{g,\varepsilon}^{[P]}(x) = \sum_{x_g=0}^{x} \left(\binom{m_g}{x_g} \rho_g(u_{\text{mod}})^{x_g} (1-\rho_g(u_{\text{mod}}))^{m_g - x_g} \right.$$

$$\left. \cdot \binom{m_s}{x_s} \rho_n(u_{\text{mod}})^{x_s} (1-\rho_n(u_{\text{mod}}))^{m_s - x_s} \right) \tag{S4.45}$$

with $u_{\text{mod}} = \lfloor (P+1) f_{\text{out}} \rfloor$.

We again use the abbreviation $\alpha := (c_m - c)\eta \left(1 - \frac{f_{\text{in}} \eta c_m}{c}\right)^{u_{\text{mod}}}$. As suggested by [4], the mean and standard deviation of $\bar{p}_{g,\varepsilon}^{[P]}$ are approximated as

$$\bar{\mu}_{g,\varepsilon}^{[P]} \approx m_g \rho_g(u_{\text{mod}}) + m_s \rho_n(u_{\text{mod}}) = m_g (\alpha + c) + m_s \left(\frac{-f_{\text{in}}}{1 - f_{\text{in}}} \alpha + c \right) \tag{S4.46}$$

$$= M_{\text{in}} \left[\alpha \left(1 - \varepsilon + \varepsilon \frac{-f_{\text{in}}}{1 - f_{\text{in}}} \right) + c \right] = M_{\text{in}} \left[\alpha \left(1 - \frac{\varepsilon}{1 - f_{\text{in}}} \right) + c \right] \tag{S4.47}$$

and

$$\bar{\sigma}_{g,\varepsilon}^{[P]} \approx \sqrt{m_g \rho_g(u_{\text{mod}})(1 - \rho_g(u_{\text{mod}})) + m_s \rho_n(u_{\text{mod}})(1 - \rho_n(u_{\text{mod}}))} \tag{S4.48}$$

$$= \sqrt{M_{\text{in}} \left[(1-2c)\alpha \left(1 - \frac{\varepsilon}{1 - f_{\text{in}}} \right) - \alpha^2 \left(1 - \frac{\varepsilon(1 - 2f_{\text{in}})}{(1 - f_{\text{in}})^2} \right) + c(1-c) \right]}. \tag{S4.49}$$

Memory capacity of the network. As for the case without noise, we solve Eq (S4.22) for P to obtain the capacity of the network. With $\bar{\mu}_{g,\varepsilon}^{[P]}$ and $\bar{\sigma}_{g,\varepsilon}^{[P]}$, I have

$$\frac{M_{\text{in}} \left[\alpha \left(1 - \frac{\varepsilon}{1 - f_{\text{in}}} \right) + c \right] - M_{\text{in}} c}{\sqrt{2} \left(R_g \sqrt{ M_{\text{in}} \left[(1-2c)\alpha \left(1 - \frac{\varepsilon}{1 - f_{\text{in}}} \right) - \alpha^2 \left(1 - \frac{\varepsilon(1 - 2f_{\text{in}})}{(1 - f_{\text{in}})^2} \right) + c(1-c) \right] } + R_s \sqrt{M_{\text{in}} c(1-c)} \right)} = 1 \tag{S4.50}$$

$$\Leftrightarrow \alpha^2 \left[M_{\text{in}} \left(1 - \frac{\varepsilon}{1 - f_{\text{in}}} \right)^2 + 2R_g^2 \left(1 - \frac{\varepsilon(1 - 2f_{\text{in}})}{(1 - f_{\text{in}})^2} \right) \right]$$

$$- \alpha 2 \left(1 - \frac{\varepsilon}{1 - f_{\text{in}}} \right) \left[\sqrt{2 M_{\text{in}} c(1-c)} R_s + (1-2c) R_g^2 \right]$$

$$+ 2c(1-c)(R_s^2 - R_g^2) = 0 \tag{S4.51}$$

$$\Leftrightarrow \alpha = \frac{(c_m - c) A_\varepsilon}{B_\varepsilon}, \tag{S4.52}$$

with the abbreviations

$$A_\varepsilon := \left((1-2c)R_g^2 + \sqrt{2M_{\rm in}c(1-c)}R_s\right)\left(1-\frac{\varepsilon}{1-f_{\rm in}}\right) + |R_g| \\ \cdot\sqrt{\begin{array}{l}\left(2\sqrt{2M_{\rm in}c(1-c)}(1-2c)R_s + (1-2c)^2R_g^2 + 2c(1-c)M_{\rm in}\right) \\ \cdot\left(1-\frac{\varepsilon}{1-f_{\rm in}}\right)^2 - 4c(1-c)(R_s^2-R_g^2)\left(1-\frac{\varepsilon(1-2f_{\rm in})}{(1-f_{\rm in})^2}\right)\end{array}} \tag{S4.53}$$

and

$$B_\varepsilon := (c_m - c)\left[M_{\rm in}\left(1-\frac{\varepsilon}{1-f_{\rm in}}\right)^2 + 2R_g^2\left(1-\frac{\varepsilon(1-2f_{\rm in})}{(1-f_{\rm in})^2}\right)\right]. \tag{S4.54}$$

These results generalize the expressions for A and B in Eqs (S4.36) and (S4.37), which were derived for $\varepsilon = 0$. Expanding $\ln(1-x)$, we obtain the capacity

$$P_\varepsilon^* = \left\lfloor \frac{\ln\left(\frac{A_\varepsilon}{\eta B_\varepsilon}\right)}{\ln\left(1-\frac{f_{\rm in}\eta c_m}{c}\right)f_{\rm out}} + \frac{1}{2}\right\rfloor \approx \frac{c}{-f_{\rm in}f_{\rm out}\eta c_m}\ln\left(\frac{A_\varepsilon}{\eta B_\varepsilon}\right). \tag{S4.55}$$

Approximation of $\bar{p}_{g,\varepsilon}^{[P]}$ by a single binomial distribution. In order to find a relationship between the capacity P_ε^* with noise and the capacity P^* without noise that is easier to interpret, we can further approximate $\bar{p}_{g,\varepsilon}^{[P]}$ by a single binomial distribution $\bar{p}_{g,\varepsilon}^{[P],\rm approx}$ with the same mean

$$\bar{\mu}_{g,\varepsilon}^{[P]} = M_{\rm in}\left[\alpha\left(1-\frac{\varepsilon}{1-f_{\rm in}}\right) + c\right] \tag{S4.56}$$

as in Eq (S4.46). If $\bar{\mu}_{g,\varepsilon}^{[P]}$ is interpreted as the mean of a binomial distribution $\mathcal{B}_{M_{\rm in},p}$, its success probability is $p = \alpha\left(1-\frac{\varepsilon}{1-f_{\rm in}}\right) + c$ and the corresponding effective standard deviation has to be

$$\bar{\sigma}_{g,\varepsilon}^{[P],\rm approx} = \sqrt{M_{\rm in}\left[\alpha\left(1-\frac{\varepsilon}{1-f_{\rm in}}\right)+c\right]\cdot\left[1-\alpha\left(1-\frac{\varepsilon}{1-f_{\rm in}}\right)-c\right]}. \tag{S4.57}$$

This approximation is exact if the two variances

$$m_g\rho_g(u_{\rm mod})(1-\rho_g(u_{\rm mod})) \qquad \text{and} \tag{S4.58}$$

$$m_s\rho_n(u_{\rm mod})(1-\rho_n(u_{\rm mod})) \tag{S4.59}$$

that contribute to $\bar{\sigma}_{g,\varepsilon}^{[P]}$ (Eq (S4.48)) are the same. Inserting $\bar{\mu}_{g,\varepsilon}^{[P]}$ and $\bar{\sigma}_{g,\varepsilon}^{[P],\rm approx}$ in Eq (S4.22) and using the abbreviation $\tilde{\alpha} := \alpha\cdot\left(1-\frac{\varepsilon}{1-f_{\rm in}}\right)$ yields the equation

$$\frac{M_{\rm in}\left(\tilde{\alpha}+c\right) - M_{\rm in}c}{\sqrt{2}\left(R_g\sqrt{2\left[(1-2c)\tilde{\alpha}-\tilde{\alpha}^2+c(1-c)\right]} + R_s\sqrt{c(1-c)}\right)} = 1. \tag{S4.60}$$

This equation in $\tilde{\alpha}$ is identical with Eq (S4.31) in α and thus has the solution

$$\tilde{\alpha} = \frac{(c_m-c)A}{B}, \tag{S4.61}$$

with A and B being exactly the same as without noisy input patterns (Eqs (S4.36) and (S4.37)). From this we obtain the capacity

$$P_\varepsilon^{*\text{approx}} = \left\lfloor \frac{\ln\left(\frac{A}{\eta\left(1-\frac{\varepsilon}{1-f_{\text{in}}}\right)B}\right)}{\ln\left(1-\frac{f_{\text{in}}\eta c_m}{c}\right)f_{\text{out}}} + \frac{1}{2} \right\rfloor \tag{S4.62}$$

$$\approx \frac{c}{-f_{\text{in}}f_{\text{out}}\eta c_m}\left[\ln\left(\frac{A}{\eta B}\right) - \ln\left(1-\frac{\varepsilon}{1-f_{\text{in}}}\right)\right] \tag{S4.63}$$

$$= P^* + \frac{\ln\left(1-\frac{\varepsilon}{1-f_{\text{in}}}\right)c}{f_{\text{in}}f_{\text{out}}\eta c_m}. \tag{S4.64}$$

References

1. Knoblauch A. Statistical implications of clipped Hebbian learning of cell assemblies. Neurocomputing. 2005;65:647–652.

2. Knoblauch A. Neural associative memory and the Willshaw–Palm probability distribution. SIAM Journal on Applied Mathematics. 2008;69(1):169–196.

3. Winitzki S. Uniform approximations for transcendental functions. In: Computational Science and Its Applications—ICCSA 2003: International Conference Montreal, Canada, May 18–21, 2003 Proceedings, Part I 3. Springer; 2003. p. 780–789.

4. Buckingham JT. Delicate Nets, Faint Recollections: A Study of Partially Connected Associative Network Memories [PhD thesis]. University of Edinburgh; 1991.
