# Supplementary material for: Population sparseness determines strength of Hebbian plasticity for maximal memory lifetime in associative networks
Source: PLoS Comput Biol. 2026 Jul 6;22(7):e1013235. doi: 10.1371/journal.pcbi.1013235 (PMC13390959; doi:10.1371/journal.pcbi.1013235)
Supplement: S5 Appendix — Showing monotonic increase of capacity for input activation ratios close to zero. (PDF) [file pcbi.1013235.s011.pdf]

S5 Appendix

Monotonicity of capacity for small f_{in}

In this section, we show that the capacity as a function of the input activation ratio f_{in} is monotonically increasing for f_{in} close to zero. The generalized sensitivity index

$$d'_R = \frac{\bar{\mu}_g^{[P]} - \mu_s}{\sqrt{2}\left(\sigma_s R_s + \bar{\sigma}_g^{[P]} R_g\right)} \tag{S5.1}$$

(introduced in Eq (91) of the manuscript) is decreasing with the number of patterns P. The memory capacity of the network is the number of patterns P for which the equation

$$d'_R = 1 \tag{S5.2}$$

is fulfilled. In the following, we show that, for fixed P and small f_{in}, d'_R is monotonically increasing as a function of f_{in}. Hence, the smaller f_{in}, the smaller the P for which Eq (S5.2) is fulfilled and the smaller the capacity.

For small values of f_{in}, we use the Taylor expansion of $\rho_g(u_{\text{mod}})$, where $u_{\text{mod}} = \lfloor f_{\text{out}}(P+1) \rfloor$, as a function of f_{in} at $f_{\text{in}} = 0$

$$\rho_g(u_{\text{mod}}) = (c_m - c)\eta \left(1 - f_{\text{in}} \frac{\eta c_m}{c}\right)^{u_{\text{mod}}} + c \tag{S5.3}$$

$$= (c_m - c)\eta \left(1 - f_{\text{in}} \frac{\eta c_m u_{\text{mod}}}{c} + \mathcal{O}(f_{\text{in}}^2)\right) + c \tag{S5.4}$$

and obtain the Taylor expanded versions of the means and standard deviations of the distributions of dendritic sums:

$$\mu_s = f_{\text{in}} N_{\text{in}} c \tag{S5.5}$$

$$\sigma_s = \sqrt{f_{\text{in}} N_{\text{in}} c(1-c)} \tag{S5.6}$$

$$\bar{\mu}_g^{[P]} = f_{\text{in}} N_{\text{in}} \left[(c_m - c)\eta \left(1 - f_{\text{in}} \frac{\eta c_m u_{\text{mod}}}{c} + \mathcal{O}(f_{\text{in}}^2)\right) + c\right] \tag{S5.7}$$

$$\bar{\sigma}_g^{[P]} = \sqrt{\begin{aligned} & f_{\text{in}} N_{\text{in}} \left[(c_m - c)\eta \left(1 - f_{\text{in}} \frac{\eta c_m u_{\text{mod}}}{c} + \mathcal{O}(f_{\text{in}}^2)\right) + c\right] \\ & \cdot \left[1 - (c_m - c)\eta \left(1 - f_{\text{in}} \frac{\eta c_m u_{\text{mod}}}{c} + \mathcal{O}(f_{\text{in}}^2)\right) - c\right]. \end{aligned}} \tag{S5.8}$$

In order to show that d'_R is monotonically increasing with f_{in}, we show that

$$f_1 < f_2 \Rightarrow d'_R(f_1) \leq d'_R(f_2). \tag{S5.9}$$

We have

$$\frac{\bar{\mu}_g^{[P]} - \mu_s}{\sqrt{2}\left(\sigma_s R_s + \bar{\sigma}_g^{[P]} R_g\right)}(f_1) \leq \frac{\bar{\mu}_g^{[P]} - \mu_s}{\sqrt{2}\left(\sigma_s R_s + \bar{\sigma}_g^{[P]} R_g\right)}(f_2) \tag{S5.10}$$

$$\Leftrightarrow \sqrt{f_1}\left(1 - f_1 \frac{\eta c_m u_{\text{mod}}}{c} + \mathcal{O}(f_1^2)\right) \cdot \left[R_s \sqrt{c(1-c)} + R_g \sqrt{a} \sqrt{1 + f_2 \frac{b}{a} + \mathcal{O}(f_2^2)}\right] \tag{S5.11}$$

$$\leq \sqrt{f_2}\left(1 - f_2 \frac{\eta c_m u_{\text{mod}}}{c} + \mathcal{O}(f_2^2)\right) \cdot \left[R_s \sqrt{c(1-c)} + R_g \sqrt{a} \sqrt{1 + f_1 \frac{b}{a} + \mathcal{O}(f_1^2)}\right]$$

with $a := c(1-c) + (c_m - c)\eta(1 - 2c - (c_m - c)\eta)$ and $b := (c_m - c)\eta\frac{\eta c_m u_\text{mod}}{c}(2(c_m - c)\eta - 1 + 2c)$. Note that $a > 0$ only if η and c_m are not both 1. We exclude the case $c_m = 1$, $\eta = 1$ here. However, evaluating Eq (92) (in the main text) for small f_in values suggests that the monotonicity of the capacity as a function of f_in is maintained also for $c_m = 1$, $\eta = 1$.

We know that $\bar{\mu}_g^{[P]} - \mu_s \geq 0$ and since we are interested in $d'_R \geq 0$, we can further assume that $\sigma_s R_s + \bar{\sigma}_g^{[P]} R_g \geq 0$. Both sides of the inequality are thus positive and we can square them to obtain

$$f_1\left(1 - 2f_1\frac{\eta c_m u_\text{mod}}{c} + \mathcal{O}(f_1^2)\right) \tag{S5.12}$$
$$\cdot\left[R_s^2 c(1-c) + 2R_s\sqrt{c(1-c)}R_g\sqrt{a}\sqrt{1 + f_2\frac{b}{a} + \mathcal{O}(f_2^2)} + R_g^2 a\left(1 + f_2\frac{b}{a} + \mathcal{O}(f_2^2)\right)\right]$$
$$\leq f_2\left(1 - 2f_2\frac{\eta c_m u_\text{mod}}{c} + \mathcal{O}(f_2^2)\right) \tag{S5.13}$$
$$\cdot\left[R_s^2 c(1-c) + 2R_s\sqrt{c(1-c)}R_g\sqrt{a}\sqrt{1 + f_1\frac{b}{a} + \mathcal{O}(f_1^2)} + R_g^2 a\left(1 + f_1\frac{b}{a} + \mathcal{O}(f_2^2)\right)\right].$$

We now use the Taylor expansion

$$\sqrt{1 + x\alpha} = 1 + x\frac{\alpha}{2} + \mathcal{O}(x^2) \tag{S5.14}$$

at $x = 0$ and obtain

$$f_1\left(1 - 2f_1\frac{\eta c_m u_\text{mod}}{c} + \mathcal{O}(f_1^2)\right) \tag{S5.15}$$
$$\cdot\left[\left(R_s\sqrt{c(1-c)} + R_g\sqrt{a}\right)^2 + f_2\frac{b}{\sqrt{a}}R_g\left(R_g\sqrt{a} + R_s\sqrt{c(1-c)}\right) + \mathcal{O}(f_2^2)\right]$$
$$\leq f_2\left(1 - 2f_2\frac{\eta c_m u_\text{mod}}{c} + \mathcal{O}(f_2^2)\right) \tag{S5.16}$$
$$\cdot\left[\left(R_s\sqrt{c(1-c)} + R_g\sqrt{a}\right)^2 + f_1\frac{b}{\sqrt{a}}R_g\left(R_g\sqrt{a} + R_s\sqrt{c(1-c)}\right) + \mathcal{O}(f_1^2)\right].$$

Keeping only the terms up to the first order yields

$$f_1\left(R_s\sqrt{c(1-c)} + R_g\sqrt{a}\right)^2 \leq f_2\left(R_s\sqrt{c(1-c)} + R_g\sqrt{a}\right)^2. \tag{S5.17}$$

Since $\left(R_s\sqrt{c(1-c)} + R_g\sqrt{a}\right)^2 \geq 0$, this inequality is true for $f_1 < f_2$ and we have thus shown that the capacity is a monotonically increasing function of f_in for values of f_in close to zero.
