# Supplementary material for: Population sparseness determines strength of Hebbian plasticity for maximal memory lifetime in associative networks
Source: PLoS Comput Biol. 2026 Jul 6;22(7):e1013235. doi: 10.1371/journal.pcbi.1013235 (PMC13390959; doi:10.1371/journal.pcbi.1013235)
Supplement: S6 Appendix — Optimal transition probability and maximal capacity for fixed number of functional connections per input neuron and fixed number of functional connections averaged across the whole network. (PDF) [file pcbi.1013235.s012.pdf]

S6 Appendix

Comparison to other versions of homeostasis

The homeostatic mechanism in this manuscript normalizes the number of functional connections per output unit, i.e., the in-degree, by silencing connections that originate from an inactive input unit. In the following, we compare this method to two other versions of synaptic homeostasis.

Instead of normalizing the in-degree, the number of functional connections per input unit could be preserved. This version simply inverses the roles of input and output units in the homeostasis step: Among the genuine-spurious connections, we randomly silence as many as necessary to maintain the connectivity c per input unit. In this case, we also normalize the morphological connectivity c_m per input unit and not per output unit. Fig S6.1A and B show the maximal capacity P^*_{max} and the optimal transition probability η_{opt} obtained from numerical simulations with this method.

Alternatively, the functional connectivity c (and the morphological connectivity c_m) could only be maintained as an average across the whole network. In this instance, the appropriate number of randomly chosen spurious-spurious connections are silenced. The maximal capacity P^*_{max} and the optimal transition probability η_{opt} for random deactivation are depicted in Fig S6.1C and D.

In both cases, the results remain qualitatively unchanged compared to the results presented in the manuscript: The maximal capacity P^*_{max} decreases as a function of the output activation ratio f_{out} but depends non-monotonically on the input activation ratio f_{in}. The optimal transition probability η_{opt} decreases with both f_{in} and f_{out} but the effect due to f_{in} is stronger (compare Fig S6.1 to Fig 3D,E).

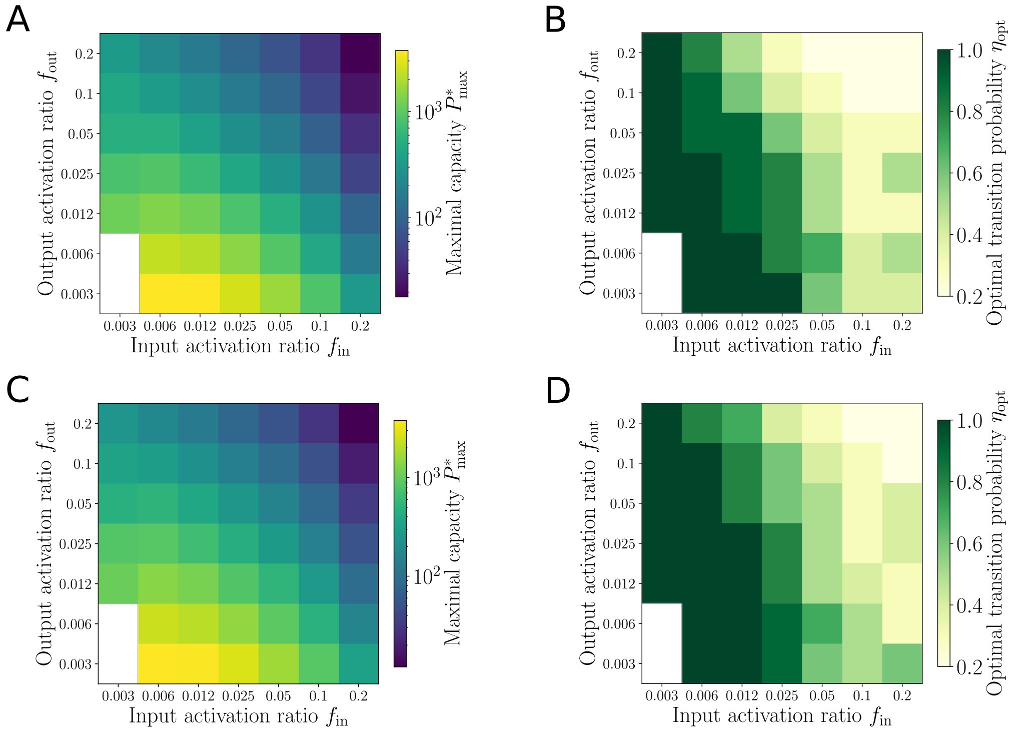

Fig S6.1. Maximal capacity and optimal transition probability with other versions of homeostasis.
The effect of f_{in} and f_{out} on the memory capacity P^*_{max} and on the optimal transition η_{opt} is qualitatively preserved with other homeostasis mechanisms. In (**A**)-(**B**), the functional connectivity c is preserved per input unit. In (**C**)-(**D**), c is maintained across the whole network. In both (**A**)-(**B**) and (**C**)-(**D**), the memory capacity P^*_{max} monotonically decreases with f_{out} whereas P^*_{max} depends non-monotonically on f_{in}. The optimal transition probability η_{opt} decreases with increasing f_{in} and f_{out}. Compare (**A**) and (**C**) to Fig 3D as well as (**B**) and (**D**) to Fig 3E. In (**A**)-(**D**), $N_{\mathrm{in}} = N_{\mathrm{out}} = 1000, c = 0.2, c_m = 1, t_S = 0.5, N_{\mathrm{avg}} = 200$.
