# Supplementary material for: Population sparseness determines strength of Hebbian plasticity for maximal memory lifetime in associative networks
Source: PLoS Comput Biol. 2026 Jul 6;22(7):e1013235. doi: 10.1371/journal.pcbi.1013235 (PMC13390959; doi:10.1371/journal.pcbi.1013235)
Supplement: S7 Appendix — Derivation of an upper bound for the error in activating the right number of output units and discussion of practical solutions for when the exact output activation ratio cannot be achieved. (PDF) [file pcbi.1013235.s013.pdf]

S7 Appendix

Can the output activation ratio be enforced?

During retrieval, since the distributions of dendritic sums are discrete distributions, it is not necessarily possible (or rather usually impossible) to choose an activation threshold such that exactly a number M_{out} of output units is activated and exactly a number $N_{\text{out}} - M_{\text{out}}$ remains inactive. As both the input states and the synaptic states are binary, it is likely that several output units have the same dendritic sum. In particular, this happens if M_{in} and η are small because they limit the range of different values that the dendritic sums can take. Fig S7.1A shows an exemplary histogram of dendritic sums, where the activation threshold T_{in} cannot be chosen such that exactly the desired number of active output units $M_{\text{out}} = 100$ is activated. We call the dendritic sums d and we define the error

$$M_{\text{err}} := \min_{T_{\text{in}}} \left| M_{\text{out}} - \sum_{\{d \geq T_{\text{in}}\}} 1 \right|, \tag{S7.1}$$

which is the smallest deviation from the exact M_{out} that can be achieved with the best choice of activation threshold T_{in}. In Fig S7.1B, we see that the mean error (normalized by M_{out}) decreases with increasing M_{in} and increasing η.

In this section, we first provide a theoretical upper bound of the error M_{err} and compare this estimate to numerical averages. Then, we discuss possible practical ways of dealing with the issue of achieving a given output sparseness in numerical simulations.

Note that these considerations become unnecessary if we approximate the discrete distributions of dendritic sums by continuous normal distributions as in the analytical parts of the Methods Section of this paper.

Theoretical analysis

In the following, we derive an upper bound for the error M_{err} in activating the right number of output units. We first discuss the case $P = 0$, i.e., retrieval immediately after learning a particular pattern, without additional patterns learned in between. The maximal error M_{err} depends on how many additional units are activated (or deactivated) if the threshold is shifted to the left (or to the right) by 1. The smaller the values of the PMF, the smaller the error we potentially have to make. First, assuming $M_{\text{in}}(1 - \rho_g(0)) \gg 1$ and $M_{\text{in}}c \gg 1$, we approximate the distributions of the dendritic sums of genuine and spurious output units by normal distributions

$$\mathcal{N}\left(\mu_g^{[0]}, {\sigma_g^{[0]}}^2\right) = \mathcal{N}\left(M_{\text{in}}(c + (c_m - c)\eta), M_{\text{in}}(c + (c_m - c)\eta)(1 - c - (c_m - c)\eta)\right) \tag{S7.2}$$

and

$$\mathcal{N}\left(\mu_s, \sigma_s^2\right) = \mathcal{N}\left(M_{\text{in}}c, M_{\text{in}}c(1 - c)\right), \tag{S7.3}$$

respectively. We first consider the genuine distribution and derive an upper bound for the error by focusing on the largest value of the probability density function (PDF) which is at $x = \mu_g^{[0]}$:

$$\mathcal{P}(d_g = \mu_g^{[0]}) \approx \mathcal{N}\left(\mu_g^{[0]}, {\sigma_g^{[0]}}^2\right)(\mu_g^{[0]}) \tag{S7.4}$$

$$= \frac{1}{\sigma_g^{[0]}\sqrt{2\pi}} \exp\left(-\frac{1}{2}\left(\frac{\mu_g^{[0]} - \mu_g^{[0]}}{\sigma_g^{[0]}}\right)^2\right) = \frac{1}{\sigma_g^{[0]}\sqrt{2\pi}}. \tag{S7.5}$$

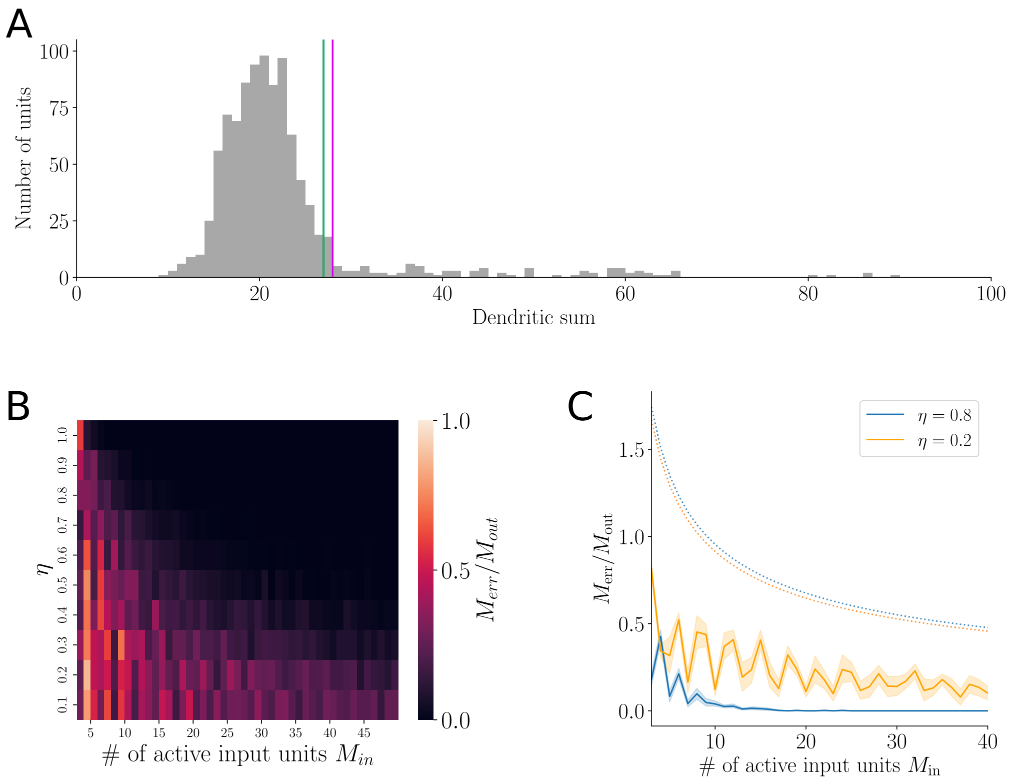

Fig S7.1. Error in number of active output units.
(**A**) In this histogram of dendritic sums (genuine and spurious together), there is no activation threshold $T_{\rm in}$ that could activate exactly $M_{\rm out} = 100$ units. If the magenta threshold ($T_{\rm in} = 28$) is chosen, only 97 units are activated. If the threshold is reduced by one and chosen at the green line ($T_{\rm in} = 27$), 119 units are activated. In (**A**), $P = 25, \eta = 0.8, N_{\rm in} = N_{\rm out} = 1000, f_{\rm in} = f_{\rm out} = 0.1, c = 0.2, c_m = 1$. (**B**) The normalized error $M_{\rm err}/M_{\rm out}$ decreases as a function of $M_{\rm in}$ and as a function of η. (**C**) Comparison between theoretical upper bound of the error (dotted lines, Eq (S7.17)) and numerically calculated error (solid lines) for $\eta = 0.2$ (orange) and $\eta = 0.8$ (blue). In (**B**) - (**C**), $P = 0, N_{\rm in} = N_{\rm out} = 1000, M_{\rm out} = 48, c = 0.2, c_m = 1$.

The number of genuine units is $M_{\rm out}$, thus this value needs to be scaled by $M_{\rm out}$ to describe the average number of genuine units that have a dendritic sum of $\mu_g^{[0]}$:

$$\frac{M_{\rm out}}{\sqrt{M_{\rm in}(c + (c_m - c)\eta)(1 - c - (c_m - c)\eta)}\sqrt{2\pi}}. \tag{S7.6}$$

For the spurious distribution, we do the same and obtain the largest value of the PDF

$$\mathcal{P}(d_s = \mu_s) \approx \frac{1}{\sigma_s\sqrt{2\pi}}, \tag{S7.7}$$

which has to be scaled by $N_{\rm out} - M_{\rm out}$:

$$\frac{N_{\rm out} - M_{\rm out}}{\sqrt{M_{\rm in}c(1 - c)}\sqrt{2\pi}}. \tag{S7.8}$$

The total change in number of activated units if the threshold is shifted by 1 is the sum of Eqs (S7.6) and (S7.8). At worst, $M_{\rm err}$ is half of this sum because if $M_{\rm err}$ was larger,

the activation threshold would not have been chosen optimally:

$$M_{\text{err}} \leq \frac{1}{2}\left(\frac{M_{\text{out}}}{\sqrt{M_{\text{in}}(c+(c_m-c)\eta)(1-c-(c_m-c)\eta)}\sqrt{2\pi}} + \frac{N_{\text{out}}-M_{\text{out}}}{\sqrt{M_{\text{in}}c(1-c)}\sqrt{2\pi}}\right). \tag{S7.9}$$

For $M_{\text{out}} \ll N_{\text{out}}$, this upper bound can be approximated by

$$M_{\text{err}} \leq \frac{1}{2}\left(\frac{N_{\text{out}}}{\sqrt{M_{\text{in}}c(1-c)}\sqrt{2\pi}}\right). \tag{S7.10}$$

It turns out that this simple approximation, which does not depend on η often is appropriate even independent of the condition $M_{\text{out}} \ll N_{\text{out}}$: For small values of η and a small enough functional connectivity c, we have

$$\frac{1}{\sqrt{M_{\text{in}}(c+(c_m-c)\eta)(1-c-(c_m-c)\eta)}} \leq \frac{1}{\sqrt{M_{\text{in}}c(1-c)}}, \tag{S7.11}$$

which is sharper for c_m small and thus close to c but even for $c_m = 1$ and e.g. $\eta \leq 0.5$ it is true for any $c < \frac{1}{3}$.

For $0 < P \ll P^*$, the approximation of the distribution of the dendritic sums of genuine units by a normal distribution can be less accurate (in particular for large η) and the above analysis cannot easily be generalized. Nevertheless, we know that with increasing P the distribution first becomes wider (σ_g is increasing) while the distribution of the dendritic sums of the spurious units does not change with P, which decreases the contribution of the genuine units (first term in Eq (S7.9)) to the change in the number of activated units due to small shifts of the activation threshold. In this case, Eq (S7.10) is thus also a suitable approximate upper bound. After becoming wider, the distribution of the dendritic sums of the genuine units approaches the distribution of the dendritic sums of the spurious units, which means $1/\sqrt{M_{\text{in}}(c+(c_m-c)\eta)(1-c-(c_m-c)\eta)} \approx 1/\sqrt{M_{\text{in}}c(1-c)}$. For large P, we can thus again make use of the same upper bound (S7.10).

For $P = 0$ and η close to one, the inequality (S7.11) that we used to simplify condition (S7.9) to (S7.10) is (especially for large c_m) usually not fulfilled. (In the limit of $\eta = 1$ and $c_m = 1$, we even have $\sigma_g = 0$ because $\mathcal{P}\left(d_g = M_{\text{in}}\right) = 1$.) However, in these cases, the distributions of dendritic sums of genuine units and spurious units are very far apart from each other and it is thus easy to set an activation threshold that will reliably activate the genuine units and deactivate the spurious units anyways. It is very unlikely that a small shift of the threshold will lead to an observable change in the number of activated units.

Although the above upper bound (Eq (S7.10)) is useful because it is a very simple expression, it relies on rather coarse approximations. In particular, the approximation for the spurious distribution in Eq (S7.7) can only be reached if $f_{\text{out}} = 0.5$ and a very large number of patterns P such that the genuine distribution is essentially the same as the spurious distribution already. We can more closely approximate the largest relevant value of the spurious probability function p_s by $\mathcal{P}(d_s = x_{f_{\text{out}}})$ with $F_s(x_{f_{\text{out}}}) = 1 - f_{\text{out}}$, where F_s is the cumulative distribution function of the spurious distribution. We find

$$F_s(x_{f_{\text{out}}}) = 1 - f_{\text{out}} \tag{S7.12}$$

$$\Leftrightarrow \frac{1}{2}\left(1 + \operatorname{erf}\left(\frac{x_{f_{\text{out}}} - \mu_s}{\sqrt{2}\sigma_s}\right)\right) = 1 - f_{\text{out}} \tag{S7.13}$$

$$\Leftrightarrow x_{f_{\text{out}}} = \mu_s + \sqrt{2}\sigma_s \operatorname{erf}^{-1}\left(1 - 2f_{\text{out}}\right) \tag{S7.14}$$

and hence

$$\mathcal{P}(d_s = x_{f_{\text{out}}}) = \frac{1}{\sigma_s\sqrt{2\pi}} \exp\left(-\frac{1}{2}\left(\frac{\mu_s + \sqrt{2}\sigma_s \operatorname{erf}^{-1}(1-2f_{\text{out}}) - \mu_s}{\sigma_s}\right)^2\right) \tag{S7.15}$$

$$= \frac{1}{\sigma_s\sqrt{2\pi} \cdot \exp\left(\left(\operatorname{erf}^{-1}(1-2f_{\text{out}})\right)^2\right)}. \tag{S7.16}$$

Together with the largest value of the genuine PDF (Eq (S7.5)), this gives the closer upper bound

$$M_{\text{err}} \leq \frac{1}{2}\left(\frac{M_{\text{out}}}{\sigma_g^{[P]}\sqrt{2\pi}} + \frac{N_{\text{out}} - M_{\text{out}}}{\sigma_s\sqrt{2\pi} \cdot \exp\left(\left(\operatorname{erf}^{-1}(1-2f_{\text{out}})\right)^2\right)}\right). \tag{S7.17}$$

Fig S7.1C compares the analytical upper bound of M_{err} (Eq (S7.17), dotted lines) to the actual error in activation ratio obtained in numerical simulations (solid lines). The numerical M_{err} was calculated by initializing a random weight matrix, presenting the network with a random input pattern with M_{in} active input units, and measuring the discretization error (Eq (S7.1)). It is observed that the normalized mean error (as well as its theoretical upper bound) decreases as a function of M_{in}. This is because higher M_{in} cause spurious and genuine distributions to be further apart, such that the number of units with dendritic sums close to the threshold T_{in} are lower.

Practical solutions

There are several approaches to handle the fact that it is often not possible to activate exactly M_{out} output units by choosing a fixed activation threshold T_{in}.

Random sampling of activated units. One way of achieving the exact given output sparseness is by randomly sampling some of the units that are activated. This method is used in all simulations of this paper unless stated otherwise (see Methods).

If the exact M_{out} cannot be achieved, this is because any possible activation threshold T_{in} yields too few or too many activated units. If the activation threshold was chosen as a, too few units would be activated, and if it was chosen as $a-1$, too many units would be activated. From all the output units that have the exact same dendritic sum (between $a-1$ and a), we randomly choose as many units as are needed to obtain M_{out} active units. Their activity is set to one while the activity of the rest of the units (with the same dendritic sum) is set to zero. This random choice constitutes an additional (but minor, see Fig S7.2) source of noise in the simulations.

Fixed noise mask on weight matrix. A fixed noise mask on the weight matrix represents an alternative solution. The choice of the activation threshold is ambiguous only because the input activities and the weight values are all zero or one. This yields relatively few distinct dendritic sums. If the dendritic sums were distributed continuously or with a finer discretization, the desired number of active output units M_{out} could be achieved more easily. The latter can, for example, be realized by point-wise multiplication of the weight matrix J with a fixed noise mask. The noise mask could be defined such that each entry is sampled from a normal distribution with mean $\mu = 1$ and a standard deviation $\sigma \ll 1$. Since weight values now have a small variability, it is much less probable that several output units receive the same dendritic sum and the activation threshold can be easily placed such that exactly M_{out} units are activated.

Fig S7.2 shows a comparison of average signal quality traces for the random sampling of activated units and a fixed noise mask on the weight matrix. When averaged across many patterns, there is hardly any difference between the two methods.

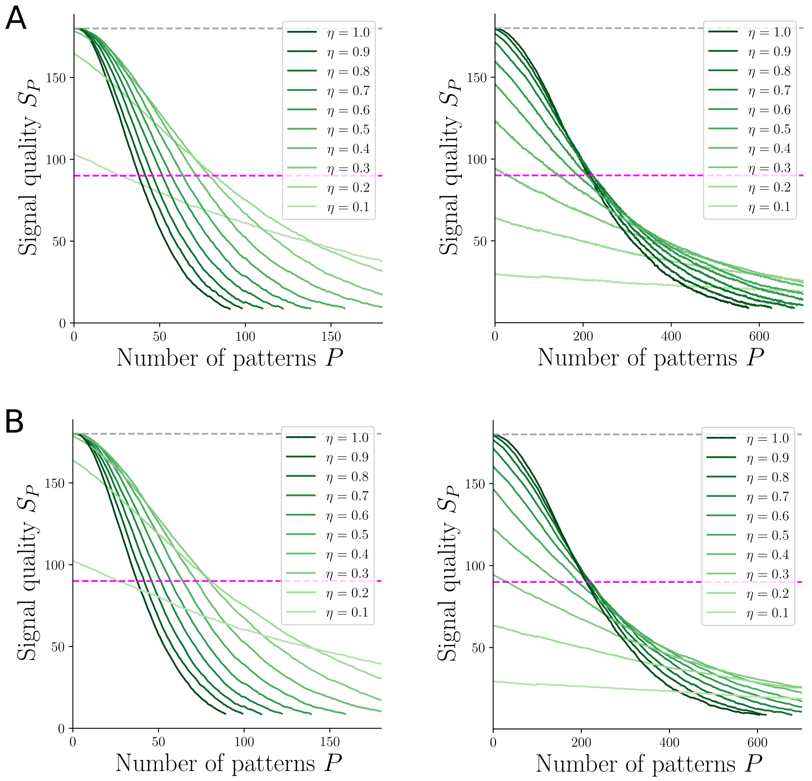

Fig S7.2. Random sampling of activated units or noise mask.
Comparison of signal quality for random sampling of activated units (**A**) and a fixed noise mask on the weight matrix (**B**) for two input activation ratios $f_{\text{in}} = 0.1$ (left) and $f_{\text{in}} = 0.012$ (right). Further parameters:
$N_{\text{in}} = N_{\text{out}} = 1000, f_{\text{out}} = 0.1, c = 0.2, c_m = 1, t_S = 0.5, N_{\text{avg}} = 200$.

A less strict f_{out}. Another obvious solution would be to implement a less strict output activation ratio f_{out}. Instead of enforcing an exact number of M_{out} active output units, there could be a certain tolerance of deviation from this value, e.g., $M_{\text{out}} \pm M_\varepsilon$ with a small $M_\varepsilon \in \mathbb{N}$. In principle, this is a reasonable solution but, since the work in this manuscript is focused on analyzing the impact of sparseness on the optimal transition probability, we want to enforce a fixed M_{out} and avoid such variabilities in activation ratios.
