# Supplementary material for: Population sparseness determines strength of Hebbian plasticity for maximal memory lifetime in associative networks
Source: PLoS Comput Biol. 2026 Jul 6;22(7):e1013235. doi: 10.1371/journal.pcbi.1013235 (PMC13390959; doi:10.1371/journal.pcbi.1013235)
Supplement: S8 Appendix — Translation of main results from pattern capacity to an information-theoretic measure of synaptic capacity. (PDF) [file pcbi.1013235.s014.pdf]

S8 Appendix

Information-theoretic synaptic capacity

This appendix provides an extension of our description of the memory capacity P^*, which was defined in Eq (8) in the Results section of the manuscript. P^* will be called *pattern capacity* in the following to emphasize that it represents a number of patterns. In this appendix, we calculate the information-theoretic *synaptic capacity* (see, e.g., [1, 2]) and show results of numerical simulations that translate our main results from Fig 3 of the manuscript into this measure. As shown in detail in what follows, the two capacity measures show a remarkably similar dependence on the activation ratios f_{in} and f_{out} and the transition probability η.

Synaptic capacity takes into account the information content of the stored patterns and the storage resources in terms of synaptic connections. Synaptic capacity has the unit of bits per synapse and is defined here as the mutual information between the target output patterns and the retrieved output patterns, normalized by the number of functional connections in the network [2, 3]:

$$C^{\infty} = \frac{\mathcal{I}(\mathbf{y}^{[1]}, \mathbf{y}^{[2]}, \mathbf{y}^{[3]}, \ldots; \hat{\mathbf{y}}^{[1]}, \hat{\mathbf{y}}^{[2]}, \hat{\mathbf{y}}^{[3]}, \ldots)}{cN_{\text{in}}N_{\text{out}}} \in [0, 1] \tag{S8.1}$$

Mutual information of target patterns and calculated output patterns

Since the learned patterns are assumed to be statistically independent, the numerator of Eq (S8.1) is a sum of the mutual information terms for the single patterns. Therefore, we first discuss the mutual information $\mathcal{I}(\mathbf{y}; \hat{\mathbf{y}}(P))$ between one target output pattern $\mathbf{y}$ and its retrieved counterpart $\hat{\mathbf{y}}(P)$, which depends on the number P of subsequently learned patterns. Assuming the statistical independence of output units, the mutual information of one pattern can be expressed as

$$\mathcal{I}(\mathbf{y}; \hat{\mathbf{y}}(P)) = N_{\text{out}}\mathcal{I}(y; \hat{y}(P)) \tag{S8.2}$$

where $\mathcal{I}(y; \hat{y}(P))$ is the mutual information of a single unit, which is defined as

$$\mathcal{I}(y; \hat{y}) = \mathcal{H}(\hat{y}) - \mathcal{H}(\hat{y}|y) \tag{S8.3}$$

where $\mathcal{H}(\hat{y})$ is the entropy of $\hat{y}$ and $\mathcal{H}(\hat{y}|y)$ is the conditional entropy of $\hat{y}$ given y. For simplicity, we omitted the explicit parameter P in the notation and include it only again in the end. In the following, we use the notation

$$\mathcal{H}_b(p) := -p\log_2(p) - (1-p)\log_2(1-p) \tag{S8.4}$$

for the entropy of a Bernoulli process with probabilities p and $1 - p$. Since the output units are binary and the output activation ratio is fixed to f_{out}, we have

$$\mathcal{H}(\hat{y}) = \mathcal{H}_b(f_{\text{out}}). \tag{S8.5}$$

The conditional entropy is defined as

$$\mathcal{H}(\hat{y}|y) = \sum_{a\in\{0,1\}} \mathcal{P}(y = a)\mathcal{H}(\hat{y}|y = a). \tag{S8.6}$$

For $y = 1$, we have

$$\mathcal{P}(y = 1) = f_{\text{out}} \tag{S8.7}$$

and, since

$$\mathcal{P}(\hat{y}=0|y=1) = e_{\text{fn}}, \tag{S8.8}$$
$$\mathcal{P}(\hat{y}=1|y=1) = 1-e_{\text{fn}}, \tag{S8.9}$$

we obtain

$$\mathcal{H}(\hat{y}|y=1) = \mathcal{H}_b(e_{\text{fn}}), \tag{S8.10}$$

where e_{fn} is the probability of a falsely inactive output unit (false negative). Analogously, for $y=0$, we have

$$\mathcal{P}(y=0) = 1-f_{\text{out}} \tag{S8.11}$$

and, since

$$\mathcal{P}(\hat{y}=1|y=0) = e_{\text{fp}}, \tag{S8.12}$$
$$\mathcal{P}(\hat{y}=0|y=0) = 1-e_{\text{fp}}, \tag{S8.13}$$

we obtain

$$\mathcal{H}(\hat{y}|y=0) = \mathcal{H}_b(e_{\text{fp}}), \tag{S8.14}$$

where e_{fp} is the probability of a falsely active output unit (false positive). The conditional entropy is then

$$\mathcal{H}(\hat{y}|y) = f_{\text{out}}\mathcal{H}_b(e_{\text{fn}}) + (1-f_{\text{out}})\mathcal{H}_b(e_{\text{fp}}). \tag{S8.15}$$

The mutual information for the whole pattern is then

$$\mathcal{I}(\mathbf{y};\hat{\mathbf{y}}(P)) = N_{\text{out}}\left[\mathcal{H}_b(f_{\text{out}}) - f_{\text{out}}\mathcal{H}_b(e_{\text{fn}}) - (1-f_{\text{out}})\mathcal{H}_b(e_{\text{fp}})\right]. \tag{S8.16}$$

The error probabilities e_{fn} and e_{fp} in these equations are defined through the cumulative distribution functions of the dendritic sums (see Section 'Theory on distributions of dendritic sums and calculation of capacity' in the Results) and can be expressed in terms of the normalized signal quality s_P (see Section 'Signal quality after P subsequent patterns' in the Methods, in particular Fig 12):

$$e_{\text{fn}} = F_g(T_{\text{in}}) = (1-s_P)(1-f_{\text{out}}) \tag{S8.17}$$
$$e_{\text{fp}} = 1-F_s(T_{\text{in}}) = (1-s_P)f_{\text{out}} \tag{S8.18}$$

Together, the mutual information for one pattern is

$$\begin{aligned}&\mathcal{I}(\mathbf{y};\hat{\mathbf{y}}(P)) \\ &\quad = N_{\text{out}}\left[\mathcal{H}_b(f_{\text{out}}) - f_{\text{out}}\mathcal{H}_b((1-s_P)(1-f_{\text{out}})) - (1-f_{\text{out}})\mathcal{H}_b((1-s_P)f_{\text{out}})\right],\end{aligned} \tag{S8.19}$$

where the dependence on the number P of subsequent patterns is inherent in s_P. The normalized signal quality $s_P = S_P/T_S$ is defined via the Hamming distance between target output and calculated output (see Eq (7) in 'Network model, learning paradigm, and quantification of capacity'). An analytical approximation of s_P can be found in Eq (S3.23) in S3 Appendix. Fig S8.1A shows the mutual information of a pattern (Eq (S8.19)) as a function of the number P of subsequently learned patterns for several transition probabilities η.

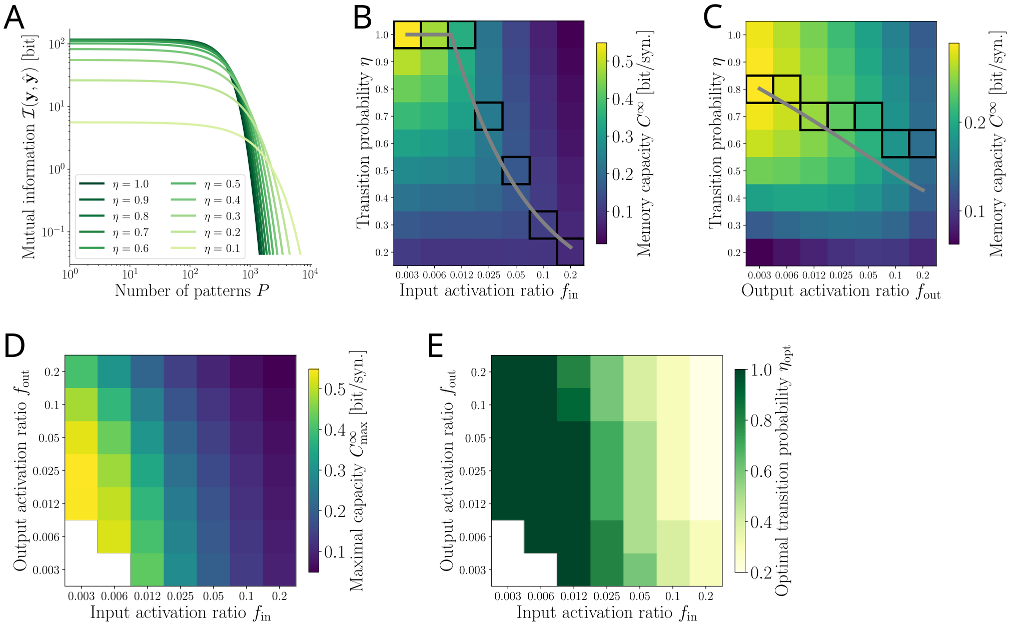

Fig S8.1. Synaptic capacity of a sequence of patterns.
(**A**) The mutual information $\mathcal{I}(\mathbf{y}; \hat{\mathbf{y}}(P))$ of a target pattern $\mathbf{y}$ and the corresponding retrieved output pattern $\hat{\mathbf{y}}$ decreases as a function of the number P of subsequently learned patterns. The initial mutual information (at $P = 0$) is larger for higher transition probabilities η, whereas the decrease is slower for lower η. Fixed parameters: $f_{\text{in}} = f_{\text{out}} = 0.025$. (**B**) For a given transition probability $\eta \leq 1$, the memory capacity increases with decreasing input activation ratio f_{in}. The optimal η, which is defined as the transition probability that results in the largest memory capacity for a given f_{in}, increases with decreasing f_{in} until it reaches and stays at its largest value of 1 (black squares). The solid gray line is the same as in Fig 3B and shows the analytically derived optimal η for the pattern capacity as a function of f_{in}. It is close to the black squares and shows a similar trend. Fixed parameter: $f_{\text{out}} = 0.025$. (**C**) Same as (**B**) for output activation ratio f_{out} instead of input activation ratio f_{in}. The memory capacity and the optimal η increase with decreasing f_{out}. Fixed parameter: $f_{\text{in}} = 0.025$. In (**D**), the maximal capacity (obtained with the optimal transition probability η_{opt} shown in (**E**)) for each combination of input activation ratio f_{in} and output activation ratio f_{out} is shown. For a fixed f_{out}, the maximal capacity decreases as a function of f_{in}. For a fixed f_{in}, the maximal capacity decreases as a function of f_{out}. (**E**) Optimal transition probability η_{opt} increases with decreasing f_{in} and with decreasing f_{out}. The slope is larger for f_{in} than f_{out}. White areas in (**D**) and (**E**) are left empty due to very long simulation times. Further fixed parameters in (**A**)–(**E**): $N_{\text{in}} = 1000$, $N_{\text{out}} = 1000$, $c = 0.2$, $c_m = 1$.

Capacity of a sequence of patterns

It is generally a limitation of the pattern capacity P^* as defined in this work that the retrieval threshold T_S can be chosen arbitrarily. The synaptic capacity can, however, be analyzed independently of such an arbitrary threshold by quantifying the mutual information of an entire sequence of patterns. Even from pattern pairs that were learned so long ago that the signal quality is lower than the imposed retrieval threshold, some (small but non-zero) mutual information remains stored in the connection matrix.

The mutual information of an infinite sequence of patterns is defined as

$$\mathcal{I}^{\infty} = \sum_{P=0}^{\infty} \mathcal{I}(\mathbf{y}; \hat{\mathbf{y}}(P)). \quad \text{(S8.20)}$$

The synaptic capacity can then be expressed as

$$C^{\infty} = \frac{\sum_{P=0}^{\infty} \mathcal{I}(\mathbf{y}; \hat{\mathbf{y}}(P))}{cN_{\text{in}}N_{\text{out}}} = \frac{N_{\text{out}} \sum_{P=0}^{\infty} \mathcal{I}(y; \hat{y}(P))}{cN_{\text{in}}N_{\text{out}}} \quad \text{(S8.21)}$$

$$= \frac{\sum_{P=0}^{\infty} [\mathcal{H}_b(f_{\text{out}}) - f_{\text{out}} \mathcal{H}_b((1-s_P)(1-f_{\text{out}})) - (1-f_{\text{out}})\mathcal{H}_b((1-s_P)f_{\text{out}})]}{cN_{\text{in}}}, \quad \text{(S8.22)}$$

where we have used the definitions in Eq (S8.2) and Eq (S8.19). The synaptic capacity C^{∞} can be interpreted as a scaled version of the areas under the curves (for $P \in [0, \infty)$) in Fig S8.1A.

The numerator of C^{∞} virtually depends on all model parameters, in particular on the transition probability η and on the input and output activation ratios f_{in} and f_{out}. In Fig S8.1, we provide a numerical analysis of the optimal transition probability η_{opt} and the maximal synaptic capacity for an example set of parameters (same as for the pattern capacity in Fig 3 of the main manuscript). Therefore, we approximate C^{∞} by semi-analytically calculating s_P as explained in Section 'Numerical calculation of the signal quality' of S3 Appendix and then summing all contributions where $s_P > 10^{-2}$. As in Fig 3, the optimal transition probability η_{opt} decreases as a function of the input activation ratio f_{in} (Fig S8.1B) and as a function of the output activation ratio f_{out} (Fig S8.1C), with a steeper gradient for f_{in} (see also Fig S8.1E). The optimal transition probabilities η_{opt} obtained from the pattern capacity P^* (gray curves in Fig S8.1B,C) slightly underestimate the optimal transition probabilities η_{opt} obtained from the synaptic capacity C^{∞} (black squares in Fig S8.1B,C). For the parameters investigated here, the maximal capacity C^{∞}_{max}, i.e., the capacity obtained with the optimal transition probability, decreases as a function of both f_{in} and f_{out} (Fig S8.1D). A more detailed analysis of the relationship between activation ratios, transition probability, and synaptic capacity remains a subject for future work.

References

1. Palm G. On associative memory. Biological Cybernetics. 1980;36(1):19–31.
2. Knoblauch A, Palm G, Sommer FT. Memory capacities for synaptic and structural plasticity. Neural Computation. 2010;22(2):289–341.
3. Knoblauch A. Neural associative memory and the Willshaw–Palm probability distribution. SIAM Journal on Applied Mathematics. 2008;69(1):169–196.
